# The impact of sulfadoxine–pyrimethamine resistance on the effectiveness of intermittent preventive treatment for the prevention of malaria in pregnancy in Africa: an updated systematic review and meta-analysis

**DOI:** 10.1016/S1473-3099(25)00219-1

**Published:** 2025-07-14

**Authors:** Anna Maria van Eijk, Kasia Stepniewska, Carole Khairallah, Eva Rodriguez, Jordan Ahn, Julie R Gutman, Feiko O ter Kuile, for the IPTp-SP Effectiveness Study group

**Affiliations:** Department of Clinical Sciences, Liverpool School of Tropical Medicine, Liverpool, UK; Centre for Tropical Medicine and Global Health, Nuffield Department of Clinical Medicine, University of Oxford, Oxford, UK; Department of Clinical Sciences, Liverpool School of Tropical Medicine, Liverpool, UK; Emory University, Atlanta, GA, USA; Emory University, Atlanta, GA, USA; Malaria Branch, Division of Parasitic Diseases and Malaria, Centers for Disease Control and Prevention, Atlanta, GA, USA; Department of Clinical Sciences, Liverpool School of Tropical Medicine, Liverpool, UK

## Abstract

**Background:**

Resistance of *Plasmodium falciparum* to sulfadoxine–pyrimethamine threatens the antimalarial effectiveness of intermittent preventive treatment during pregnancy (IPTp) with sulfadoxine–pyrimethamine (ITPp-SP) in sub-Saharan Africa. We updated an aggregated-data meta-analysis to assess the associations between sulfadoxine–pyrimethamine resistance and the effectiveness of IPTp-SP to inform policy.

**Methods:**

We searched databases (Jan 1, 1990, to June 8, 2024) for observational studies or trials reporting data on malaria, low birthweight (<2500 g), anaemia, and other outcomes by IPTp-SP dose and matched these by year and location with studies that reported on molecular markers of sulfadoxine–pyrimethamine resistance. Studies including only women with HIV or combined interventions were excluded. We evaluated how sulfadoxine–pyrimethamine resistance influenced the adjusted risk ratio (aRR) between three and two doses of IPTp-SP for various outcomes using Poisson mixed-effects models that allowed for non-linear relationships. Initially, we performed a threshold analysis, stratified by region, to identify the resistance levels most predictive of altered effect of IPTp-SP doses on malaria parasitaemia at delivery (peripheral or placental parasitaemia by any test), our primary outcome. These resistance strata were then used in all subsequent models for other outcomes. All analyses were adjusted for malaria transmission intensity, HIV infection, percentage of paucigravidae, and insecticide-treated net use. Performance of models was evaluated using cross-validation. The trial was registered with PROSPERO (CRD42021250359).

**Findings:**

Overall, 122 studies involving 148 693 participants were included. For west and central Africa (69 studies comprising 63 745 participants), very low resistance was categorised as a prevalence of the dihydropteroate synthase (*dhps*) Lys540Glu mutation in the parasite population of less than 4%, and low resistance as a prevalence of Lys540Glu of 4% or higher. In east and southern Africa (53 studies comprising 84 948 participants), moderate resistance was categorised as a prevalence of the Lys540Glu mutation of less than 60% combined with a prevalence of the Ala581Gly mutation of less than 5%, high resistance as a prevalence of Lys540Glu of 60% or higher combined with a prevalence of Ala581Gly of less than 5%, and very high resistance as a prevalence of the Lys540Glu mutation of 60% or higher combined with a prevalence of Ala581Gly of 5% or higher. There was a marked trend towards lower efficacy of IPTp-SP on reducing malaria infection with increasing resistance levels. In west and central Africa, when comparing three versus two doses, the aRR was 0·71 (95% CI 0·65–0·78) in areas with very low resistance and 0·83 (0·72–0·95) in areas with low resistance (p=0·0144 for the difference between dose–response curves in very low *vs* low resistance). For east and southern Africa, the same trend was observed: the aRR was 0·63 (95% CI 0·57–0·69) in areas with moderate resistance, 0·89 (0·82–0·96) in areas with high resistance, and 0·93 (0·85–1·01) in areas with very high resistance (p<0·0001 for dose–response curves differences between moderate *vs* high and moderate *vs* very high resistance). This pattern was not seen for low birthweight. When comparing three versus two doses in west and central Africa, the aRR was 0·58 (95% CI 0·48–0·68) in areas with very low resistance and 0·56 (0·44–0·68) in areas with low resistance (p=0·72 for dose–response curves very low *vs* low resistance). For east and southern Africa, the aRR was 0·75 (95% CI 0·52–0·98) in areas with moderate resistance, 0·73 (0·69–0·78) in areas with high resistance, and 0·75 (0·63–0·87) in areas with very high resistance (p=0·80 for dose–response curves moderate *vs* high resistance; p=0·90 for moderate *vs* very high resistance). Dose comparisons in some resistance strata were limited by sample size.

**Interpretation:**

IPTp-SP antimalarial efficacy is greatly reduced in very high resistance areas. However, it remains effective at reducing low birthweight in these areas, possibly through non-malaria effects on fetal growth. While IPTp-SP use should continue in high SP-resistance areas, alternative malaria preventive strategies are urgently needed in these areas.

**Funding:**

WHO and WorldWide Antimalarial Resistance Network.

## Introduction

Approximately 12·4 million pregnant women in malaria-endemic regions in Africa were infected with malaria in 2023.^1^ Malaria in pregnancy is associated with maternal anaemia and clinical illness, pregnancy loss, fetal growth restriction and preterm birth resulting in low birthweight (<2500 g).^2^

Since the 1980s, chemoprevention in pregnancy has been used to reduce the adverse effects of malaria in pregnant women and their newborns. WHO has recommended intermittent preventive treatment in pregnancy (IPTp) with sulfadoxine–pyrimethamine (IPTp-SP) since 1998; in contrast to chemoprophylaxis, IPTp consists of full treatment doses of long-acting antimalarials at regular intervals. In 2012, WHO recommended that IPTp-SP be given to all women in their first or second pregnancy (paucigravidae) in malaria-endemic areas of Africa as a part of antenatal care, starting as early as possible in the second trimester and continuing until delivery, with each treatment dose of sulfadoxine–pyrimethamine (three tablets) given at least one month apart.^3–5^ In 2022, the recommendation was made explicit for women of all pregnancies, although this approach had been standard practice in Africa for many years.^6^

A 2014 meta-analysis which included 17 randomised controlled trials (RCTs) and quasi-RCTs of antimalarials versus placebo or no intervention showed that among paucigravidae, malaria chemoprevention in pregnancy reduced the risk of antenatal and placental parasitaemia, maternal anaemia, and low birthweight.^7^ Among women in their third or higher pregnancy (multigravidae), chemoprevention reduced antenatal parasitaemia, but there were too few trials to evaluate the effects on other outcomes.^7^ An analysis of national cross-sectional surveys published in 2012, including 32 surveys from 25 African countries, demonstrated that compared to 0 or 1 dose of sulfadoxine–pyrimethamine, receipt of at least two doses of IPTp-SP was associated with a statistically significant reduction in low birthweight, with a similar effect size in paucigravidae (20·2%) and multigravidae (21·5%).^8^ Concerns over *Plasmodium falciparum* resistance to sulfadoxine–pyrimethamine have led to trials assessing the impact of increasing the number of IPTp doses. A 2013 meta-analysis, including data from seven trials, found that three or more doses of IPTp-SP were associated with significant reductions in maternal and placental parasitaemia and higher mean birthweight compared to two doses in both paucigravidae and multigravidae.^9^

Sulfadoxine–pyrimethamine resistance results from a series of single nucleotide polymorphisms in the dihydrofolate reductase gene ([*dhfr*] substitutions Asn51Ile, Cys59Arg, and Ser108Asn) and dihydropteroate synthase gene ([*dhps*] substitutions Ala437Gly, Lys540Glu, and Ala581Gly) of *P falciparum*. The intensity of resistance to sulfadoxine–pyrimethamine increases with the number and types of mutant codons, with quintuple mutations (five mutations including Lys540Glu, excluding Ala581Gly) being associated with mid-level resistance, and sextuple mutations (six mutations including Ala581Gly) with high-level resistance. Chico and colleagues conducted a systematic review and meta-analysis in 2015, pairing data from IPTp trials together with data on prevalence of sulfadoxine–pyrimethamine resistance markers, and found that a prevalence of over 10·1% of the highly resistant sextuple mutant parasites harbouring the Ala581Gly mutation was associated with reduced sulfadoxine–pyrimethamine efficacy.^10^ In 2019, van Eijk and colleagues further evaluated the effect of sulfadoxine–pyrimethamine resistance on the effectiveness of IPTp on low birthweight, and other outcomes, in a meta-analysis that included aggregated data published from Jan 1, 1990, to March 1, 2018.^11^ IPTp-SP remained associated with reductions in low birthweight even in areas where a substantial proportion of parasites harboured the Ala581Gly mutation, albeit the effect was smaller than in areas with less resistance.^11^ Here, we update the 2019 review by including additional data obtained from studies published since March 1, 2018 and older studies and revising the methodology to better take into account a non-linear relationship between sulfadoxine–pyrimethamine doses and outcomes.^11^

## Methods

### Search strategy and selection criteria

Both randomised and non-randomised studies with data on pregnancy outcomes by the number of sulfadoxine–pyrimethamine doses were included (search period: Jan 1, 1990, to June 8, 2024). Outcomes of interest included *Plasmodium* spp infection at delivery (maternal or placental blood by any test), maternal haemoglobin or anaemia (haemoglobin <11 g/dL), birthweight and low birthweight (<2500 g), premature delivery (<37 weeks), and gestational age at the time of delivery. Studies which only included HIV-positive women, studies outside of Africa, and study groups where sulfadoxine–pyrimethamine was combined with another drug were excluded. For further details on search strategy and outcomes, see the appendix (pp 5–7). This review followed the PRISMA guidelines. The study is registered with PROSPERO (CRD42021250359).

### Extraction and quality assessment of IPTp effectiveness data

Two teams of two investigators each (AMvE and JA, and ER and JRG) independently extracted aggregated data on outcomes by sulfadoxine–pyrimethamine dose. Authors of primary studies were contacted for missing information or if reported data did not fit the required format. The same two teams independently assessed study quality using an adaptation of the Newcastle–Ottawa Scale that expanded exposure ascertainment criteria to include antenatal records and modified comparability assessment to examine differences in characteristics between sulfadoxine–pyrimethamine dose groups (appendix pp 7–8).^12^ Data on the prevalence of *dhps* Ala437Gly, Lys540Glu, and Ala581Gly mutations among *P falciparum* parasites were extracted from the clinical studies in pregnant women (if provided), and otherwise from the literature or existing molecular surveyor databases. Further details on matching sulfadoxine–pyrimethamine resistance markers and malaria transmission levels and quality assessment are provided in the appendix (pp 9–10).

### Statistical analysis

To define thresholds for resistance, we used the outcome of any malaria at delivery, defined as placental or maternal malaria detected by any diagnostic test at delivery. Threshold analyses were done separately for central and west Africa and east and southern Africa because of distinct parasite populations and distributions of mutations in each region.^13,14^ For each combination of the thresholds and combinations of the different markers, mixed-effects Poisson models were fitted to the number of malaria cases, with the total number of participants (ie, the denominator) as the exposure variable (appendix p 10). Study site was used as a cluster variable, and random intercept and slope for sulfadoxine–pyrimethamine dose were included in the model since they consistently gave lower Akaike information criterion (an indicator of the fit of the model) than corresponding models with random intercept only. The dose was modelled after the approximate cumulative distribution transformation to allow for the S-shape dose–response relationship.^15^ Other non-linear relationships were explored using fractional polynomials, but approximate cumulative distribution transformation provided the best fit to the data. Interactions between the transformed dose variable and the resistance variable categories were included in the model so that the separate dose–response relationship was fitted for each of the resistance categories. The resistance variable was included in the model only through the interaction terms (no main effects), as it was assumed that the only association between the resistance variable and the outcome (percentage with outcome of interest) was via the possibly altered efficacy of the treatment (changed dose-effect relationship). All models were adjusted for the study-level co-variables of malaria transmission intensity (*Pf*PR_2–10_), estimated HIV infection prevalence, percentage of paucigravidae in the study, and insecticide-treated net use.^11^ Performance of models was evaluated using cross-validation. Each study was excluded in turn, and the model was fitted to the data from the remaining studies, and the prevalence of malaria cases was predicted for the excluded study. The mean value of Akaike information criterion and root-mean square error were used to assess the performance.

The same approximate cumulative distribution transformation was used for peripheral and placental parasitaemia outcomes. Using fractional polynomials, the best non-linear models were identified for groups of other outcomes (low birthweight, prematurity, birthweight and gestational age combined, and haemoglobin and anaemia combined). Graphs were prepared showing the effect of sulfadoxine–pyrimethamine dose on each outcome by resistance level in each region, using two sulfadoxine–pyrimethamine doses as the reference, showing the adjusted risk ratio or adjusted mean difference by different resistance strata. We used a Wald test to assess differences between non-linear effects of our interaction terms (representing sulfadoxine–pyrimethamine dose-resistance category interactions) in our mixed-effects Poisson regression model, using Stata’s post-estimation test command. Additionally, we conducted pair-wise comparisons of the effects of consecutive sulfadoxine–pyrimethamine dose levels (3 *vs* 2 doses, 4 *vs* 3 doses, and 5 *vs* 4 doses) for each outcome. These comparisons were also derived from the mixed-effects Poisson regression models, employing both conditional and marginal models, where sufficient data were available. This analysis allowed us to quantify the incremental impact of each additional sulfadoxine–pyrimethamine dose on the outcomes of interest. Analyses were repeated by gravidity for malaria and low birthweight outcomes. Further details of the methodology are available in the appendix (pp 10–12). We used a two-sided p value <0·05 to define statistical significance. All analyses were conducted with Stata version 17.

### Role of the funding source

The funders had no role in the study design, data collection, analysis, interpretation, or writing of this manuscript. The corresponding author had full access to all the data in the study and together with the joint senior authors had final responsibility for the decision to submit for publication.

## Results

**Figure 1:**
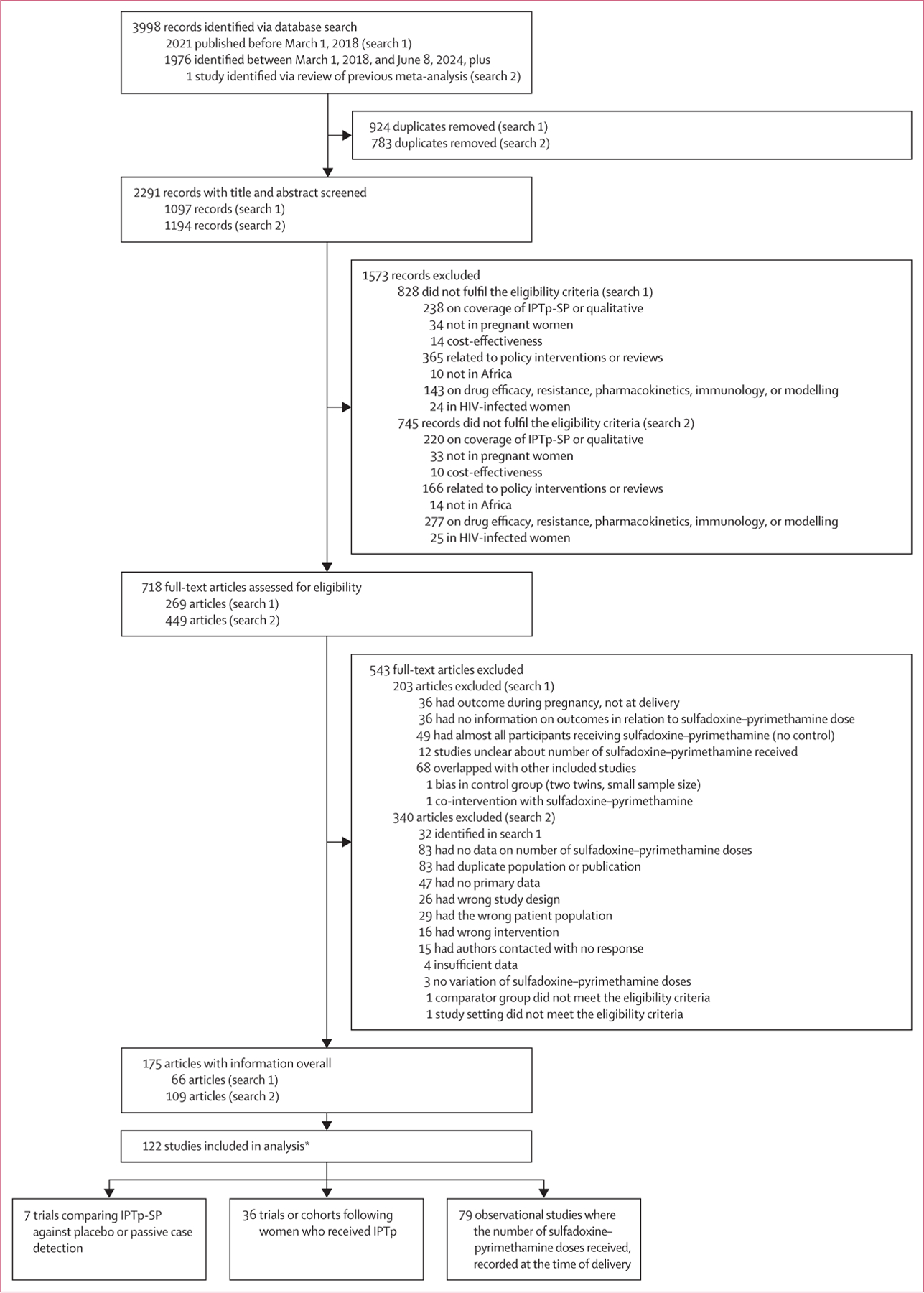
PRISMA diagram IPTp=intermittent preventative treatment in pregnancy. IPTp-SP=IPTp with sulfadoxine –pyrimethamine. *The difference between 175 articles and 122 studies reflects that data from several studies were reported in multiple publications.

Overall, 3998 records were identified through database searching, and 122 studies fulfilled the eligibility criteria and were included in the review: 69 from west and central Africa and 53 from east and southern Africa, involving 148 693 participants (63 745 in west and central Africa and 84 948 in east and southern Africa). These included seven trials comparing IPTp-SP against placebo or passive case detection, 366 trials or cohorts following women who received IPTp-SP, and 79 cross-sectional studies (figure 1, appendix pp 13, 40). The studies were conducted between 1993 and 2021. Most studies were classified as of moderate-to-low quality (89 [73%] of 122 had a quality score of ≤4 out of 6 points, appendix pp 8–9, 13–19). The relationship between the *dhps* Ala437Gly and *dhps* Lys540Glu mutation in the study locations was similar to our previous publication, and differed strongly by region (appendix p 41).^11^ The match between clinical studies and sulfadoxine–pyrimethamine resistance marker data was optimal for most locations. Only five of 84 locations (6%) in west and central Africa and one of 59 locations (2%) in east and southern Africa had suboptimal matches (defined as temporal difference ≥3 years, geographical distance >300 km, or molecular marker sample size <30, appendix pp 10, 20–26). Using threshold analysis, for west and central Africa, very low and low resistance were categorised as a prevalence of the Lys540Glu mutation under 4% and at 4% or above, respectively. For east and southern Africa, moderate resistance was categorised as a combined prevalence of Lys540Glu under 60% and Ala581Gly under 5%, high resistance as a combined prevalence of Lys540Glu at 60% or above and Ala581Gly under 5%, and very high resistance as a combined prevalence of Lys540Glu at 60% or above and Ala581Gly at 5% or above.

**Figure 2:**
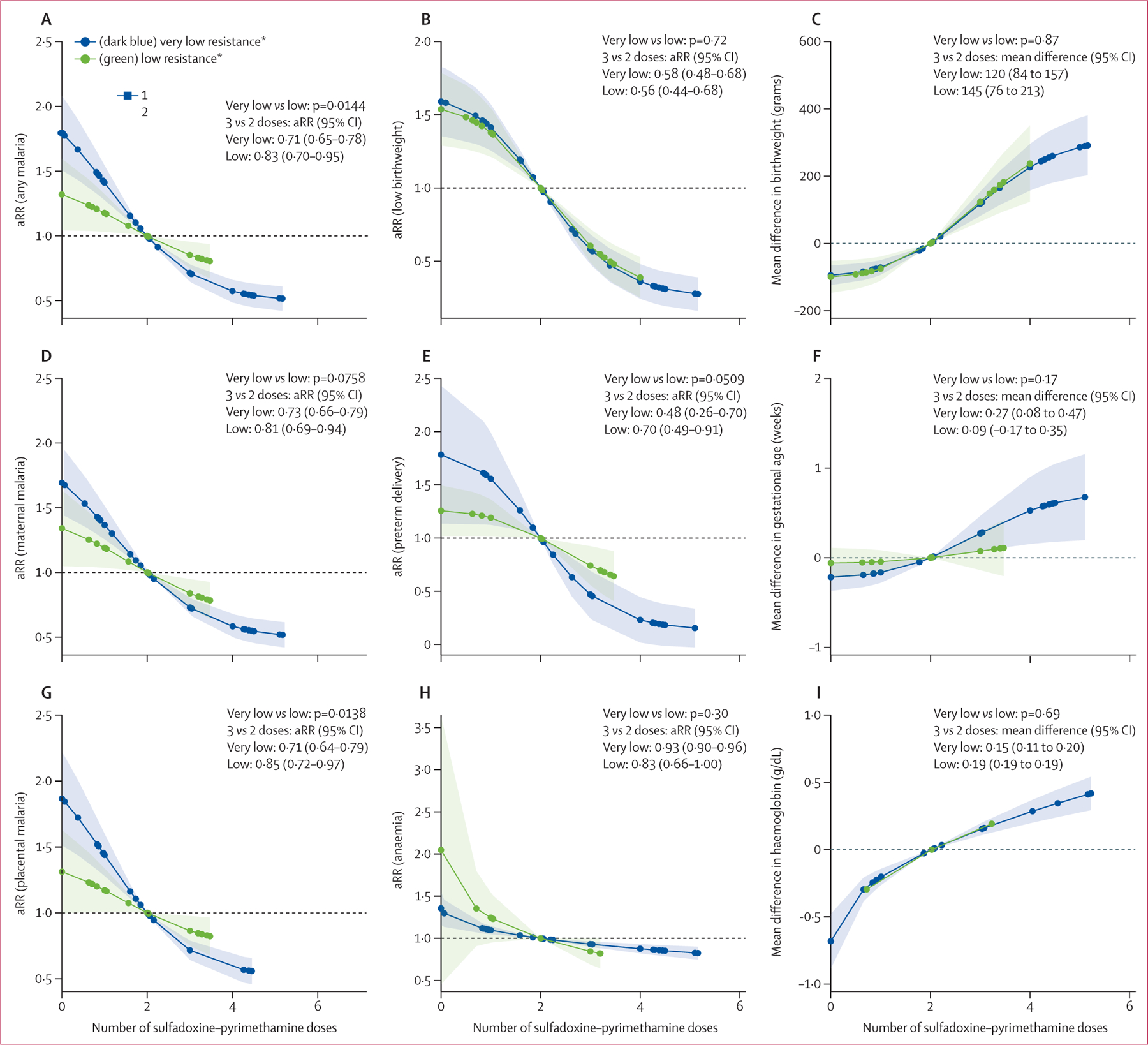
aRRs in west and central Africa associated with IPTp-SP by resistance strata*, 1993–2021 (A) Adjusted risk ratio for any malaria; (B) adjusted risk ratio for low birthweight; (C) mean difference in birthweight; (D) adjusted risk ratio for maternal malaria; (E) adjusted risk ratio for preterm delivery; (F) mean difference in gestational age; (G) adjusted risk ratio for placental malaria; (H) adjusted risk ratio for anaemia; and (I) mean difference in haemoglobin. Two doses of sulfadoxine–pyrimethamine used as the reference dose. All given p values reflect comparisons between dose–response curves across resistance strata, not individual effect estimates of three versus two doses. For full details see appendix (pp 27–29). Shaded areas indicate 95% CIs. aRR=adjusted risk ratio for the effect of three versus two doses for each resistance strata. IPTp-SP=intermittent preventive treatment in pregnancy with sulfadoxine–pyrimethamine. *Very low resistance was categorised as Lys540Glu under 4% (dark blue), and low resistance was categorised as Lys540Glu at 4% or above (green).

**Figure 3:**
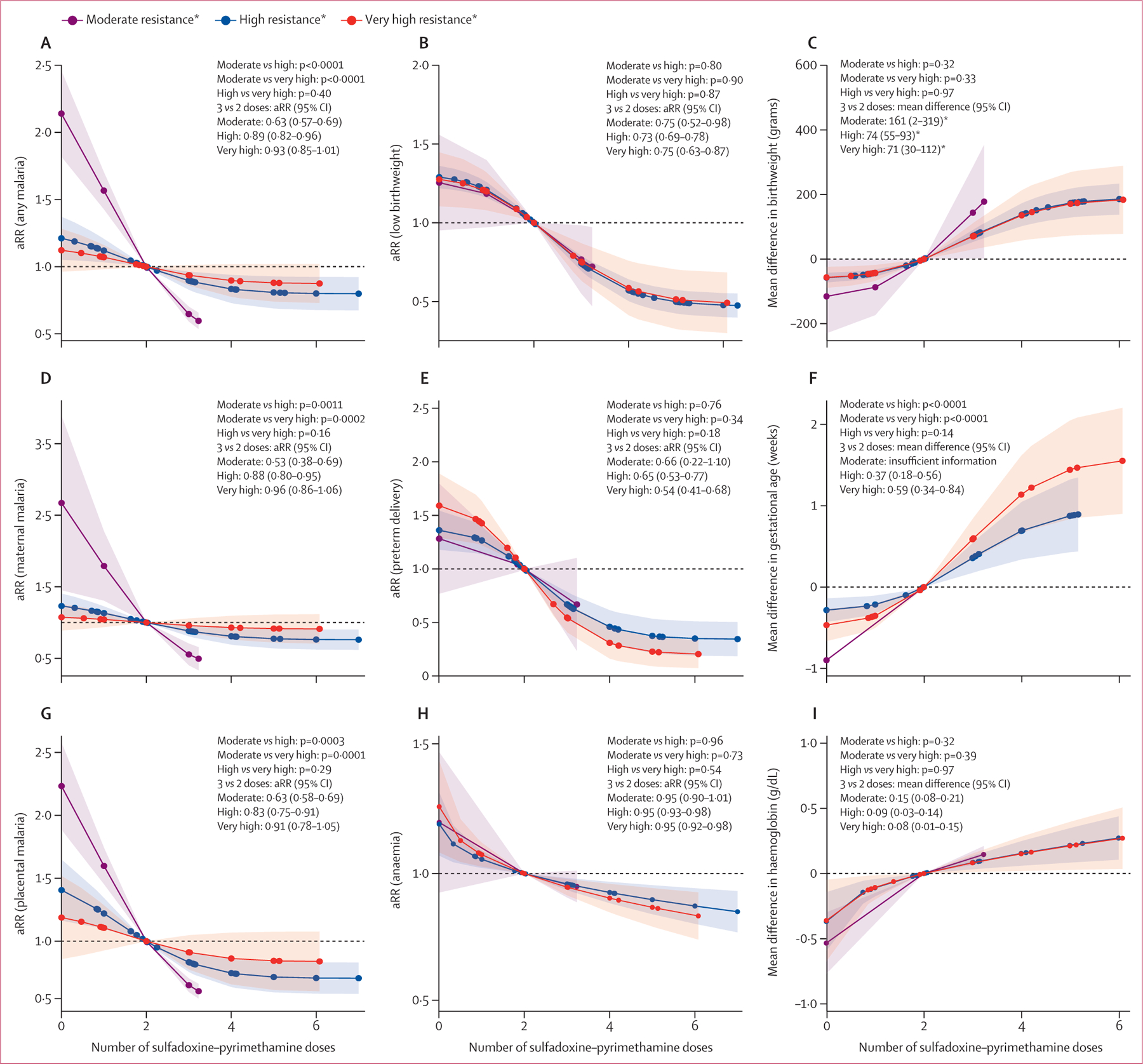
aRRs of outcomes in east and southern Africa associated with IPTp-SP by resistance strata*, 1993–2021 (A) Adjusted risk ratio for any malaria; (B) adjusted risk ratio for low birthweight; (C) mean difference in birthweight; (D) adjusted risk ratio for maternal malaria; (E) adjusted risk ratio for preterm delivery; (F) mean difference in gestational age; (G) adjusted risk ratio for placental malaria; (H) anaemia; and (I) mean difference in haemoglobin. Two doses of sulfadoxine–pyrimethamine used as the reference dose. All given p values reflect comparisons between dose–response curves across resistance strata, not individual effect estimates of three versus two doses. Shaded areas indicate 95% CIs. aRR=adjusted risk ratio for the effect of three versus two doses for each resistance strata. IPTp-SP=intermittent preventive treatment in pregnancy with sulfadoxine–pyrimethamine. *Moderate resistence was categorised as Lys540Glu under 60% and Ala581Gly under 5% (purple), high resistance as Lys540Glu at 60% or above and Ala581Gly under 5% (blue), and very high resistance as Lys540Glu at 60% or above and Ala581Gly at 5% or above (red).

The effectiveness of sulfadoxine–pyrimethamine in reducing malaria infection outcomes at delivery (including any malaria and maternal and placental parasitaemia) decreased as the parasite resistance level increased in both regions (figures 2 and 3). In areas of west and central Africa with very low to low resistance, sulfadoxine–pyrimethamine effectively decreased malaria infection. This protective effect had a dose–response relationship, with the magnitude of protection increasing with each additional dose of sulfadoxine–pyrimethamine, albeit with diminishing returns at higher doses (figure 2 and appendix pp 27–28). In west and central Africa, the protective effect of three versus two doses of sulfadoxine–pyrimethamine on any malaria infection at delivery persisted in areas with low resistance (adjusted risk ratio [aRR] 0·83 [95% CI 0·72–0·95] for very low resistance *vs* 0·71 [0·65–0·78] for low resistance), despite an attenuation of the dose–response relationship (p=0·0144 for dose–response curve comparison; figure 2). In east and southern Africa, the effectiveness of sulfadoxine–pyrimethamine markedly declined in areas with high and very high levels of parasite resistance relative to moderate transmission areas, depicted by a notable flattening of the dose–response curve in these areas compared with the moderate resistance areas (figure 3). In east and southern Africa, the aRR for the effect of three versus two doses of IPTp-SP on any malaria infection at delivery was 0·63 (95% CI 0·57–0·69), 0·89 (0·82–0·96) and 0·93 (0·85–1·01) in moderate, high, and very high resistance areas, respectively (p<0·0001 for the dose–response curves comparison moderate *vs* high and moderate *vs* very high resistance strata; p=0·40 for dose–response curves comparison; figure 3 and appendix pp 27–28).

In contrast to the observed effect of sulfadoxine–pyrimethamine resistance on malaria infection outcomes at delivery, no such modifying effect was evident for low birthweight. The dose–response curves exhibited remarkable similarity across different levels of sulfadoxine–pyrimethamine resistance, both in west and central Africa and in east and southern Africa (figures 2 and 3). For the effect of three versus two doses in west and central Africa, the aRR in areas with very low resistance was 0·58 (95% CI 0·48–0·68), while this was 0·56 (0·44–0·68) in areas with low resistance (p=0·72 for the difference in dose–response curves between very low *vs* low resistance). In east and southern Africa, the aRR for the effect of three versus two doses of sulfadoxine–pyrimethamine on low birthweight were consistently similar across resistance strata: 0·75 (95% CI 0·52–0·98) in moderate resistance areas, 0·73 (0·69–0·78) in high resistance areas, and 0·75 (0·63–0·87) in very high resistance areas (p=0·80 for differences in dose–response curve moderate *vs* high, p=0·90 for moderate *vs* very high, and p=0·87 for high *vs* very high). Comparison of the effects of three versus two doses, four versus three doses, and five versus four doses on low birthweight suggested diminishing incremental benefit with higher doses (appendix pp 27–28).

This absence of a trend towards reduced effectiveness with increased levels of parasite resistance to sulfadoxine–pyrimethamine was also observed for preterm birth. In east and southern Africa, the aRRs for three versus two doses were 0·66 (95% CI 0·22–1·10), 0·65 (0·53–0·77), and 0·54 (0·41–0·68) in areas of moderate, high, and very high resistance, respectively. Similarly, the effect on maternal anaemia remained consistent across resistance strata, with aRRs of 0·95 (95% CI 0·90–1·01), 0·95 (0·93–0·98), and 0·95 (0·92–0·98) for moderate, high, and very high resistance areas in east and southern Africa. The analysis of continuous outcomes yielded comparable results (appendix pp 27–28). The mean differences in birthweight, gestational age, and maternal haemoglobin levels when comparing three versus two doses of sulfadoxine–pyrimethamine showed no clear trend across resistance strata, mirroring the findings observed for the categorical outcomes. Population average estimates (estimates that describe the effect on the entire population, appendix p 29) were similar to the conditional estimates (average effect on the individual participant; [appendix pp 27–28]).

**Figure 4:**
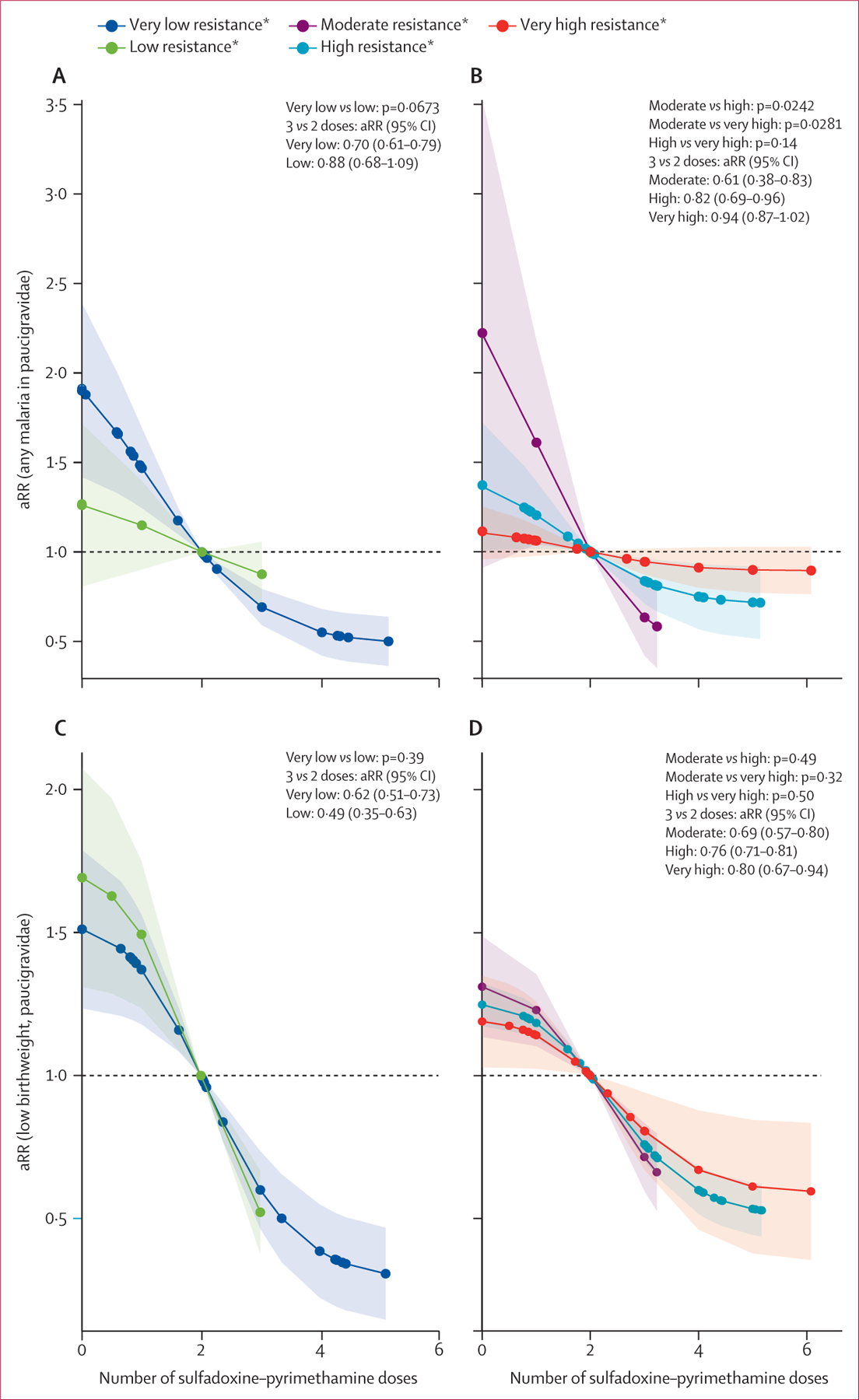
aRRs of malaria infection at delivery and low birthweight associated with IPTp-SP by resistance strata* among paucigravidae, 1993–2021 Adjusted risk ratio for (A) any malaria, west and central Africa, (B) any malaria, east and southern Africa, (C) low birthweight, west and central Africa, and (D) low birthweight, east and southern Africa. p values shown reflect comparisons between dose–response curves across resistance strata, not individual effect estimates of three versus two doses. aRR=adjusted risk ratio. IPTp-SP=intermittent preventive treatment in pregnancy with sulfadoxine–pyrimethamine. *Very low resistance was categorised as Lys540Glu under 4% (dark blue), and low resistance was categorised as Lys540Glu at 4% or above (green). Moderate resistence was categorised as Lys540Glu under 60% and Ala581Gly under 5% (purple), high resistance as Lys540Glu at 60% or above and Ala581Gly under 5% (blue), and very high resistance as Lys540Glu at 60% or above and Ala581Gly at 5% or above (red).

**Figure 5:**
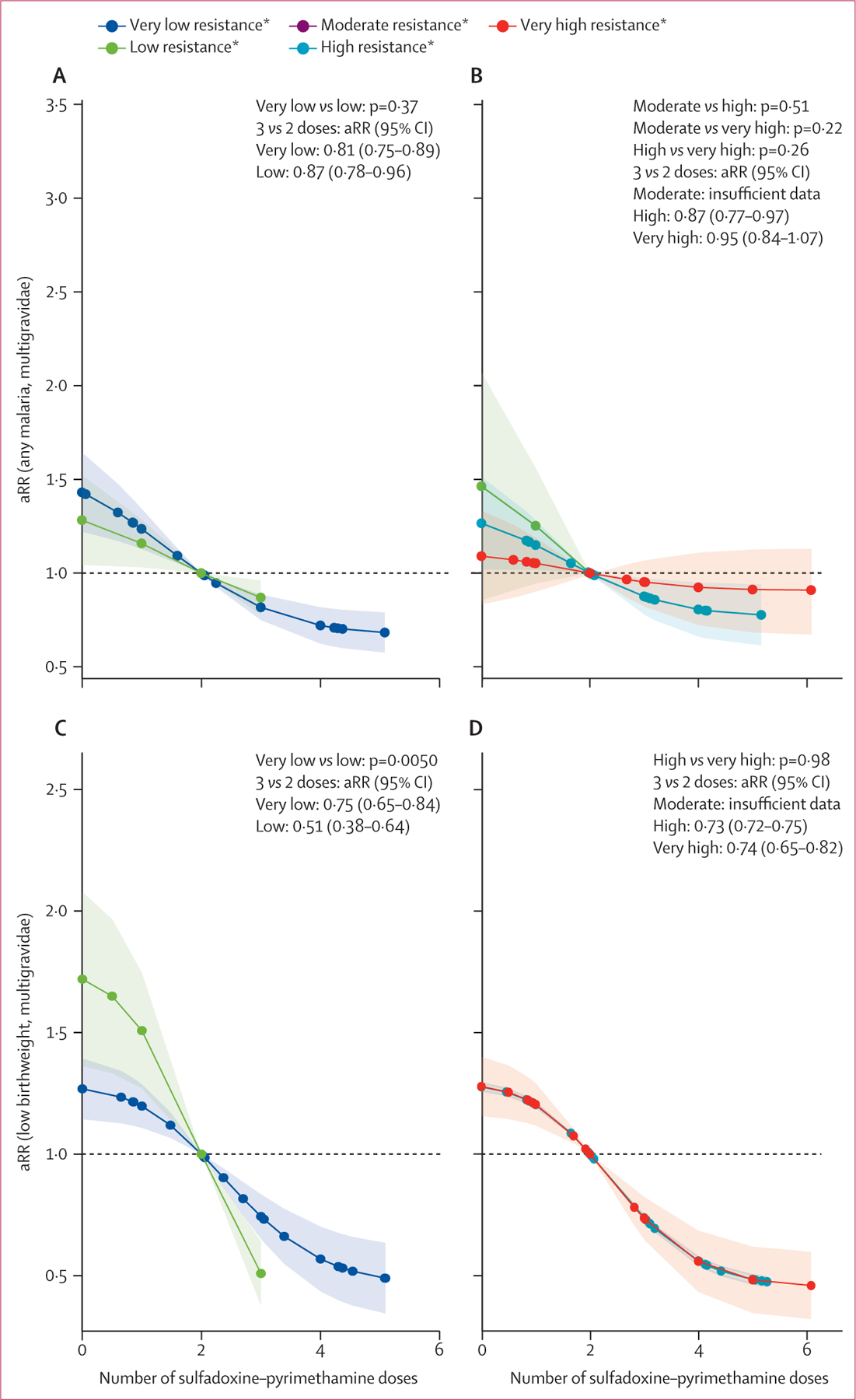
aRRs of malaria infection at delivery and low birthweight associated with IPTp-SP by resistance strata* among multigravidae, 1993–2021 p values shown reflect comparisons between dose–response curves across resistance strata, not individual effect estimates of three versus two doses. *Very low resistance was categorised as Lys540Glu under 4% (dark blue), and low resistance was categorised as Lys540Glu at 4% or above (green). Moderate resistence was categorised as Lys540Glu under 60% and Ala581Gly under 5% (purple), high resistance as Lys540Glu at 60% or above and Ala581Gly under 5% (blue), and very high resistance as Lys540Glu at 60% or above and Ala581Gly at 5% or above (red). aRR=adjusted risk ratio. IPTp-SP=intermittent preventive treatment in pregnancy with sulfadoxine–pyrimethamine. For full details see appendix (p 29).

Patterns for malaria and low birthweight among paucigravidae were similar to those for all gravidae (figure 4). Among multigravidae, differences in the effect on malaria infection across resistance strata were smaller and not statistically significant in both regions. For low birthweight in east and southern Africa, no differences in effects by resistance strata were observed (figure 5). The effectiveness of sulfadoxine–pyrimethamine for low birthweight was higher among multigravidae in the low resistance areas compared to the very low resistance strata in west and central Africa (figure 5). When comparing by dose (appendix p 30), sulfadoxine–pyrimethamine seemed beneficial among multigravidae in very high resistance regions, with a similar aRR as in paucigravidae (eg, four *vs* three doses aRR 0·75 [95% CI 0·67–0·83], for four studies covering 350 women among multigravidae *vs* 0·81 [0·68–0·94], for three studies covering 130 women among paucigravidae; appendix p 30). However, numbers among paucigravidae were low. Adjusting for study quality did not change the results (appendix pp 31–32), and subgroup analyses, including only trials or trials and cohort studies (appendix pp 33–36), gave similar or more protective results.

## Discussion

This analysis builds on our previous meta-analyses.^11,16^ However, unlike the previous analyses, a non-linear relationship between sulfadoxine–pyrimethamine dose and effect was assumed. The results demonstrate a clear trend towards reduced effectiveness of IPTp-SP in controlling malaria infections with increasing resistance to sulfadoxine–pyrimethamine. IPTp-SP had no discernable effect on malaria infections in areas with the highest level of sulfadoxine–pyrimethamine resistance, defined by a prevalence of 5% or more of the *dhps* Ala581Gly mutation in the presence of at least 60% *dhps* Lys540Glu. However, IPTp-SP demonstrated a significant dose-dependent effect on birth outcomes, with each additional course associated with progressive increases in mean birthweight and corresponding reductions in low birthweight, irrespective of resistance level. Similarly, the effect of sulfadoxine–pyrimethamine on anaemia and prematurity did not seem to be affected by sulfadoxine–pyrimethamine resistance strata.

These results confirm that despite a near-complete loss of its antimalarial effects in very high sulfadoxine–pyrimethamine-resistant areas,sulfadoxine–pyrimethamine continues to positively affect fetal growth and the prevention of maternal anaemia. It has been hypothesised that this is mediated through non-antimalarial properties of sulfadoxine–pyrimethamine,^11,17^ consistent with a recent individual participant data meta-analysis of six trials comparing IPTp with dihydroartemisinin–piperaquine versus sulfadoxine–pyrimethamine.^17^ This showed that in high sulfadoxine–pyrimethamine-resistant areas, IPTp with dihydroartemisinin–piperaquine is much more effective in reducing maternal malaria than sulfadoxine–pyrimethamine, preventing about two out of every three infections relative to sulfadoxine–pyrimethamine.^18,19^ However, this did not translate into better pregnancy outcomes, primarily because sulfadoxine–pyrimethamine was associated with better fetal growth, resulting in higher mean birthweights in all gravidae groups, even when these infants were born to mothers with 60% to 70% more malaria infections than in the dihydroartemisinin–piperaquine groups. The results of IPTp trials suggest that sulfadoxine–pyrimethamine’s non-malarial effect could be mediated, at least in part, through better gestational weight gain in the mother.^20,21^ Sulfadoxine is a long-acting sulfonamide with broad-spectrum antimicrobial properties. The associated reduced risk for persistent bacterial infections could influence the maternal gut microbiome, dampen harmful inflammatory responses, or both.^17,22–24^ A Ugandan trial reported reduced occurrence of non-malarial febrile illnesses among pregnant women using IPTp-SP.^25^ A recent in-vitro study, where the adult female intestine was replicated using the human organ-on-a-chip model, suggested a potent effect of sulfadoxine–pyrimethamine on gut health; sulfadoxine–pyrimethamine treatment was noted to reverse multiple intestinal absorptive abnormalities in malnourished female intestinal chip models.^26^ Sulfadoxine–pyrimethamine additionally reduced the production of inflammatory cytokines and suppressed the recruitment of peripheral blood mononuclear cells in chips in nutrient-deficient medium.^26^

A strength of this review is the inclusion of a large number of studies and participants and the use of a novel method to estimate non-linear dose–response relationships, as opposed to the linear dose–response used in the previous meta-analysis. However, there are several limitations. We used the dose–response of IPTp-SP as a proxy marker of efficacy. Observational studies are prone to bias. For example, women receiving a relatively high number of IPTp doses will probably differ from those receiving no or inadequate doses of IPTp-SP. Although multivariable meta-analysis will have reduced the potential for bias, residual confounding is likely to occur because only study-level covariables were available in this aggregated data analysis. For some studies, time and space-matched local sulfadoxine–pyrimethamine resistance data were not available from the source study and had to be obtained from other sources, which were less precise. Additionally, for some comparisons, the sample sizes were low because of lack of studies in specific resistance strata, and these results should be interpreted cautiously as providing directional information rather than definitive outcomes. Lastly, the results might overestimate a sulfadoxine–pyrimethamine effect if studies that reported an effect of sulfadoxine–pyrimethamine doses on outcomes were more likely to be published than those with a negative result. Despite these limitations, the results demonstrate clearly that IPTp-SP continues to result in maternal and fetal benefits despite reductions in antimalarial activity at increasing resistance levels.

In conclusion, this review underscores the dual effects of IPTp with sulfadoxine–pyrimethamine in pregnancy. While sulfadoxine–pyrimethamine’s antimalarial efficacy is severely compromised in high resistance areas, the data demonstrate no evidence for harm and sulfadoxine–pyrimethamine remains notably effective at preventing low birthweight.^27^ This resilient effect may reflect non-malarial mechanisms benefiting fetal growth, which urgently require further elucidation; these effects need to be assessed in malarious and non-malarious regions using trials to repurpose sulfadoxine–pyrimethamine for low birthweight prevention irrespective of the presence of malaria infection.

More effective alternatives are urgently needed to reduce maternal malaria and associated adverse pregnancy outcomes, which may be partially masked by sulfadoxine–pyrimethamine’s malaria-independent effects. However, in the absence of better alternatives that can both control malaria infection and improve birth outcomes, IPTp-SP should continue to be used, even in high sulfadoxine–pyrimethamine-resistance areas. Indeed, sulfadoxine–pyrimethamine confers benefits on fetal growth even where its ability to clear or prevent malaria infections is severely compromised.

This continued use should not diminish the urgency of finding alternative preventive strategies for malaria in pregnancy. The search for alternative drugs, malaria vaccines, or monoclonal antibodies that can either replace or complement sulfadoxine–pyrimethamine remains crucial for comprehensive protection against the impact of malaria on maternal and fetal health. In addition, increased efforts should be made to ensure that all women of reproductive age are using insecticide-treated bednets routinely before and during pregnancy.

## Supplementary Material

1

## Data Availability

Data are in the public domain or de-identified data are available from the WWARN data repository for datasets used. Data from some studies were shared using a data transfer agreement, and are not available through WWARN but at the discretion of the authors of these studies.

## Research in context

### Evidence before this study

We searched the Malaria in Pregnancy Library, Embase (OVID), Global Health (OVID), Cochrane Library, CINAHL (EBSCOHOST), Web of Science (first search only), and Scopus from March 1, 2018, to Dec 4, 2021, and again on June 8, 2024, for cross-sectional surveys, cohort studies or randomised controlled trials, with no language restrictions, using the search terms “Malaria AND pregnan* AND intermittent AND (prevent* OR prophyla* OR chemoprevent* OR chemoprophyla* OR IPT*) AND (sulfadoxine OR sulphadoxine OR pyrimethamine OR SP)”. We included studies reporting on malaria, low birthweight, anaemia, and other outcomes by intermittent preventive treatment during pregnancy with sulfadoxine–pyrimethamine (IPTp-SP) dose, matched with studies reporting on molecular markers of sulfadoxine–pyrimethamine resistance in sub-Saharan Africa, where *Plasmodium falciparum* is the predominant malaria species. Previous studies and meta-analyses have shown that IPTp-SP in pregnancy reduces the risk of malaria parasitaemia, low birthweight, and maternal anaemia. However, concerns have been raised about the effect of increasing parasite resistance to sulfadoxine–pyrimethamine on its effectiveness. A 2019 systematic review and meta-analysis found that IPTp-SP remained associated with reductions in low birthweight even in areas with substantial sulfadoxine–pyrimethamine resistance, albeit with a smaller effect than in areas with less resistance.

### Added value of this study

This updated systematic review and meta-analysis includes additional data from 55 studies and 72 093 pregnancies published since March 1, 2018, and employed a novel methodology to estimate non-linear dose–response relationships. Our findings confirmed a clear relationship between the degree of sulfadoxine–pyrimethamine resistance in the parasite population and the effectiveness of IPTp-SP in reducing malaria infection and confirmed that while the antimalarial effects are severely diminished in areas with the highest levels of resistance, there is no evidence for harm. However, we found that IPTp-SP remains effective at reducing low birthweight, and maternal anaemia, even in areas with high levels of SP resistance. This suggests a potential non-malarial mechanism of sulfadoxine–pyrimethamine that benefits fetal growth and maternal health, which persists despite compromised antimalarial efficacy. Our analysis provides a more nuanced understanding of the dual effects of sulfadoxine–pyrimethamine, highlighting its continued value in improving birth outcomes even as its ability to prevent malaria infection decreases.

### Implications of all the available evidence

Our findings have important implications for malaria control programs and policy makers. While the search for more effective antimalarials to reduce maternal malaria and associated adverse pregnancy outcomes should remain a priority, IPTp-SP should continue to be used even in areas with high sulfadoxine–pyrimethamine resistance. The persistent benefits of sulfadoxine–pyrimethamine on fetal growth and maternal health, independent of its antimalarial effects, justify its continued use in the absence of better alternatives that can both control malaria infection and improve birth outcomes. However, this should not diminish the urgency of developing new preventive strategies for malaria in pregnancy. Future research should focus on elucidating the non-malarial mechanisms by which SP improves birth outcomes and on studies evaluating alternative antimalarials, malaria vaccines or monoclonal antibodies that can either replace or complement SP to provide comprehensive protection against malaria’s impacts on maternal and fetal health. Additionally, continued monitoring of SP resistance and its impact on various pregnancy outcomes is crucial to inform timely policy decisions regarding malaria prevention strategies in pregnancy.
