# Supplementary material for: The impact of sulfadoxine–pyrimethamine resistance on the effectiveness of intermittent preventive treatment for the prevention of malaria in pregnancy in Africa: an updated systematic review and meta-analysis
Source: Lancet Infect Dis. Author manuscript; Available in PMC 2025 Dec 27. (PMC12743599; doi:10.1016/S1473-3099(25)00219-1)
Supplement: 1 [file NIHMS2126177-supplement-1.pdf]

THE LANCET
Infectious Diseases

Supplementary appendix

This appendix formed part of the original submission and has been peer reviewed. We post it as supplied by the authors.

Supplement to: van Eijk AM, Stepniewska K, Khairallah C, et al. The impact of sulfadoxine–pyrimethamine resistance on the effectiveness of intermittent preventive treatment for the prevention of malaria in pregnancy in Africa: an updated systematic review and meta-analysis. *Lancet Infect Dis* 2025; published online July 14. https://doi.org/10.1016/S1473-3099(25)00219-1.

SUPPLEMENTARY APPENDIX

Supplement to:

The impact of sulfadoxine-pyrimethamine resistance on the effectiveness of intermittent preventive treatment for the prevention of malaria in pregnancy: an updated systematic review and meta-analysis

Authors

Anna Maria van Eijk, Kasia Stepniewska, Carole Khairallah, Eva Rodriguez, Jordan Ahn, Manfred Accrombessi, Yaa Nyarko Agyeman, Professor Eleni Aklillu, Emmanual Arinaitwe, Paulo Arnaldo, Gideon Darko Asamoah, Professor Per Ashorn, Professor James A. Berkley, Valerie Briand, Enesia Banda Chapondo, R. Matthew Chico, Jobiba Chinkhumba, Lauren Cohee, Professor Sheick Oumar Coulibaly, Professor Umberto d'Alessandro, Meghna Desai, Professor Alassane Dicko, Professor Grant Dorsey, Patrick Duffy, Professor Gaoqian Feng, Professor Jennifer Flegg, Michal Fried, Professor Brian Greenwood, Mary Hamel, Aurore Hounto, Japhet Kabalu-Tshiongo, Richard Kajubi, Abel Kakuru, Linda Kalilani-Phiri, Alice Kamau, Professor Kassoum Kayentao, Professor Christopher L. King, Professor Miriam Laufer, Ruth Lemwayi, Moussa Lingani, Mari Luntamo, Kimberly E. Mace, Mwayiwawo Madanitsa, Almahamoudou Mahamar, Indu Malhotra, Professor Junior Matangila-Rika, Professor Don. P. Mathanga, Petra Mens, Professor Omary Minzi, Eulambius Mathias Mlugu, Moses M. Musau, Sadiatu S. Obi, Peter Ouma, Professor Ellis Owusu-Dabo, Professor Stephen J. Rogerson, Professor Anna Rosanas-Urgell, Henk Schallig, Susana Scott, Professor Robert W. Snow, Professor Beverly I. Strassmann, Julie Niemczura Sutton, Professor Harry Tagbor, Steve M. Taylor, Professor Halidou Tinto, Almamy Amara Toure, Claudius Vincenz, Professor Trésor Zola-Matuvanga, Julie R. Gutman, and Professor Feiko O. ter Kuile.

Affiliations

Department of Clinical Sciences, Liverpool School of Tropical Medicine, Liverpool, UK (AM van Eijk PhD, C Khairallah MsC, L Cohee MD, A Kamau PhD, Professor F O ter Kuile PhD); Infectious Diseases Data Observatory (IDDO), University of Oxford, Oxford, UK (K Stepnieuwska PhD); Emory University, Atlanta, GA, USA (E Rodriguez MSc, J Ahn MSc); Malaria Branch, Division of Parasitic Diseases and Malaria, Center for Global Health, Centers for Disease Control and Prevention (CDC), Atlanta, GA, USA (J Gutman MD, K E Mace PhD); London School of Hygiene & Tropical Medicine, UK (M Accrombessi, R M Chico, Professor B Greenwood MD, Professor U d'Alessandro PhD, S Scott PhD); Institut de Recherche Clinique du Benin (IRCB), Abomey-Calavi, Benin (M Accrombessi PhD); School of Public Health, University for Development Studies, Tamale, Northern Region, Ghana (Y N Agyeman PhD); Department of Global Public Health, Karolinska Institutet, Karolinska University Hospital, Stockholm Sweden (Professor E Akililu PhD); Infectious Diseases Research Collaboration (IDRC), Kampala, Uganda (E Arinaitwe PhD, R Kajubi MSc, A Kakuru PhD); Instituto Nacional de Saúde, Maputo, Mozambique (P Arnaldo PhD); MRC Institute of Clinical Trials and Methodology, University College London, London, UK (G D Asamoah MSc); Center for Child, Adolescent and Maternal Health Research, Faculty of Medicine and Health Technology, Tampere University, Tampere, Finland (Professor P Ashorn PhD, M Luntamo MD); Department of Paediatrics, Tampere University Hospital, Tampere, Finland (Professor P Ashorn PhD); Kenya Medical Research Institute–Wellcome Trust Research Programme, Nairobi, Kenya (Professor J A Berkley MD, A Kamau PhD, M M Musau MSc, Professor R W Snow FMedSci); Centre for Clinical Vaccinology and Tropical Medicine, Churchill Hospital, University of Oxford, Oxford, United Kingdom (Professor J A Berkley MD); IRD, Inserm, Université de Bordeaux, IDLIC team, UMR 1219, Bordeaux, France (V Briand MD); Department of Biological Sciences, University of Zambia, Lusaka, Zambia (E B Chapondo PhD);

Malaria Alert Center, Malawi College of Medicine, Blantyre, Malawi (J Chinkhumba PhD); Unité de formation et de recherche en Sciences de la Santé, Université Joseph Ki-Zerbo de Ouagadougou, Burkina Faso (Professor S O Coulibaly PhD); U.S. Centers for Disease Control and Prevention, New Delhi, India (M Desai PhD); Malaria Research & Training Center, Faculty of Medicine, Pharmacy and Dentistry, University of Sciences Techniques and Technologies of Bamako, Bamako, Mali (Professor A Dicko MD, Professor K Kayentao PhD, A Mahamar PhD); University of California San Francisco, USA (Professor G Dorsey MD); Laboratory of Malaria Immunology and Vaccinology, National Institute of Allergy and Infectious Diseases, Bethesda, United States (P Duffy MD); Burnet Institute, Melbourne, VIC, Australia; Department of Medicine, The University of Melbourne, Melbourne, VIC, Australia (Professor G Feng PhD); School of Mathematics and Statistics, The University of Melbourne, Parkville, Australia (Professor J A Flegg PhD); Laboratory of Malaria Immunology and Vaccinology, National Institute of Allergy and Infectious Diseases, Rockville, United States (M Fried PhD); Department of Immunizations, Vaccines, and Biologicals, WHO, Geneva, Switzerland (M Hamel MD); Unité de Parasitologie/Faculté des Sciences de la Santé, Université d'Abomey Calavi, Cotonou, Benin (A Hounto MD); Department of Tropical Medicine, University of Kinshasa (UNIKIN), Kinshasa, Democratic Republic of the Congo (J Kabalu-Tshiongo MD, J Matangila-Rika PhD, Professor T Zola-Matuvanga PhD); Department of Community and Environmental Health, Kamuzu University of Health Sciences, Blantyre, Malawi (L Kalilani-Phiri PhD); School of Pharmacy, Muhimbili University of Health and Allied Sciences, Dar Es Salaam, Tanzania (Professor O Minzi PhD, E M Mlugu PhD); Center for Global Health and Diseases, Department of Pathology, Case Western Reserve University School of Medicine, Cleveland, OH USA (Professor C. King PhD, I Malhotra PhD); Center for Vaccine Development and Global Health, University of Maryland School of Medicine, Baltimore, MD USA (Professor M Laufer MD, L Cohee MD); USAID Afya Yangu – Mama na Mtoto, Mwanza, Tanzania (R Lemwayi MD); Institut de Recherche en Sciences de la Santé/Direction Régionale du Centre Ouest (IRSS/DRCO), Nanoro, Burkina Faso (M Lingani MD, Professor H Tinto PhD); Academy of Medical Sciences, Malawi University of Science and Technology, Malawi (m Madanitsa PhD); Department of Public Health, Kamuzu University of Health Sciences, Blantyre, Malawi (Professor D P Mathanga PhD); Amsterdam University Medical Centers, Academic Medical Center at the University of Amsterdam, Department of Medical Microbiology & Infection Prevention, Lab Experimental Parasitology, Amsterdam, The Netherlands (P Mens PhD, H Schallig PhD); Nigerian Institute of Medical Research, outstation Asaba, Delta State Nigeria (S S Obi PhD); Kenya Medical Research Institute (KEMRI)/Centre for Global Health Research, Kisumu, Kenya (P Ouma PhD); Department of Global and International Health, School of Public Health, Kwame Nkrumah University of Science and Technology, Ghana (Professor E Owusu-Dabo MD); Department of Infectious Diseases, The University of Melbourne, The Doherty Institute, Melbourne Victoria, Australia (Professor Stephen Rogerson PhD); Department of Biomedical Sciences, Institute of Tropical Medicine Antwerp, Belgium (Professor A Rosanas-Urgell PhD); Centre for Tropical Medicine and Global Health, Nuffield Department of Clinical Medicine, University of Oxford, Oxford, UK (Professor R W Snow FMedSci); Department of Anthropology, University of Michigan, Ann Arbor, Michigan, USA (Professor B I Strassman PhD); PMI Impact Malaria/MCD Global Health, Silver Spring, MD, USA (J N Sutton MPH); University of Health and Allied Sciences, Ho, Ghana (Professor H Tagbor DrPH); Division of Infectious Diseases and Duke Global Health Institute, Duke University, Durham, NC, USA (S M Taylor MD); Centre National de Formation et de Recherche en Santé Rurale de Maferinyah. République de Guinée (A A Toure MD); Research Center for Group Dynamics, Institute for Social Research, University of Michigan, Ann Arbor, Michigan, USA (C Vincenz PhD).

Content of supplementary appendix

Supplemental Methods 5
PICOS Table 5
Search strategy 5
Eligibility criteria 7
Definitions of maternal outcomes 7
Data extraction and quality assessment observational studies and trials 7
Quality assessment form for observational studies and trials 8
Matching of clinical studies with population-level data from other sources 9
Malaria transmission 9
SP resistance markers 9
HIV prevalence and bednet use 10
Statistical methods 10
Defining thresholds for resistance based on malaria infection as delivery 10
Transformation of SP dose variable and non-linear modelling 11
Deviations from the study protocol 12
Supplemental Results 12
Gestational age assessment in participating studies 12
Supplemental Tables 13
Table S1. Study characteristics of studies with information on outcomes by SP doses 13
Table S2. Matching of studies with information on SP resistance markers (*Pfdhps*-A437G, *Pfdhps*-K540E and *Pfdhps*-A581G) 20
Table S3: Adjusted risk ratio and mean difference for several outcomes associated with a difference in IPTp-SP dose by resistance strata in each region, sub-Saharan Africa, 1993-2021 27
Table S4. Population average adjusted risk ratio and mean difference for several outcomes associated with a difference in IPTp-SP dose by resistance strata in each region, 1997-2021 29
Table S5. Adjusted risk ratio for any malaria and low birth weight associated with a difference in IPTp-SP dose by resistance strata in each region by gravidity, 1997-2021 30
Table S6: Adjusted risk ratio and mean difference for outcomes associated with a difference in IPTp-SP dose by resistance strata in each region, when including study-quality-assessment as co-variate in the model, sub-Saharan Africa, 1997-2021 31
Table S7: Adjusted risk ratio and mean difference for several outcomes associated with a difference in IPTp-SP dose by resistance strata in each region, sub-Saharan Africa, 1997-2021, trials only 33
Table S8: Adjusted risk ratio and mean difference for several outcomes associated with a difference in IPTp-SP dose by resistance strata in each region, sub-Saharan Africa, 1997-2021, trials and cohort studies only 35
Table S9. AICs for the association between SP doses and pregnancy outcomes 37

Table S9b: Transformation of SP dose variable explored 39
Supplemental Figures 40
Figure S1. Map of sites included in the analyses 40
Figure S2. Relationship between the prevalence of the *dhps* Ala437Gly and *dhps* Lys540EGlu mutation in the study locations in central and west Africa and east and southern Africa 41
Figure S3. Transformation used for analyses and transformations with the highest AICs for each outcome 42
Supplemental References 44

Supplemental Methods

PICOS Table

**Population**	Pregnant women living in malaria-endemic settings
**Background**	Standard of care at the time the study was conducted
**Intervention**	Administration of full therapeutic courses of sulfadoxine-pyrimethamine (SP) at predetermined times for intermittent preventive treatment in pregnancy (IPTp)
**Comparison**	No IPTp group (0 doses of IPTp) or inadequate IPTp doses, defined as 0 or 1 SP dose for the comparison with ≥2 doses or ≤2 doses for the comparison with ≥3 doses
**Outcomes**	*In the mother:*<br>Anaemia/ haemoglobin<br>Placental malaria infection<br>Preterm delivery<br>Maternal parasite prevalence<br>*In the foetus and infant:*<br>Low birth weight/birthweight
**Timeframe**	Third trimester and at birth
**Timing**	No time restriction
**Potential effect modifiers**	Transmission intensity<br>Gravidity (stratified by 1-2 and ≥3 where possible, and otherwise as defined in the source study)<br>Prevalence of anti-folate (SP) drug resistance<br>Seasonality<br>Vector control coverage (insecticide-treated net [ITNs] use, Indoor residual spraying)
**Additional considerations**	Coverage of antenatal care (proportion ≥1 antenatal visit as reported by study)<br>Plasmodium species<br>HIV<br>Timing of the first dose<br>Maternal age<br>First line treatment of malaria

Search strategy

This review (Prospero CRD42021250359) is an update of a previous evaluation of the impact of *Plasmodium falciparum* resistance to sulfadoxine-pyrimethamine (SP) on the effectiveness of intermittent preventive treatment in pregnancy (IPTp) with SP (Prospero CRD42016035540).[1] The following search terms were used for the aggregated data meta-analysis: “Malaria AND pregnan* AND intermittent AND (prevent* OR prophyla* OR chemoprevent* OR chemoprophyla* OR IPT*) AND (sulfadoxine OR sulphadoxine OR pyrimethamine OR SP)”. The electronic databases “Malaria in Pregnancy Library” (https://mip.wwarn.org),[2] PubMed/MEDLINE (OVID), Embase (OVID), Global Health (OVID), Cochrane Library, CINAHL (EBSCOHOST), Web of Science (first search only), and Scopus were searched from March 2018 to December 4, 2021, and updated June 8, 2024. In

addition, we screened reference lists and consulted experts in the field. In the previous review, included studies had to have a zero dose or inadequate dose SP group (0-1 doses) as the comparator group and a 2 or more dose group as intervention group and the search was conducted from January 1, 1990 to March 1, 2018.[1] Given the current recommendation of 3+ doses of SP and the different methodology used in this review, the inclusion criteria were widened to studies with any number of doses (≥2 dose-groups), as long as the outcomes were reported by number of SP doses. Studies already included in the previous publication were screened on outcomes of interest or SP dose groupings that were not included in the previous review. This search was conducted for the time period of March 1, 2018, to June 8, 2024.

Additionally, we ran a search for clinical trials using the same sources and search terms for articles published prior to March 2018, but with inclusion of the terms "AND trial* OR RCT* OR clinical stud*" to ensure that all older studies comparing two versus three or more doses of SP were included.

All searches were conducted in English but without language restriction.

**Database**	**Search 01 March 2018 – 08 June 2024: Strategy**	**Records**
**PubMed/Medline (OVID)**	Malaria AND pregnan* AND intermittent AND (prevent* OR prophyla* OR chemoprevent* OR chemoprophyla* OR IPT*) AND (sulfadoxine OR sulphadoxine OR pyrimethamine OR SP	268
**Embase (OVID)**	Malaria AND pregnan* AND intermittent AND (prevent* OR prophyla* OR chemoprevent* OR chemoprophyla* OR IPT*) AND (sulfadoxine OR sulphadoxine OR pyrimethamine OR SP)	363
**Global Health (OVID)**	Malaria AND pregnan* AND intermittent AND (prevent* OR prophyla* OR chemoprevent* OR chemoprophyla* OR IPT*) AND (sulfadoxine OR sulphadoxine OR pyrimethamine OR SP)	214
**Cochrane Library**	(Malaria AND pregnan* AND intermittent AND (prevent* OR prophyla* OR chemoprevent* OR chemoprophyla* OR IPT*) AND (sulfadoxine OR sulphadoxine OR pyrimethamine OR SP)):ti,ab	110
**CINAHL (EbscoHost)**	Malaria AND pregnan* AND intermittent AND (prevent* OR prophyla* OR chemoprevent* OR chemoprophyla* OR IPT*) AND (sulfadoxine OR sulphadoxine OR pyrimethamine OR SP)	67
**Scopus**	TITLE-ABS-KEY(Malaria AND pregnan* AND intermittent AND (prevent* OR prophyla* OR chemoprevent* OR chemoprophyla* OR IPT*) AND (sulfadoxine OR sulphadoxine OR pyrimethamine OR SP))	326
**Malaria in pregnancy library**	(Intermittent AND (prevent* OR prophyla* OR chemoprevent* OR chemoprophyla* OR IPT*) AND (sulfadoxine OR sulphadoxine OR pyrimethamine OR SP)):ti,ab	628

Eligibility criteria

Observational studies were included if they were conducted in sub-Saharan Africa, had information at delivery on the number of SP doses received, and data on peripheral or placental plasmodium infection, low birthweight or mean birthweight, anaemia (haemoglobin <11 g/dL) or mean maternal haemoglobin at delivery, or any other outcome of interest. Trials were included if they were quasi-randomized or randomized trials; were conducted in sub-Saharan Africa; compared IPTp-SP against passive case detection or placebo or otherwise fulfilled the same criteria as for the observational studies. Studies or study arms were excluded if they involved only HIV-infected women, combined SP with other antimalarial drugs, such as artemisinin derivatives or azithromycin, or other interventions, such as screening for malaria. Two reviewers independently screened titles and abstracts identified from literature searches. The same two reviewers evaluated any potentially relevant articles identified by at least one reviewer for full-text eligibility based on pre-determined inclusion criteria. Disagreements were resolved by consensus. Multiple publications from the same study were grouped and included once as a single study at the full-text stage.

Definitions of maternal outcomes

- Maternal anaemia: Any anaemia at delivery or in the 3rd trimester: haematocrit <33% or haemoglobin <10 or 11 g/dL as reported by the source study
- Malaria at delivery: Peripheral or placental malaria infection detected by microscopy, malaria rapid diagnostic test (RDT), polymerase chain reaction (PCR), or histopathology in placental or peripheral maternal blood, regardless of the presence of fever or history of fever
- Peripheral malaria at delivery: Maternal peripheral blood malaria measured at delivery by microscopy, malaria RDT or PCR, regardless of the presence of fever/ history of fever
- Placental malaria at delivery: Placental malaria detected by any diagnostic test
- Adverse pregnancy outcome:
    - Preterm delivery: delivery <37 weeks gestational age
    - Low birth weight: birth weight <2,500 gram
- Continuous variables of pregnancy outcome:
    - Gestational age at delivery
    - Newborn birthweight
    - Maternal haemoglobin (delivery or third trimester)

Data extraction and quality assessment observational studies and trials

Two reviewers independently extracted information from studies on a pre-piloted electronic data extraction form. Any discrepancies in information were resolved by consensus or consultation with a third reviewer. We contacted the study author(s) to retrieve any missing data or for clarification. Authors of potentially eligible studies with insufficient information for extraction (or with potentially more information than was extractable) were approached by email and sent one reminder. If there was no response, the study was excluded. Data on first author, publication year, study period (year of start and end of study), study design, inclusion criteria, study settings (country, location), population characteristics (gravidity, age, setting, season), withdrawal and loss to follow-up, details of the intervention, ITN use, HIV-infection, antenatal care visits, and outcome data were extracted. If available, SP resistance data was extracted. For binary outcomes (Yes/no), we extracted the number of participants with an event (n) and the number of participants (N) per SP dose group. For continuous data, we extracted the mean and a measure of variance (e.g., standard deviation) and the number of participants contributing per SP dose group. For the clinical trials, only participants in

the IPTp-SP arms were included for the efficacy analysis of the effect of SP by the number of SP doses received. For studies where the time of conduct of the study was not reported or could not be obtained, the study was assumed to have been conducted two years before the publication date,[3,4] based on the analysis of the Malaria in Pregnancy library content.[5] Data from studies with multiple publications were combined into a single entry to avoid duplication. If individual participant data was available, this data was used to prepare the tables needed for aggregated data analysis (outcomes of interest by number of SP-doses). No individual participant data analysis was conducted.

Because data from trials and observational studies were both treated as cohort studies with the number of SP doses as exposure and low birthweight (or other parameters) as the measured outcomes, the same quality assessment form (an adaptation of the Newcastle-Ottawa Scale for cohort studies) was used for both designs. We modified the Newcastle-Ottawa Scale in two key aspects: (1) we expanded the "ascertainment of exposure" category to include antenatal care (ANC) records as the data source option, and (2) we revised the "comparability of cohorts" assessment to specifically evaluate whether differences in participant characteristics across SP dose groups were examined and reported. To maintain consistency with our previous analyses, we did not incorporate two items that were added in more recent versions of the scale ("Demonstration that outcome of interest was not present at the start of the study" and "Was follow-up long enough for outcome to occur"). For our adaptation, studies could achieve a maximum quality score of 6 points, with scores of 5 or 6 considered good quality and scores below 5 considered moderate-to-low quality.

Two reviewers conducted the quality assessment; where a disagreement occurred, a joint review of the specific study was conducted to reach consensus. Follow-up or outcome was considered adequate if more than 80% of participants initially enrolled were included in the analysis. Studies were not a-priori excluded based on their quality score. The study quality score was used as a potential confounder or effect modifier in the analyses.

Quality assessment form for observational studies and trials

	Focus area	Category options†
1	Representativeness of the exposed group	a) truly representative of pregnant women in the community (e.g. random selection in community) *<br>b) somewhat representative of the average pregnant woman in the community (e.g. selection in ANC) *<br>c) selected group of pregnant women (e.g. women who deliver in a health unit)<br>d) no description of the derivation of the group
2	Selection of the non-exposed group	a) drawn from the same community/pool as the exposed group *<br>b) drawn from a different source<br>c) no description of the derivation of the non-exposed group
3	Ascertainment of exposure	a) ANC record (e.g. antenatal clinic notes)<br>b) structured interview<br>c) combination of ANC notes and interview *<br>d) observed and prospectively collected (trial or cohort study) *<br>e) unsecure record<br>f) written self-report<br>g) no description
4	Comparability of exposed and unexposed group	a) differences examined and no differences reported in characteristics which are presented *<br>b) differences in characteristics present but no effect on outcome, or multivariable analysis for outcome available or randomized study *<br>c) differences in characteristic present, not shown if effect on outcome<br>d) no description/not examined

5	Outcome assessment (low birthweight, haemoglobin, malaria)	a) independent blind assessment *<br>b) record linkage *<br>c) not clear<br>d) no blind assessment<br>e) no description
6	Attrition	a) complete - all subjects accounted for *<br>b) Outcome not available for all subjects but unlikely to introduce bias - small number lost - <20%, or description provided of those lost *<br>c) Outcome for less than 80% of people with exposure data and no description of those lost<br>d) no statement

† A study could be awarded a maximum of one star for each item

Matching of clinical studies with population-level data from other sources

Information on the latitude and longitude of study locations was obtained for all included studies using Google Earth. For each study, we calculated the midyear, which represents the calendar year that falls at the midpoint of the study's duration (e.g., for a study conducted from 2011 to 2013 inclusive, the midyear would be 2012). For studies with no information on study years, we assumed the study was conducted two years before the publication unless the authors provided this information.[5] These geographical coordinates and midyear dates were essential for matching the clinical studies with various population-level data sources, including malaria transmission intensity from the Malaria Atlas Project, molecular markers of SP resistance, HIV prevalence data, and insecticide-treated net (ITN) use estimates as outlined below.

Malaria transmission

Using the geographical location and midyear, we assigned each study an indicator of malaria transmission at the time of the study using the *P. falciparum* prevalence among children aged 2-10 years (*Pf*$PR_{2\text{-}10}$) from the Malaria Atlas Project (https://malariaatlas.org/explorer/#/). The studies included in the 2019 review were reassigned a *Plasmodium falciparum* $PR_{2\text{ - }10}$ value using version 2020 of the Malaria Atlas Project for consistency.[1]

SP resistance markers

Data on the prevalence of *dhps* Ala437Gly, Lys540Glu, and Ala581Gly mutations among *P. falciparum* parasites were extracted from the clinical studies in pregnant women (if provided), and otherwise, the literature or existing molecular surveyor databases were used (https://www.wwarn.org/tracking-resistance/sp-molecular-surveyor). For studies without a good match, the prevalence was obtained from models as described by Flegg *et al*.[6,7]

Studies were categorised by geographic region into two groups based on established parasite population divisions: central and west Africa (Benin, Burkina Faso, Cameroon, Cote d'Ivoire, [western] Democratic Republic of Congo [DRC], Gabon, Ghana, Guinea, Mali, Nigeria, Republic of Congo, Senegal, The Gambia) and east and southern Africa ([eastern] DRC, Kenya, Malawi, Mozambique, Sudan, Tanzania, Uganda, Zambia, Zimbabwe). Previous research has established distinct parasite populations with different distributions of resistance mutations between these regions, with the division approximately following a line through the Democratic Republic of Congo. Consequently, studies conducted in the Democratic Republic of Congo were assigned to either "central and west Africa" or "east and southern Africa" based on their specific geographical location within the country. One study with multiple sites spanning both eastern and western DRC was split,

with sites in western DRC allocated to "central and west Africa" and sites in eastern DRC allocated to "east and southern Africa", as detailed in Tables S1 and S2.

We attempted to match the prevalence of each point mutation for SP resistance markers to each study as in the previous study by time (within 3 years before or after for point mutations) and by location (within 300 km).[8] A score was assigned to each match, based on the time between the study on SP resistance marker and the clinical study, the distance between the location of SP resistance marker assessment and the clinical study and the overall sample size (≥30 yes/no) for SP resistance marker, with a maximum score of three points. A good match was defined as a study conducted within 3 years and within 300 km of the data of interest, and a sample size >=30 for the assessment of the marker; these studies would receive 3 points. A point was not given if a criterion resistance was not met. Optimal matching was defined as a maximum score of three for matching for *dhps* Lys540Glu for a study in west and central Africa and for *dhps* Lys540Glu and AlaA581Gly for a study in east and southern Africa. Non-optimal matches were identified but retained in the analyses. Of the 84 locations in west and central Africa, only five (6.0%) had a sub-optimal match (score of 2) for dhps Lys540Glu, and none had a score of 1. Similarly, among 59 locations in east and southern Africa, only one (1.7%) had a suboptimal match (score of 2) for both dhps Lys540Glu and AlaA581Gly markers (Table S2).

HIV prevalence and bednet use

To explore the effect of HIV, we added the country-level HIV prevalence corresponding to each study's midyear, obtained from UNAIDS, for studies lacking study-level or participant-level HIV information. Similarly, for studies without data on ITN use, we incorporated ITN coverage estimates specific to each study's geographical location and midyear, sourced from the Malaria Atlas Project (https://malariaatlas.org/).

Statistical methods

All analyses were conducted in Stata (StataCorp, College Station, TX, USA, version 17).

Defining thresholds for resistance based on malaria infection as delivery

To define thresholds for resistance, we used the outcome of "any malaria at delivery", defined as placental or maternal malaria detected by any diagnostic test at delivery. In areas where the prevalence of *dhps* Lys540Glu was more than 50%, the prevalence of the *dhps* Ala581Gly mutation served as a proxy for the sextuple mutant. Threshold analyses were done separately for central and west Africa and east and southern Africa because of distinct parasite populations and distributions of mutations in each region (p41: Figure S2).[9,10] We considered the following cutoffs:

1) West and central Africa: *dhps* Ala437Gly starting from 20%, increasing with steps of 5% until 100%; Lys540Glu starting at 1%, increasing with steps of 1% until 5%, and continuing increasing with steps of 5% until 35%.
2) East and southern Africa: *dhps* Lys540Glu starting from 20%, increasing with steps of 5% until 100%; AlaA581Gly at the levels 0·2, 1, 2, 3, 4, 5, 10, 15 and 16%.

We explored all possible options with 2, 3, and 4 categories for resistance using all combinations of these cutoffs for each region. For each cutoff, or combination of cutoffs and the corresponding definition of the resistance variable, Poisson mixed effects model were fitted to the number of malaria cases, with the total number of participants taken as an exposure variable. Study-site was a cluster variable, and random intercept and slope for SP dose were included in the model since they consistently gave lower mean Akaike information criterium than the corresponding models with random intercept only. Dose was modelled after the approximate cumulative distribution (ACD)

transformation to allow for the S-shape dose-response relationship.[11] This ACD transformation enables modelling a sigmoid relationship between a continuous covariate X and an outcome variable. Sigmoid relationships exhibit asymptotes at one or both ends of the continuous covariate (X) range (representing minimum and maximum effect), which cannot be accurately represented by fractional polynomials. Computational details of the ACD approach are presented by Royston (2014).

In a model where there is a linear relationship between Y and ACD(X): E(Y)=β0 +β1ACD(X), the relationship between Y and X is nonlinear and typically sigmoid in shape (and can be shown graphically). The parameters β0 and β0 +β1 in such a model are interpreted as the expected values of Y at the minimum and maximum of X, respectively (at ACD(X) = 0 and 1). The parameter β1 represents the range of predictions of E(Y) across the entire observed distribution of X

This approach is particularly suitable for modelling antimalarial dose-response relationships, where efficacy often follows a sigmoid pattern with diminishing returns at higher doses as response approaches maximal effect.

Other nonlinear relationships were explored using fractional polynomials, but ACD transformation provided the best fit to the data. Interactions between the transformed dose variable and the resistance variable categories were included in the model, so that the separate dose-response relationship was fitted for each of the resistance categories. The resistance variable was included in the model only through the interaction terms (no main effects) as it was assumed that the only association between the resistance variable and the outcome (number of cases) was via the possibly altered efficacy of the treatment (changed dose-effect relationship). All models were adjusted for malaria transmission intensity (*Pf*$PR_{2\text{-}10}$), estimated HIV infection prevalence, percentage of paucigravidae in the study, and ITN use.[1]

The performance of models was evaluated using cross-validation. Each study was excluded in turn, and the model was fitted to the data from the remaining studies, and the prevalence of malaria cases was predicted for the excluded study. The mean value of Akaike information criteria and rootmean square error were used to assess the performance.

Root Mean Square Error (RMSE):

$$\text{RSME} = \sqrt{\sum_{i=1}^{N} \frac{1}{N} (y_i - \hat{y}_i)^2}$$

where y_i is the observed malaria prevalence, $\hat{y}_i$ is the predicted malaria prevalence from the model that excluded this study-site, and N is the total number of data points.

For central and west Africa, very low resistance was categorized as Lys540Glu<4% and low if Lys540Glu≥4%; for east and southern Africa moderate resistance was categorized as Lys540Glu<60 & Ala581Gly<5%, high as Lys540Glu≥60 & Ala581Gly<5% and very high as Lys540Glu≥60 & Ala581Gly≥5%.

Transformation of SP dose variable and non-linear modelling

For each outcome, we explored nonlinear transformations of SP dose and resistance level using fractional polynomials in Stata and ranked the Akaike information criteria for each model (supplement table S9, page 37). For additional malaria outcomes (peripheral and placental malaria), the same transformation was used as for “any malaria”, and the second-best models are shown in the supplement as sensitivity analysis (Figure S3, page 42). We ignored linear models because we did not think these would be plausible. For birthweight, low birth weight, preterm delivery and gestational age, results were ranked, and the transformation with the lowest score when combining

all four outcomes was used in further analyses (this was the ACD transformation followed by a quadratic transformation, T14). The same procedure was followed for haemoglobin and anaemia (root transformation, T5, was used). The second-best models are shown in the supplement as part of the sensitivity analysis (Figures S3, page 42). To assess statistical differences between the non-linear effects of interaction terms representing SP dose and resistance category in our mixed-effects Poisson regression model, we employed a Wald test. This approach allows for simultaneous comparison of multiple coefficients while accommodating the heterogeneity in study designs and incorporating robust standard errors in our analysis. Specifically, we tested the null hypothesis that the coefficients for these interaction terms were equal (H0: $\beta 1 = \beta 2 = \beta 3$), which would indicate no significant difference in their effects. The test was performed post-estimation using Stata's 'test' command. A p-value < 0·05 was considered statistically significant, indicating heterogeneity in the effects of these variables. To further elucidate specific differences, we conducted pairwise comparisons between the interaction terms.

For each outcome (any malaria, peripheral and placental malaria, low birth weight, preterm delivery, anaemia, birth weight, gestational age and haemoglobin) graphs were prepared by region, using two doses of SP as the reference group, showing the adjusted risk ratio or adjusted mean difference by different resistance strata in each region. Additionally, the adjusted risk ratio or mean difference was calculated for 3 doses compared to 2, 4 doses compared to 3 and 5 doses compared to 4, by region and resistance strata when there was sufficient data, using the same models. For comparison, we also calculated marginal effects: these are the effects for a population rather than for an individual, using the margins command in Stata (table S4, page 29).

Deviations from the study protocol

Although we initially planned a generalised least square regression for trend estimation of summarized dose-response data and the use of meta-regression to assess the effect of SP-resistance, this was replaced with non-linear mixed-effects Poisson models, as this was thought to better align with biological relationships than linear models.

Supplemental Results

Gestational age assessment in participating studies

We checked how gestational age was measured in the different studies. Fourteen studies reported the use of ultrasound (11.5%), the use of last menstrual period (LMP) was reported by seven, fundal height (FH) by four, and a combination of LMP/FH by nine. Fifty-two studies (42.6%) did not report how it was measured (presumably by LMP/FH). For the remaining 17 studies, a combination of methods was used, with some reporting the use of an additional scoring system at delivery to verify prematurity (e.g. Ballard 12, Dubowitz 2, Finstrom 1). Gestational age data were not reported in 19 studies (15.6%).

Supplemental Tables

Table S1. Study characteristics of studies with information on outcomes by SP doses

	Author and Publication Year	Country	Region	Time period	Design	Sample size	# of sites	LBW (all) %	Pauci-gravidae % [b]	Definition pauci-gravidae	ITN use %[c]	HIV % [d]	Folate dose (mg)[e]	ANC % [c]	*Pf*Pr$_{2\text{-}10}$[12]	Quality score
1	Accrombessi 2018[13]	Benin	CW	2014-2017	Observational	273	35	9·0	7·7	G1	100	1·5	NA	100.0	20·6	4
2	Aduloju 2013[14]	Nigeria	CW	2011-2011	Observational	4200	1	NA	20·7	G1	10·3	4·1 (UNAIDS)	NA	100·0	28·7	2
3	Agomo 2011[15]	Nigeria	CW	2009-2009	Trial	259	4	4·7	39·8	G1	11·1	0·0	NA	100·0	12·6	5
4	Agyeman 2021[16]	Ghana	CW	2016-2017	Observational	1181	4	19·0	29·6	G1	44·3	0·0	NA	100·0	9·2	5
5	Akinnawo 2022[17]	Ghana	CW	2008-2011	Observational	1823	NA	NA	19·3	G1	51·7	2·1 (UNAIDS)	NA	95·0	69·5	4
6	Alli 2013[18]	Nigeria	CW	2010-2011	Observational	200	1	2·0	35·0	G1	19·5	4·1 (UNAIDS)	NA	100·0	24·6	3
7	Anchang Kimbi 2009[19]	Cameroon	CW	2007-2007	Observational	300	1	NA	31·0	G1	6·6	5·6	NA	98·1	47·2	3
8	Anchang-Kimbi 2020[20]	Cameroon	CW	2016-2017	Observational	465	4	7·3	29·9	G1	67·7	3·4 (UNAIDS)	NA	99·4	25·6	5
9	Anto 2019[21]	Ghana	CW	2017-2017	Observational	254	1	17·7	34·3	G1	95·3	2·3	NA	99·2	32·0	4
10	Apinjoh 2015[22]	Cameroon	CW	2008-2010	Observational	411	1	NA	32·0	G1	9·8	4·0	NA	100·0	33·6	4
11	Apinjoh 2022[23]	Cameroon	CW	2019-2020	Observational	454	1	3·1	29·1	G1	65·4	3·7 (UNAIDS)	NA	100·0	23·6	3
12	Appiah 2020[24]	Ghana	CW	2014-2015	Observational	2012	2	NA	31·1	G1	55·6	2·0	NA	100·0	17·0	2
13	Arinaitwe 2013[25] [f] [g]	Uganda	ES	2011-2011	Observational	552	1	9·8	32·4	G1	87·8	0·0	5·0	94·3	38·0	4
14	Arnaldo 2018[26]	Mozambique	ES	2014-2015	Observational	918	5	7·7	38·3	G1	92·5	0·0	NA	99·4	11·3	5
15	Asamoah 2018[27]	Ghana	CW	2016-2016	Observational	100	2	14·0	33·0	G1	86·0	2·4 (UNAIDS)	NA	100.0	15·6	3
16	Aziken 2011[28]	Nigeria	CW	2009-2009	Observational	741	1	10·1	18·9	G1	0·0	0·0	NA	100·0	44·3	2
17	Bedia-Tanoh 2021[29]	Cote d'Ivoire	CW	2017-2017	Observational	197	2	0·0	20·3	G1	49·2	3·6 (UNAIDS)	NA	NA	20·1	4
18	Biaou 2019[30]	Benin	CW	2017-2017	Observational	567	30	NA	22·0	G1	81·7	1·2 (UNAIDS)	NA	100·0	23·5	4
19	Bouyou-Akotet 2010[31]	Gabon	CW	2005-2006	Observational	203	1	17·2	77·3	G1	37·0	5·4 (UNAIDS)	NA	91·8	9·8	3
20	Bouyou-Akotet 2016[32] [f]	Gabon	CW	2011-2011	Observational	299	2	6·0	19·1	G1	16·2	0·0	NA	100·0	23·5	4
21	Braun 2015[33] [g]	Uganda	ES	2013-2013	Observational	624	1	9·6	31·7	G1	65·1	0·0	NA	95·9	13·5	4
22	Cassam 2007[34]	Mozambique	ES	2005-2007	Observational	15,295	50	8·1	27·5	G1	43·7	36·4	NA	100·0	15·1	4
23	Challis 2004[35]	Mozambique	ES	2001-2002	Trial (IPTp) [h]	403	2	11·4	100·0	G1/G2	1·0	10·0	NA	100·0	32·9	5
24	Chico 2017[36]	Zambia	ES	2012-2014	Observational	716	2	21·9	26·8	G1	37·8	12·8	NA	100·0	37·5	3
25	Chukwuocha 2016[37]	Nigeria	CW	2014-2014	Observational	230	1	NA	36·5	G1	19·7	3·2 (UNAIDS)	NA	100·0	5·9	3
26	Cohee 2014[38]	Malawi	ES	2009-2011	Observational	447	1	18·0	100·0	G1/G2	44·8	0·0	NA	100·0	23·7	5

	Author and Publication Year	Country	Region	Time period	Design	Sample size	# of sites	LBW (all) %	Pauci-gravidae %[b]	Definition pauci-gravidae	ITN use %[c]	HIV %[d]	Folate dose (mg)[e]	ANC %[c]	*Pf*Pr$_{2-10}$[12]	Quality score
27	Cosmic 2018[39]	Burkina Faso	CW	2014-2016	Trial[h]	429	15	12·4	20·1	G1	73·2	0·9 ((UNAIDS)	NA	100·0	30·6	5
	Cosmic 2018[39]	The Gambia	CW	2013-2015	Trial	900	15	12·6	16·7	G1	80·2	1·9 (UNAIDS)	NA	100·0	24·4	
	Cosmic 2018[39]	Benin	CW	2014-2016	Trial	952	15	11·7	17·5	G1	72·7	1·1 (UNAIDS)	NA	100·0	35·0	
28	Coulibaly 2014[40, f, g]	Burkina Faso	CW	2010-2012	Observational	912	5	15·1	20·6	G1	80·3	0·0	0·4	100.0	37·3	4
29	Desai 2015[41]	Kenya	ES	2012-2014	Trial[h]	514	4	5·0	34·6	G1	71·4	0·0	NA	100·0	13·8	5
30	Desai 2015[42, f, g]	Kenya	ES	2011-2012	Observational	869	3	7·9	40·0	G1	98·0	0·0	0·4	95·1	42·5	4
31	Diakite 2011 (Maiga)[43]	Mali	CW	2006-2008	Trial	814	2	9·9	41·8	G1/G2	16·7	1·4 (UNAIDS)	0·4	100·0	27·0	5
32	Dosoo 2021[44]	Ghana: Dodowa	CW	2017-2019	Observational	404	1	5·2	13·9	G1	52·0	0·0	NA	100·0	16·2	5
	Dosoo 2021[44]	Ghana: Kintampo	CW	2017-2019	Observational	1128	1	7·0	23·4	G1	63·6	0·0	NA	100·0	17·2	
	Dosoo 2021[44]	Ghana: Navrongo	CW	2017-2019	Observational	394	1	10·7	29·4	G1	80·0	0·0	NA	100·0	8·0	
33	Douamba 2014[45]	Burkina Faso	CW	2013-2014	Observational	238	1	NA	21·3	G1[g]	86·6	.9 (UNAIDS)	NA	98·5	13·6	2
34	Epuitai 2019[46]	Uganda	ES	2018-2019	Observational	366	1	NA	34·1	G1	80·3	10·4	NA	98·4	35·8	2
35	Falade 2007[47]	Nigeria	CW	2003-2004	Observational	769	1	6·1	23·5	G1	1·1	2·0	5·0	91·9	38·8	3
36	Famanta 2011[48, f]	Mali	CW	2009-2009	Observational	359	1	11·4	27·5	G1	80·7	1·3 (UNAIDS)	NA	72·8	6·4	2
37	Fehintola 2015[49]	Nigeria	CW	2013-2013	Observational	300	2	NA	40·3	G1	27·0	4·0	NA	85·3	35·5	2
38	Feng 2010[50, f]	Malawi	ES	1997-1999	Observational	912	1	14·6	46·4	G1/G2	10·0	16·6 (UNAIDS)	NA	76·2	24·5	2
	Feng 2010[50, f]	Malawi	ES	1999-2001	Observational	837	1	12·7	48·4	G1/G2	23·0	16·5 (UNAIDS)	NA	97·9	47·9	
	Feng 2010[50, f]	Malawi	ES	2002-2006	Observational	2370	1	10·1	47·5	G1/G2	51·0	14·5 (UNAIDS)	NA	97·3	47·9	
39	Filler 2006[51]	Malawi	ES	2002-2005	Observational	432	1	21·3	100·0	G1/G2	16·2	0·0	0·5	100·0	39·9	5
40	Gies 2009[52]	Burkina Faso	CW	2004-2006	Trial (cluster)	1272	12	17·5	100·0	G1/G2	5·3	1·4 (UNAIDS)	NA	95·3	49·4	5
41	Gutman 2013[53]/Kalilani 2014[54, f, g,]	Malawi	ES	2009-2011	Observational	1832	4	7·2	31·6	G1	67·0	0·0	0·4	95·4	23·3	4
42	Gutman 2016[55]	Malawi	ES	2015-2015	Observational	536	2	8·3	35·7	G1	52·2	3·6	NA	100·0	22·2	5
43	Harrington 2011[56, f]	Tanzania	ES	2002-2005	Observational	372	1	4·6	29·2	G1	15·5	6·9 (UNAIDS)	NA	100·0	51·3	4
44	Hommerich 2007[57]	Ghana	CW	2006-2006	Observational	839	1	12·4	32·7	G1	8·0	3·0	NA	97·3	28·1	3
45	Igboeli 2017[58]	Nigeria	CW	2013-2013	Observational	416	1	3·6	30·2	G1	20·0	3·4 (UNAIDS)	NA	100·0	8·3	3
46	Igboeli 2018[59]	Nigeria	CW	2016-2016	Trial	210	1	8·0	29·5	G1	86·4	0·0	5·0	100·0	8·5	4

	Author and Publication Year	Country	Region	Time period	Design	Sample size	# of sites	LBW (all) %	Pauci-gravidae % [b]	Definition pauci-gravidae	ITN use %[c]	HIV % [d]	Folate dose (mg)[e]	ANC % [c]	*Pf*Pr$_{2-10}$[12]	Quality score
47	Inyang-Etoh 2011[60]	Nigeria	CW	2008-2008	Observational	640	1	NA	24·4	G1 [g]	7·2	3·1 (UNAIDS)	NA	100·0	51·5	3
48	Isah 2017[61]	Nigeria	CW	2013-2014	Trial	377	1	14·3	33·4	G1	12·7	1·7 (UNAIDS)	5·0	100·0	11·8	4
49	Kajubi 2019[62]	Uganda	ES	2016-2018	Trial [h]	391	1	6·6	26·1	G1	99·0	0·0	NA	100·0	29·7	6
50	Kalayjian 2013[63]	Kenya	ES	2006-2009	Observational	479	1		25·6	G1	75·1	6·9 (UNAIDS)	NA	100·0	11·1	4
51	Kalilani 2010[64]	Malawi	ES	2002-2003	Observational	1151	2	8·7	26·4	G1	15·0	19·5 (UNAIDS)	NA	100·0	30·6	4
52	Kamau 2022[65]	Kenya: Kilifi North	ES	2015-2021	Observational	19,542	1	13·5	37·4	G1	72·0	3·7	NA	99·7	6·7	2
	Kamau 2022[65]	Kenya: Kilifi South	ES	2015-2021	Observational	6620	1	17·0	42·4	G1	72·0	3·9	NA	99·7	8·4	
53	Kayentao 2014[66] [f g]	Mali: Koro	CW	2006-2007	Observational	351	1	7·7	27·8	G1	58·7	1·3 (UNAIDS)	0·4	46·6	65·6	4
	Kayentao 2014[66] [f g]	Mali: San	CW	2006-2006	Observational	398	1	7·3	23·6	G1	61·3	1·3 (UNAIDS)	0·4	68·5	74·8	
	Kayentao 2014[66] [f g]	Mali: Bougouni	CW	2006-2007	Observational	407	1	6·9	23·5	G1	35·9	1·3 (UNAIDS)	0·4	70·5	44·7	
	Kayentao 2014[66] [f g]	Mali: Djenne	CW	2006-2006	Observational	355	1	6·5	22·1	G1	67·9	1·3 (UNAIDS)	0·4	51·7	70·0	
	Kayentao 2014[66] [f g]	Mali: Kita	CW	2009-2010	Observational	544	1	10·3	25·8	G1	88·3	1·3 (UNAIDS)	0·4	24·1	69·2	
	Kayentao 2014[66] [f g]	Mali: San	CW	2009-2010	Observational	430	1	9·3	20·2	G1	94·5	1·3 (UNAIDS)	0·4	51·7	70·0	
54	Kayiba 2021[67]	DRC	CW	2019-2020	Observational	844	1	6·2	22·9	G1	50·1	0·0	NA	98·7	11·1	4
55	Kilauzi 2013[68]	DRC	CW	2011-2011	Observational	705	1	7·5	20·0	G1 [g]	43·8	1·1 (UNAIDS)	NA	96·4	7·7	3
56	Lash 2020[69]	Tanzania	ES	2019-2019	Observational	912	40	4·6	20·7	G1	90·4	6·2 (UNAIDS)	NA	98·6	5·0	4
57	Likwela 2012[70]	DRC: Mikalayi	CW	2007-2007	Observational	477	1	16·1	17·2	G1	6·5	1·5 (UNAIDS)	NA	100·0	79·7	4
58	Likwela 2012[70]	DRC: Kisangani	ES	2007-2007	Observational	137	1	7·8	29·2	G1	4·7	1·5 (UNAIDS)	NA	100·0	78·5	
	Likwela 2012[70]	DRC: Rutshuru	ES	2007-2007	Observational	670	1	8·2	16·4	G1	11·3	1·5 (UNAIDS)	NA	100·0	13·5	
59	Lingani 2022[71]	Burkina Faso	CW	2019-2020	Observational	600	4	11·0	33·1	G1	92·9	0·7	NA	98·0	17·1	3
	Luntamo 2012[72]	Malawi	ES	2003-2007	Trial [h]	877	1	10·1	24·0	G1	72·9	14·1	0·25	100·0	39·8	6
61	MacArthur 2007[73]	Tanzania	ES	2003-2006	Trial [h]	799	2	NA	100	G1/G2	37·0	6·6 (UNAIDS)	0·4	100·0	39·6	4
62	Mace 2015[74] [f g]	Zambia	ES	2009-2010	Observational	423	2	7·1	36·7	G1	55·5	0·0	5·0	100·0	35·0	4
63	Madanitsa 2016[75]	Malawi	ES	2011-2013	Trial [h]	936	3	10·8	61·8	G1/G2	99·8	0·0	NA	100·0	16·2	4
64	Madanitsa 2023[76]	Kenya	ES	2018-2019	Trial [h]	495	4	5·8	36·0	G1	89·9	0·0	0·5	100·0	5·3	6

	Author and Publication Year	Country	Region	Time period	Design	Sample size	# of sites	LBW (all) %	Pauci-gravidae % [b]	Definition pauci-gravidae	ITN use % [c]	HIV % [d]	Folate dose (mg) [e]	ANC % [c]	*Pf*Pr$_{2-10}$[12]	Quality score
	Madanitsa 2023[76]	Malawi	ES	2018-2019	Trial [h]	469	5	10·8	32·3	G1	62·5	0·0	0·5	100·0	17·8	
	Madanitsa 2023[76]	Tanzania	ES	2018-2019	Trial [h]	595	3	10·4	27·6	G1	78·1	0·0	0·5	100·0	7·8	
65	Mahamar 2021[77]	Mali	CW	2010-2014	Observational	1885	1	9·0	23·6	G1	51·2	0·0	NA	100·0	53·8	5
66	Malpass 2020[78 f]	Malawi	ES	2020-2020	Observational	763	2	10·2	33·9	G1	98·0	7·7	NA	99·8	16·1	3
67	Massamba 2022[79]	Congo	CW	2014-2015	Observational	371	1	8·9	41·1	G1/G2	67·3	3·5 (UNAIDS)	NA	83·4	27·1	2
68	Matambisso 2024[80]	Mozambique	ES	2016-2019	Observational	4016	3	6·0	34·3	G1	84·7	0·0	NA	100·0	5·3	4
69	Mbaye 2006[81]	The Gambia	CW	2002-2004	Trial (IPTp)	1454	14	5·9	0·0	G1	70·3	0·5	0·4	100·0	8·4	6
70	Menendez 2008[82]	Mozambique	ES	2003-2005	Trial (IPTp)	793	1	11·3	25·7	G1	91·5	23·9 (UNAIDS)	0·4	100·0	28·8	6
71	Mikomangwa 2020[83]	Tanzania	ES	2018-2018	Observational	1161	1	5·3	38·4	G1	98·1	0·0	0·4	100·0	2·7	5
72	Minja 2013[84]	Tanzania	ES	2008-2010	Observational	722	1	6·5	21·6	G1	94·9	5·8 (UNAIDS)	NA	100·0	8·4	3
73	Mlugu 2021[85,86]	Tanzania	ES	2018-2018	Observational	500	1	10·9	47·7	G1/G2	97·1	0·0	0·4	100·0	4·1	6
74	Moleins 2010[87]	Senegal	CW	2007-2008	Observational	151	1	7·9	27·3	G1 [g]	45·7	0·8 (UNAIDS)	NA	100·0	0·6	3
75	Mosha 2014[88]	Tanzania	ES	2012-2012	Observational	350	2	5·1	37·4	G1	94·6	3·4	NA	100·0	17·1	4
76	Moukoko 2023[89]	Cameroon	CW	2015-2016	Observational	182	3	9·3	35·8	G1	72·0	0·0	NA	100·0	9·2	4
77	Msyamboza 2009[90]	Malawi	ES	2002-2004	Observational	1318	26	16·8	29·4	G1	10·2	15·2 (UNAIDS)	NA	87·3	37·2	4
78	Muchekeza 2018[91]	Zimbabwe	ES	2011-2011	Observational	300	2	36·7	29·5	G1	58·7	0·0	NA	100·0	15·0	5
79	Muhammad 2016[92]	Nigeria	CW	2014-2014	Observational	184	1	37·0	62·0	G1/G2	89·7	3·2 (UNAIDS)	NA	100·0	21·0	4
80	Mwangi 2015[93]	Kenya	ES	2011-2013	Trial (Iron)	233	4	NA	18·1	G1	15·5	21·1	NA	96·6	16·5	6
81	Mwapasa 2004[94]	Malawi	ES	2000-2002	Observational	1177	1	NA	42·2	G1	22·3	0·0	5·0	97·9	46·8	4
82	Namusoke 2010[95 f]	Uganda	ES	2004-2005	Observational	321	1	14·6	49·4	G1	32·0	11·0	NA	96·8	19·1	4
83	Ndeserua 2015[96]	Tanzania	ES	2012-2012	Observational	350	1	6·3	33·1	G1	97·7	1·7	NA	97·0	17·1	4
84	Nduka 2011[4]	Nigeria	CW	2009-2009	Observational	844	3	NA	35·5	G1	12·0	4 (UNAIDS)	NA	87·0	35·2	2
85	Ndyomugyenyi 2011[97]	Uganda	ES	2004-2007	Trial (IPTp) [h]	3138	10	6·6	21·1	G1	97·0	6·5 (UNAIDS)	5·0	100·0	0·0	6
86	Nganda 2004[98]	Tanzania	ES	2003-2003	Observational	293	1	NA	42·3	G1	48·1	6·8 (UNAIDS)	NA	100·0	37·6	3
87	Njagi 2002[99]	Kenya	ES	1997-1999	Trial (IPTp)	728	1	13·3	100·0	G1/G2	50·0	22·4 (UNAIDS)	5·0	100·0	51·8	5
88	Obi 2022[100]	Nigeria	CW	2021-2021	Observational	483	4	NA	20·1	G1	29·4	1·3 (UNAIDS)	NA	100	17·0	3
89	Oduro 2010[101]	Ghana	CW	2006-2007	Observational	2277	6	18·4	24·2	G1	53·6	2·2 (UNAIDS)	NA	97·0	41·8	3
90	Olliaro 2008[102]	Senegal	CW	2000-2007	Observational	904	1	9·5	21·7	G1 [g]	12·4	.8 (UNAIDS)	NA	100·0	0·2	4

	Author and Publication Year	Country	Region	Time period	Design	Sample size	# of sites	LBW (all) %	Pauci-gravidae % [b]	Definition pauci-gravidae	ITN use % [c]	HIV % [d]	Folate dose (mg) [e]	ANC % [c]	*Pf*$Pr_{2\text{-}10}$ [12]	Quality score
91	Olorunda 2013[103]	Nigeria	CW	2010-2010	Observational	330	1	7·9	37·2	G1	13·9	4·1 (UNAIDS)	NA	100·0	34·8	4
92	Onoja 2021[104]	Nigeria	CW	2018-2018	Observational	300	1	2·0	24·7	G1	24·0	1·6 (UNAIDS)	NA	100·0	20·2	3
93	Onyebuchi 2014[105]	Nigeria	CW	2012-2012	Observational	516	1	NA	45·2	G1/G2	100·0	3·4 (UNAIDS)	NA	100·0	27·1	4
94	Orobaton 2016[106]	Nigeria	CW	2014-2015	Observational	6720	4	NA	18·3	G1	56·1	3·1 (UNAIDS)	NA	56·6	57·6	4
95	Ouma 2012[107]	Kenya	ES	2008-2009	Observational	966	2	7·3	43·9	G1	50·5	0·0	NA	91·0	36·3	3
96	Oweisi 2018[108]	Nigeria	CW	2016-2016	Observational	205	1	NA	27·8	G1	50·2	1·5 (UNAIDS)	NA	88·3	15·3	4
97	Parise 1998[109]	Kenya	ES	1994-1996	Trial (IPTp)	996	1	10·5	100·0	G1/G2	1·0	26·9	5·0	100·0	33·7	4
98	Ramharter 2007[110]	Gabon	CW	2005-2006	Observational	693	3	10·2	28·7	G1	38·1	7·9	NA	90·7	11·9	3
99	Rogawski 2012[111]	Malawi	ES	1997-2006	Observational	3848 [i]	1	NA	47·8	G1	37·0	15·5 (UNAIDS)	NA	90·5	46·8	4
100	Rogerson 2000[112]	Malawi	ES	1997-1999	Observational	1397 [i]	1	NA	46·0	G1	7·0	16·6 (UNAIDS)	0·25	100·0	47·9	2
101	Shulman 1999[113]	Kenya	ES	1996-1997	Trial	1264	2	NA	100·0	G1/G2	23·8	5·5	NA	100·0	32·0	6
102	Sirima 2006[114] [f]	Burkina Faso	CW	2004-2004	Observational	1120	2	12·1	31·1	G1	35·3	1·6 (UNAIDS)	0·25	83·4	28·7	3
103	Stephens 2017[115]	Ghana	CW	2008-2009	Observational	121	1	3·3	31·2	G1	5·4	2·3 (UNAIDS)	NA	100·0	6·6	3
104	Suleiman 2003[116]	Sudan	ES	1999-2001	Observational	110	2	19·1	100	G1	1·0	0·1 (UNAIDS)	NA	100·0	2·6	4
105	Tagbor 2015[117]	The Gambia	CW	2010-2012	Trial [h]	625	1	17·3	100·0	G1/G2	100	0·0	0·4	100·0	7·9	5
	Tagbor 2015[117]	Ghana	CW	2010-2012	Trial [h]	653	1	19·5	100·0	G1/G2	100	0·0	0·4	100·0	60·7	
	Tagbor 2015[117]	Burkina Faso	CW	2010-2012	Trial [h]	720	1	19·5	100·0	G1/G2	100	0·0	0·4	100·0	47·3	
	Tagbor 2015[117]	Mali	CW	2010-2012	Trial [h]	680	3	13·9	100·0	G1/G2	100	0·0	0·4	100·0	36·4	
106	Tetteh-Ashong 2005[118]	Malawi	ES	2005-2005	Observational	228	1	8·3	27·6	G1	17·6	0·1 (UNAIDS)	0·25	90·9	5·1	3
107	Tonga 2013[119]	Cameroon	CW	2011-2012	Observational	195	5	16·7	22·5	G1	19·3	6·0	NA	97·4	26·8	2
108	Tongo 2011[120]	Nigeria	CW	2007-2008	Observational	796	2	9·0	11·6	G1	20·1	3·1 (UNAIDS)	NA	87·1	43·1	2
109	Toure 2014[121]	Cote d'Ivoire	CW	2009-2010	Observational	1312	6	8·5	24·0	G1	16·7	4·0	NA	98·6	43·4	4
110	Toure 2019[122]	Guinea: Kankan	CW	2017-2017	Observational	250	1	16·8	40·4	G1	56·8	1·0 (UNAIDS)	NA	100·0	19·1	2
	Toure 2019[122]	Guinea: Forecariah	CW	2017-2017	Observational	250	1	15·6	46·8	G1	42·8	1·4 (UNAIDS)	NA	98·9	44·0	
	Toure 2019[122]	Guinea: Nzerekoro	CW	2017-2017	Observational	250	1	6·4	26·4	G1	46·4	1·2 (UNAIDS)	NA	92·0	38·0	
	Toure 2019[122]	Guinea: Gueckedou	CW	2017-2017	Observational	250	1	6·4	36·8	G1	69·2	1·2 (UNAIDS)	NA	100·0	22·8	

	Author and Publication Year	Country	Region	Time period	Design	Sample size	# of sites	LBW (all) %	Pauci-gravidae % [b]	Definition pauci-gravidae	ITN use % [c]	HIV % [d]	Folate dose (mg) [e]	ANC % [c]	*Pf*Pr$_{2\text{-}10}$[12]	Quality score
111	Tshiongo 2024[123]	DRC	CW	2018-2018	Observational	467	1	31·2	38·8	G1	52·6	2·9 (UNAIDS)	NA	97·0	17·6	5
112	Tutu 2011[124]	Ghana	CW	2005-2007	Observational	2583	6	12·1	24·3	G1	26·5	2·3 (UNAIDS)	NA	97·3	29·8	4
113	Umemmuo 2020[125]	Nigeria	CW	2017-2017	Observational	426	1	NA	53·5	G1	34·7	1·9 (UNAIDS)	NA	100·0	16·2	5
114	Valea 2010[126]	Burkina Faso	CW	2006-2008	Trial	1296	2	NA	41·4	G1/G2	38·3	1·2 (UNAIDS)	0·4	100·0	64·1	5
115	van Eijk 2004[127 f]	Kenya	ES	1999-2000	Observational	1873	1	9·7	50·2	G1	7·4	14·8 (UNAIDS)	5·0	100·0	39·6	5
116	Van Spronsen 2012[128]	Ghana	CW	2010-2010	Observational	98	1	NA	34·0	G1	26·5	0·0	NA	100·0	69·6	1
117	Vanga-Bosson 2011[129]	Cote d'Ivoire	CW	2008-2008	Observational	1945	6	10·6	16·1	G1	48·0	5·4	NA	97·8	58·4	5
118	Verhoeff 1998[130]	Malawi	ES	1993-1994	Observational	310	1	NA	30·0	G1	1·0	0·0	NA	100·0	61·2	3
119	Vincenz 2022[131]	Mali	CW	2011-2019	Observational	317	1	25·3	44·5	G1	92·0	0·4	0·4	93·4	38·4	2
120	Waltmann 2022[132]	Malawi	ES	2017-2018	Trial [h]	301	1	7·8	34·1	G1	73·3	0·0	0·4	100·0	25·6	5
121	Wolf 2021[133]	Benin	CW	2020-2020	Observational	1259	40	11·5	20·6	G1	92·0	0·5 (UNAIDS)	NA	97·0	34·8	4
122	Yussuf 2010[134]	Tanzania	ES	2009-2010	Observational	246	1	40·2	50·4	G1	91·5	4·0	NA	65·0	15·3	4

Abbreviations (alphabetical order): CW, central and west African region. ES, east and southern African region. *dhps*, dihydropteroate synthetase; G1, G2, first and second pregnancies; G3+, 3 or more previous pregnancies; NA, not available; *Pf*Pr2-10, *P. falciparum* parasite prevalence in children aged 2-10 years; NP: not published; SP, sulfadoxine-pyrimethamine; UNAIDS: Joint United Nations Programme on HIV and AIDS

Notes

a. Outcomes considered: maternal malaria at the time of delivery (any test), placental malaria (any test), low birth weight, preterm delivery, anaemia (haemoglobin < 11 g/dl), birth weight, gestational age and haemoglobin. Additionally, information was collected on placental histology, cord malaria, neonatal malaria, stillbirths/perinatal deaths, and infant anthropometry at delivery.
b. The proportion of primigravidae among the study population was not reported in some studies; the best estimate was obtained from a Demographic and Health Survey (DHS) close in time and location for the following studies: Douambo *et al*. (2014): DHS 2010 Burkina Faso, Inyang-Etoh *et al*. (2011): DHS 2008 Nigeria, Kilauzi *et al.* (2013): DHS DRC 2007 DRC & DHS 2013-2014 (midpoint), Moleins *et al.* (2010): DHS 2005 Senegal, and Olliaro *et al.* (2008): DHS 2005 Senegal.
c. If ITN data were not reported in the study sample, DHS or MIS survey data were used instead, matched closest in time and location. If survey data for a particular year were not available, the nearest value was recorded. If data from two surveys were available (i.e., the nearest survey before and after the start and completion of the study), a linear trend was assumed between the two coverage estimates of the two surveys. If ITN data was not available, then bed net data was used. Information on ITN or bednet use was commonly not reported for studies conducted prior to 2001 and if so, coverage of 1% was assumed in the analyses. If antenatal visits were not reported in the study sample, DHS or other national survey data were used instead, matched closest in time and location; procedures were the same as described for ITNs.
d. Where data was not available for HIV-negative women only, the HIV status prevalence was reported as available in the study. If this was not available, HIV prevalence data among adult women was obtained from UNAIDS for year and country among female adults 15-49 years.[135]
e. Folic acid dose used in the antenatal clinic as reported in the source study article
f. Data was supplemented with information from the authors.
g. These studies were part of the 'IPTp-Mon' study, a multi-country observational study specifically designed to address the relationship between the population level of SP resistance and IPTp-effectiveness.[42] The study used a common protocol and data sets were available to the current study.

h. Trials with arms excluded (10 of 22 trials or 45.5%): Cosmic 2018: arm with intermittent screening and treatment (IST) by community health workers excluded. Desai 2015, Kajubi 2019, Madanitsa 2016 & Madanitsa 2023, Waltmann 2022, Tagbor 2015: arms with dihydroartemisin-piperaquine (DP) and/or IST were excluded. Luntamo 2012: IPT arm with azithromycin excluded. MacArthur 2007: arm with artesunate excluded. Ndyomugyenyi 2011: arm with IPTp only excluded (included ITN+IPTp with SP versus ITN+IPTp with placebo).

i. The studies by Rogawski 2012 and Rogerson 2000 were excluded from the total number of enrolled participants because the data of these studies were included in Feng 2010

Table S2. Matching of studies with information on SP resistance markers (*Pfdhps*-A437G, *Pfdhps*-K540E and *Pfdhps*-A581G)

	IPTp study Author, Publication Year	Study site, country	Time period study	*Pfdhps* A437G %	Distance in km (location match)	Years difference (study period match)	*Pfdhps* K540E %	Distance in km (location match)	Years difference (study period match)	Sco re*	*Pfdhps* A581G %	Distance in km (location match)	Years difference (study period match)	Score*	Sample size (N) of marker study
1	Accrombessi 2018[13]	So-Ava, Abomey-Calavi districts, Benin	2014-2017	98·4[136]	~40 (Allada)	-2 (2010-2012)	0·0	~40 (Allada)	-2 (2010-2012)	3	1·6	~40 (Allada)	-2 (2010-2012)		183[136]
2	Aduloju 2013[14]	Ado Ekiti, Nigeria	2011-2011	84·2	0	0	2·2	0	0	3	15·1	0	0		model[6]
3	Agomo 2011[15]	Lagos, Nigeria	2009-2009	37·5[137]	0 (Lagos)	+2 (2011)	0·0[137]	0 (Lagos)	+2 (2011)	3	0·0[138]	~320 (Benin City)	+4 (2014-2015)		40[137], 94[138]
4	Agyeman 2021[16]	Tamale, Ghana	2016-2017	100[139]	0	0 (2017)	2·4[139]	0	0 (2017)	3	2·3[140]	~550 (Begoro)	0 (2017)		41[140]
5	Akinnawo 2022[17]	Kintampo district, Ghana	2008-2011	86·1	0	0	0·5	0	0	3	0·9	0	0		model[6]
6	Alli 2013[18]	Kubwa, Nigeria	2010-2011	70·5	0	0	1·7	0	0	3	10·5	0	0		model[6]
7	Anchang Kimbi 2009[19]	Mutengene, Cameroon	2007-2007	85·5[141]	0 (Mutengene)	-1 (2004-2006)	0·5[141]	0 (Mutengene)	-1 (2004-2006)	3	2·0[141]	0 (Mutengene)	-1 (2004-2006)		200[141]
8	Anchang-Kimbi 2020[20]	Mount Cameroon area, Cameroon	2016-2017	90·0[142]	~300 (Dschang)	0 (2017)	4·4	~300 (Dschang)	0 (2017)	2	7·1	~300 (Dschang)	0 (2017)		68[142]
9	Anto 2019[21]	Navrongo, Ghana	2017-2017	100[139]	~190 (Tamale)	0 (2017)	2·4[139]	~190 (Tamale)	0 (2017)	3	2·3[140]	~750 (Begoro)	0 (2017)		33-43[139,140]
10	Apinjoh 2015[22]	Mutengene, Cameroon	2008-2010	85·5[141]	0 (Mutengene)	-2 (2004-2006)	0·5[141]	0 (Mutengene)	-2 (2004-2006)	3	2·0[141]	0 (Mutengene)	-2 (2004-2006)		200[141]
11	Apinjoh 2022[23]	Mutengene, Cameroon	2019-2020	88·6[143]	0	0	2·6	0	0	3	5·8	0	0		model[6]
12	Appiah 2020[24]	Bono region, Ghana	2014-2015	100[139]	~130 (Duase)	+2 (2017)	0·0	~130 (Duase)	+2 (2017)	3	5·8[140]	~300 (Begoro)	0 (2015)		45, 69[139,140]
13	Arinaitwe 2013[25]	Tororo, Uganda	2011-2011	97·3[42]	0 (Tororo)	0	97·5[42]	0 (Tororo)	0	3	0·2[42]	0 (Tororo)	0	3	100[42]
14	Arnaldo 2018[26]	Chokwe district, Mozambique	2014-2015	90·8[144]	0	0 (2015)	88·5	0	0 (2015)	3	0[145]	~120 (Palmeira)	+1 (2016)	3	87[145]
15	Asamoah 2018[27]	Asanti region, Ghana	2016-2016	97·7	0	0	1·1	0	0	3	3·5	0	0		model[6]
16	Aziken 2011[28]	Benin City, Nigeria	2009-2009	84·2[138]	~260 (Enugu)	+1 (2010)	0·0[138]	~260 (Enugu)	+1 (2010)	3	47·4[138]	~260 (Enugu)	+1 (2010)		38[138]
17	Bedia-Tanoh 2021[29]	San Pedro, Cote d'Ivoire	2017-2017	92·4	0	0	4·7	0	0	3	1·2	0	0		model[6]
18	Biaou 2019[30]	Cotonou, Benin	2017-2017	94·4	0	0	3·6	0	0	3	9·3	0	0		model[6]
19	Bouyou-Akotet 2010[31]	Libreville, Gabon	2005-2006	78·0	0	0	1·6	0	0	3	0·9	0	0		model[6]
20	Bouyou-Akotet 2016[32]	Libreville, Melen, Gabon	2011-2011	85·9	0	0	2·6	0	0	3	5·7	0	0		model[6]
21	Braun 2015[33]	Fort Portal, Uganda	2013-2013	100[146]	~130 (Kihurura)	0 (2012-2014)	100[146]	~130 (Kihurura)	0 (2012-2014)	3	12·9[146]	~130 (Kihurura)	0 (2012-2014)	3	62[146]
22	Cassam 2007[34]	Gaza, Maputo, Mozambique	2005-2007	53·2[147]	0	0	47·6[147]	0	0	3	0·0[148,149]	0 (Gaza, Maputo)	0 (2006-2007)	3	~ 2700 [148,149]
23	Challis 2004[35]	Matola, Boane, Mozambique	2001-2002	26·1[9]	0 (peri-urban Maputo)	0 (2001)	25·4[9]	0 (peri-urban Maputo)	0 (2001)	3	0·0[150]	0 (Maputo)	0 (1999-2004)	3	134[9] ~1000[150]

	IPTp study Author, Publication Year	Study site, country	Time period study	*Pfdhps* A437G %	Distance in km (location match)	Years difference (study period match)	*Pfdhps* K540E %	Distance in km (location match)	Years difference (study period match)	Score*	*Pfdhps* A581G %	Distance in km (location match)	Years difference (study period match)	Score*	Sample size (N) of marker study
24	Chico 2017[36]	Nchelenge, Zambia	2012-2014	36·1[151]	0	0 (2013)	31·9	0	0 (2013)	3	13·9	0	0 (2013)	3	72[151]
25	Chukwuocha 2016[37]	Owerri, Nigeria	2014-2014	96·8[138]	~230 (Benin City)	0 (2014-2015)	0·0[138]	~230 (Benin City)	0 (2014-2015)	3	52·6[138]	~230 (Benin City)	0 (2014-2015)		95[138]
26	Cohee 2014[38]	Blantyre, Malawi	2009-2011	99·6[152]	0	0 (2012)	99·6	0	0 (2012)	3	2·9	0	0 (2012)	3	546[152]
27	Cosmic 2018[39]	Nanoro, Burkina Faso	2014-2016	34·2[153]	0	-2 (2012)	0·0	0	-2 (2012)	2	2·5[154]	West Africa	0 (2016)		23[153], >14000[154]
	Cosmic 2018[39]	Upper River Region, The Gambia	2013-2015	64·1[155]	0	0	1·3	0	0	3	0·6	0	0		model[6]
	Cosmic 2018[39]	Zinvie, Ze, and Glo-Djigbe, Benin	2014-2016	100[156]	~95 (Klouékanmey)	+1 (2017)	1·5	~95 (Klouékanmey)	+1 (2017)	3	4·4	~95 (Klouékanmey)	+1 (2017)		68[156]
28	Coulibaly 2014[40]	Ziniare, Burkina Faso	2010-2012	75·3[42]	0	0	0·0[42]	0	0	3	0·0[42]	0	0		273[42]
29	Desai 2015[41]	Siaya County, Kenya	2012-2014	93·0	0	0 (2009-2013)	95·6	0	0 (2009-2013)	3	5·7	0	0 (2009-2013)	3	53[41]
30	Desai 2015[42]	Siaya, Kenya	2011-2012	93·0[42]	0	0	95·6[42]	0	0	3	5·7[42]	0	0	3	53[42]
31	Diakite 2011 (Maiga)[43]	Bla, Mali	2006-2008	27·7[157]	~110 (San)	-1 (2009-2010)	0·0	~110 (San)	-1 (2009-2010)	3	0·0	~110 (San)	-1 (2009-2010)		130[157]
32	Dosoo 2021[44]	Dodowa, Ghana	2017-2019	960	0	0	2·9	0	0	3	5·6	0	0		model[6]
	Dosoo 2021[44]	Kintampo, Ghana	2017-2019	100[139]	~170 (Duase)	0 (2017)	0·0	~170 (Duase)	0 (2017)	3	2·3[140]	~350 (Begoro)	0 (2017)		45[139] 43[140]
	Dosoo 2021[44]	Navrongo, Ghana	2017-2019	100[139]	~190 (Pagaza)	0 (2017)	2·4	~190 (Pagaza)	0 (2017)	3	2·3[140]	~750 (Begoro)	0 (2017)		33[139] 43[140]
33	Douamba 2014[45]	Ouagadougou, Burkina Faso	2013-2014	75·3[42]	~ 30 (Ziniare)	-1 (2010-12)	0·0[42]	~ 30 (Ziniare)	-1 (2010-12)	3	0·0[42]	~ 30 (Ziniare)	-1 (2010-12)		273[42]
34	Epuitai 2019[46]	Lira district, Uganda	2018-2019	96·9	0	0	91·2	0	0	3	7·7	0	0	3	model[6]
35	Falade 2007[47]	Ibadan, Nigeria	2003-2004	63·0[138]	0 (Ibadan)	0 (2003)	0·0[138]	0 (Ibadan)	0 (2003)	3	0·0[138]	0 (Ibadan)	0 (2003)		36[138]
36	Famanta 2011[48]	Bamako, Mali	2009-2009	15·2[42]	~190 (Kita)	0 (2009-2010)	0·7[42]	~190 (Kita)	0 (2009-2010)	3	0·0[42]	~190 (Kita)	0 (2009-2010)		117[42]
37	Fehintola 2016[49]	Ile Ife, Nigeria	2013-2013	96·8[138]	~230 (Benin City)	+1 (2014-2015)	0·0[138]	~230 (Benin City)	+1 (2014-2015)	3	52·6[138]	~230 (Benin City)	+1 (2014-2015)		95[138]
38	Feng 2010[50]	Blantyre, Malawi	1997-1999	63·6[147]	0	0	74·0[147]	0	0	3	0·0[158]	0 (Ndirande)	0 (1997-1999)	3	149[158]
	Feng 2010[50]	Blantyre, Malawi	1999-2001	80·3[147]	0	0	84·0[147]	0	0	3	0·0[152]	0 (Ndirande)	0 (1999-2001)	3	550[152]
	Feng 2010[50]	Blantyre, Malawi	2002-2006	93·5[147]	0	0	95·0[152]	0 (Ndirande)	+1 (2007-2009)	3	2·0[152]	0 (Ndirande)	+1 (2007-2009)	3	556
39	Filler 2006[51]	Machinga, Malawi	2002-2005	89·8[159]	~130 (Lungwena)	0 (2003-2006)	86·4	~130 (Lungwena)	0 (2003-2006)	3	1·1	~130 (Lungwena)	0 (2003-2006)	3	88[159]
40	Gies 2009[52]	Boromo, Burkina Faso	2004-2006	71·5[147]	0	0	0·2[147]	0	0	3	0·0[42]	210 (Ziniare)	+4 (2010)		273[42]
41	Gutman 2013[53]/ Kalilani 2014[54]	Blantyre & Machinga, Malawi	2009-2011	94·4[42]	0	0	99·6[42]	0	0	3	1·5[42]	0	0	3	134[42]

	IPTp study Author, Publication Year	Study site, country	Time period study	*Pfdhps* A437G %	Distance in km (location match)	Years difference (study period match)	*Pfdhps* K540E %	Distance in km (location match)	Years difference (study period match)	Sco re*	*Pfdhps* A581G %	Distance in km (location match)	Years difference (study period match)	Score*	Sample size (N) of marker study
42	Gutman 2016[55]	Machinga, Balaka; Malawi	2015-2015	100·0[55]	0	0	100	0	0	3	2·4	0	0	3	82[55]
43	Harrington 2011[56]	Muheza, Tanzania	2002-2005	85·6s	0	0	85·2	0	0	3	15·2	0	0	3	model[6]
44	Hommerich 2007[57]	Agogo, Ghana	2006-2006	84·6[147]	0	0	1·4[147]	0	0	3	0·0[160]	~90 (Bekwai)	+1 (2007-2008)		35[160]
45	Igboeli 2017[58]	Enugu State, Nigeria	2013-2013	96·8[138]	~ 260 (Benin City)	+1 (2014-2015)	0·0[138]	~ 260 (Benin City)	+1 (2014-2015)	3	52·6[138]	~ 260 (Benin City)	+1 (2014-2015)		95[138]
46	Igboeli 2018[59]	Nsukka, Nigeria	2016-2016	57·3[161]	~200 (Imo)	-1 (2014-2015)	0·0	~200 (Imo)	-1 (2014-2015)	3	0·0	~200 (Imo)	-1 (2014-2015)		82[161]
47	Inyang-Etho 2011[60]	Calabar, Nigeria	2008-2008	84·2[138]	~ 260 (Enugu)	+2 (2010)	0·0[138]	~ 260 (Enugu)	+2 (2010)	3	47·4[138]	~ 260 (Enugu)	+2 (2010)		38[138]
48	Isah 2017[61]	Abuja, Nigeria	2013-2014	91·7	0 (Abuja)	0 (2013-2016)	11·1	0 (Abuja)	0 (2013-2016)	3	13·9	0 (Abuja)	0 (2013-2016)		36[162]
49	Kajubi 2019[62]	Busia, Uganda	2016-2018	98·6	~55 (Tororo)	-1 (2014-2015)	84·9	~55 (Tororo)	-1 (2014-2015)	3	0	~55 (Tororo)	-1 (2014-2015)	3	365-380[163]
50	Kalayjian 2013[63]	Msambweni, Kenya	2006-2009	79·3	~195 (Malindi)	+1 (2010)	75·0	~195 (Malindi)	+1 (2010)	2	0	~195 (Malindi)	+1 (2010)	2	28[164]
51	Kalilani 2010[165]	Mpemba, Madziabango, Malawi	2002-2003	80·3[147]	0	0	84·0[152]	~30 (Ndirande)	-1 (1999-2001)	3	0·0[152]	~30 (Ndirande)	-1 (1999-2001)	3	550[152]
52	Kamau 2022[65]	Kilifi, Kenya	2015-2021	96·5[166]	0 (Kilifi)	0 (2015-2018)	95·1	0 (Kilifi)	0 (2015-2018)	3	0·4	0 (Kilifi)	0 (2015-2018)	3	226[166]
53	Kayentao 2014[66]	Koro, Mali	2006-2007	44·8[147]	0	0	0·1[147]	0	0	3	0·0[42]	~200 (San)	+3 (2010)		130[42]
	Kayentao 2014[66]	San, Mali	2006-2006	32·6[147]	0	0	0·0[147]	0	0	3	0·0[42]	0 (San)	+4 (2010)		130[42]
	Kayentao 2014[66]	Bougouni, Mali	2006-2007	33·8[147]	0	0	0·2[147]	0	0	3	0·0[42]	~650 (San)	+3 (2010)		130[42]
	Kayentao 2014[66]	Djenne, Mali	2006-2006	32·7[147]	0	0	0·0[147]	0	0	3	0·0[42]	~130 (San)	+4 (2010)		130[42]
	Kayentao 2014[66]	Kita, Mali	2009-2010	15·2[42]	0	0	0·7[42]	0	0	3	0·0[42]	0	0		117[42]
	Kayentao 2014[66]	San, Mali	2009-2010	27·5[42]	0	0	0·0[42]	03	0	3	0·0[42]	0	0		130[42]
54	Kayiba 2021[67]	Kinshasa, DRC	2019-2020	79·1[67]	0	0 (2019-2020)	32·6	0	0 (2019-2020)	3	6·7[167]	0	-4 (2014-2015)		14-34[67], 577[167]
55	Kilauzi 2013[68]	Kinshasa, DRC	2011-2011	100·0[146]	0 (Kinshasa)	+1 (2012-2014)	18·9[146]	0 (Kinshasa)	+1 (2012-2014)	3	8·1[146]	0 (Kinshasa)	+1 (2012-2014)		37[146]
56	Lash 2020[69]	Geita, Tanzania	2019-2019	78·8	0	0	77·5	0	0	3	2·2	0	0	3	model[6]
57	Likwela 2012[70]	Mikalayi, DRC	2007-2007	76·9[147]	0	0	11·3[147]	0	0	3	0·0[8]				model[6,8]
58	Likwela 2012[70]	Kisangani, DRC	2007-2007	72·3[147]	0	0	46·6	0	0	3	10·8	0	0	3	model[6]
	Likwela 2012[70]	Rutshuru, DRC	2007-2007	88·1[168]	~280 (Rukara & Mashesa, Rwanda)	-1 (2005-2006)	91·2[168]	~280 (Rukara & Mashesa, Rwanda)	-1 (2005-2006)	3	45·6[168]	~280 (Rukara & Mashesa, Rwanda)	-1 (2005-2006)	3	Mean†: 776[168]
59	Lingani 2022[71]	Yako Health district, Burkina Faso	2019-2020	79·7[169]	~290 (Hounde)	-3 (2016)	3·0	~290 (Hounde)	-3 (2016)	3	5·6[154]	0	-1 (2018)		64[169] 801[154]
60	Luntamo 2012[72]	Lungwena, Malawi	2003-2007	89·8[159]	0	0 (2003-2006)	86·4	0	0 (2003-2006)	3	1·1	0	0 (2003-2006)	3	88[159]

	IPTp study Author, Publication Year	Study site, country	Time period study	*Pfdhps* A437G %	Distance in km (location match)	Years difference (study period match)	*Pfdhps* K540E %	Distance in km (location match)	Years difference (study period match)	Score*	*Pfdhps* A581G %	Distance in km (location match)	Years difference (study period match)	Score*	Sample size (N) of marker study
61	MacArthur 2007[73]	Kilombera, Tanzania	2003-2006	2·1[170]	~250 (Kibiti, Rufiji)	0 (1998-2003)	4·9	~250 (Kibiti, Rufiji)	0 (1998-2003)	3	0·0	~250 (Kibiti, Rufiji)	0 (1998-2003)	3	339, 82[170]
62	Mace 2015[74]	Mansa, Zambia	2009-2010	83·7[42]	0	0	84·0[42]	0	0	3	0·0[42]	0	0	3	97[42]
63	Madanitsa 2016[75]	South Malawi	2011-2013	99·6[152]	0	0 (2012)	99·6	0	0 (2012)	3	2·9	0	0 (2012)	3	588[152]
64	Madanitsa 2023[76]	Ahero, Kenya	2018-2019	95·4	0	0 (2017)	93·8	0	0 (2018-2019)	3	6·2	0	0 (2018-2019)	3	65[76]
	Madanitsa 2023[76]	Mangochi, Zomba, Mpemba, Madziabango, Malawi	2018-2019	98·6	0	0 (2018-2019)	98·5	0	0 (2018-2019)	3	11·4	0	0 (2018-2019)	3	68-70[76]
	Madanitsa 2023[76]	Korogwe district, Tanzania	2018-2019	94·4	0	0 (2018-2019)	94·3	0	0 (2018-2019)	3	47·9	0	0 (2018-2019)	3	70-72[76]
65	Mahamar 2021[77]	Ouelessebougou, Mali	2010-2014	41·8[171]	~270 (Kita)	0 (2014)	12·5	~270 (Kita)	0 (2014)	3	0·0	0	0 (2009-2010)		48[171], 130[42]
66	Malpass 2020[78]	Nkhata Bay, Malawi	2020-2020	93·0[152]	0	0	95·1	0	0	3	1·2	0	0	3	model[6]
	Malpass 2020[78]	Ntcheu district, Malawi	2020-2020	96·5	0	0	98·2	0	0	3	1·2	0	0	3	model[6]
67	Massamba 2022[79]	Brazzaville, Republic of Congo	2014-2015	96·1[167]	~35 (Kinshasa)	0 (2014-2015)	17·0	~35 (Kinshasa)	0 (2014-2015)	3	10·0	~35 (Kinshasa)	0 (2014-2015)		311[167]
68	Matambisso 2024[80]	Manhica, Mozambique	2016-2019	94·9	0	0	94·9	0	0	3	0·0	0	0	3	131[80]
69	Mbaye 2006[81]	Farafenni, The Gambia	2002-2004	47·6	0	0	0·2	0	0	3	0·2	0	0		model[6]
70	Menendez 2008[82]	Manhica district, Mozambique	2003-2005	62·9[172]	0 (Manhica)	0 (2002-2005)	68·6[172]	0 (Manhica)	0 (2002-2005)	3	0·0[148]	50 (Magude)	0 (2004-2005)	3	70[172]‡ ~500[148]
71	Mikomangwa 2020[83]	Dar es Salaam, Tanzania	2018-2018	89·2[173]	~150 (Kibiti)	+1 (2019)	87·4	~150 (Kibiti)	+1 (2019)	3	1·1	~150 (Kibiti)	+1 (2019)	3	446[173]
72	Minja 2013[84]	Korogwe, Tanzania	2008-2010	96·4	0	0	93·6	0	0	3	49·9	0	0	3	model[6]
73	Mlugu 2020[85]	Kibiti, Tanzania	2017-2019	89·2[173]	0 (Kibiti)	0 (2019)	87·4	0 (Kibiti)	0 (2019)	3	1·1	0 (Kibiti)	0 (2019)	3	446[173]
74	Moleins 2010[87]	Oussouye, Senegal	2007-2008	43·0[147]	0	0	0·06[147]	0	0	3	0·0[174]	~440 (Thies)	0 (2008)		93[174]
75	Mosha 2014[88]	Moshi & Rufiji, Tanzania	2012-2012	77·0	0	0	69·9	0	0	3	1·0	0	0	3	model[6]
76	Moukoko 2023[89]	Douala, Littoral Region, Cameroon	2015-2016	90·5	0	0	2·4	0	0	3	7·8	0	0		model[6]
77	Msyamboza 2009[90]	Chikwawa, Malawi	2002-2004	87·0[147]	0	0	92·7[147]	0	0	3	0·0[175]	~70 (Chileka)	0 (2003-2005)	3	95[175]
78	Muchekeza 2018[91]	Gokwe North, Zimbabwe	2011-2011	65·6	0	0	55·6	0	0	3	0·5	0	0	3	model[6]
79	Muhammad 2016[92]	Nguru, Yobe state, Nigeria	2014-2014	84·1[176]	0	0	3·4[176]	0	0	3	9·3[8]	0	0		model[6]
80	Mwangi 2015[93]	South West Kisumu, Nyanza, Kenya	2011-2013	93·0[42]	~70 (Siaya county)	0 (2011-2012)	95·6[42]	~70 (Siaya county)	0 (2011-2012)	3	5·7[42]	~70 (Siaya county)	0 (2011-2012)	3	53[42]
81	Mwapasa 2004[94]	Blantyre, Malawi	2000-2002	80·2[147]	0	0	85·3[147]	0	0	3	0·0[152]	0 (Ndirande)	0 (1999-2001)	3	550[152]

	IPTp study Author, Publication Year	Study site, country	Time period study	*Pfdhps* A437G %	Distance in km (location match)	Years difference (study period match)	*Pfdhps* K540E %	Distance in km (location match)	Years difference (study period match)	Score*	*Pfdhps* A581G %	Distance in km (location match)	Years difference (study period match)	Score*	Sample size (N) of marker study
82	Namusoke 2010[95]	Kampala, Uganda	2004-2005	93·5[147]	0	0	95·1[147]	0	0	3	0·0[177]	~200 (Tororo)	0 (2003-2006)	3	55[177]§
83	Ndeserua 2015[96]	Rufiji, Tanzania	2012-2012	75·0[178]	0	-1 (2010-2011)	76·3[179]	0	-1 (2010-2011)	3	0·0[179]	0	-1 (2010-2011)	3	96-97[178,179]
84	Nduka 2011[4]	Umuahia, Afikpo, Okigwe, Nigeria	2009-2009	84·2[138]	~130 (Enugu)	+1 (2010)	0·0[138]	~130 (Enugu)	+1 (2010)	3	47·4[138]	~130 (Enugu)	+1 (2010)		38[138]
85	Ndyomugyenyi 2011[97]	Kabale district, Uganda	2004-2007	100·0[180]	~70 (Bufundi)	0 (2005)	100·0[180]	~70 (Bufundi)	0 (2005)	3	45·0[180]	~70 (Bufundi)	0 (2005)	3	60[180]
86	Nganda 2004[98]	Kibaha, Tanzania	2003-2003	19·8[181]	~20 (Mlandizi)	-1 (2002)	23·6[181]	~20 (Mlandizi)	-1 (2002)	3	0·0[181]	~20 (Mlandizi)	-1 (2002)	3	106[181]
87	Njagi 2002[99]	Bondo, Kenya	1997-1999	42·8[182]	~60 (Kisumu)	0 (1996-2000)	31·1[182]	~60 (Kisumu)	0 (1996-2000)	3	0·0[182]	~60 (Kisumu)	0 (1996-2000)	3	180[182]
88	Obi 2022[100]	Asaba, Nigeria	2021-2021	91·1	0	0	1·5	0	0	3	25·0	0	0		model[6]
89	Oduro 2010[101]	Navrongo, Ghana	2006-2007	53·8[160]	0	0 (2007-2008)	0·0[160]	0	0 (2007-2008)	3	0·0[160]	0	0 (2007-2008)		39[160]
90	Olliaro 2008[102]	Mlomp, Senegal	2000-2007	39·3[147]	0	0	0·03[147]	0	0	3	0·0[183]	~410 (Thies)	0 (2003 & 2008)		108[174]
91	Olorunda 2013[103]	Ibadan, Nigeria	2010-2010	92·4[138]	0 (Ibadan)	-2 (2007-2008)	1·0[138]	0 (Ibadan)	-2 (2007-2008)	3	2·5[138]	0 (Ibadan)	-2 (2007-2008)		198[138]
92	Onoja 2021[104]	Port Harcourt, Nigeria	2018-2018	94·3	0	0	2·7	0	0	3	24·7	0	0		model[6]
93	Onyebuchi 2014[105]	Abakaliki, Nigeria	2012-2012	84·2[138]	~70 (Enugu)	-2 (2010)	0·0[138]	~70 (Enugu)	-2 (2010)	3	47·4[138]	~70 (Enugu)	-2 (2010)		38[138]
94	Orobaton 2016[106]	Sokoto State, Nigeria	2014-2015	78·0	0	0	1·9	0	0	3	9·6	0	0		model[6]
95	Ouma 2012[107]	Nyanza, Kenya	2008-2009	100	0 (Siaya, Bondo)	+2 (2010-2013)	99·5	0 (Siaya, Bondo)	+2 (2010-2013)	3	0·5	0 (Siaya, Bondo)	+2 (2010-2013)	3	203[184]
96	Oweisi 2018[108]	Yenagoa, Nigeria	2016-2016	96·8[138]	~220 (Benin City)	-1 (2014-2015)	0·0	~220 (Benin City)	-1 (2014-2015)	3	0·0	~220 (Benin City)	-1 (2014-2015)		94[138]
97	Parise 1998[109]	Kisumu, Kenya	1994-1996	42·8[182]	0 (Kisumu)	0 (1996-2000)	31·1[182]	0 (Kisumu)	0 (1996-2000)	3	0·0[182]	0 (Kisumu)	0 (1996-2000)	3	180[182]
98	Ramharter 2007[110]	Lambarene, Libreville, Gabon	2005-2006	57·9[185]	0 (Lambarene)	0 (2005-2007)	3·3[185]	0 (Lambarene)	0 (2005-2007)	3	0·0[185]	0 (Lambarene)	0 (2005-2007)		121[185]
99	Rogawski 2012[111]	Blantyre	1997-2006	80·2[147]	0	0	85·3[147]	0	0	3	0·0[152]	0 (Ndirande)	0 (1999-2001)	3	550[152]
100	Rogerson 2000[112]	Blantyre, Malawi	1997-1999	63·6[147]	0	0	74·0[147]	0	0	3	0·0[152]	0 (Ndirande)	0 (1999-2001)	3	550[152]
101	Shulman 1999[113]	Kilifi, Kenya	1996-1997	45·9	0	0	28·7	0	0	3	0·8	0	0	3	model[6]
102	Sirima 2006[114]	Koupela district, Burkina Faso	2004-2004	48·1[147]	0	0	0·1[147]	0	0	3	0·0[40]	~120 (Ziniare)	+6 (2010-2011)		273[40]
103	Stephens 2017[115]	Madina, Ghana	2008-2009	89·6	0	0	1·5	0	0	3	0·7	0	0		model[6]
104	Suleiman 2003[116]	Wad Medani, Sudan	1999-2001	13·3[186]	~190 (Khartoum)	-2 (1996-1997)	0·0[186]	~190 (Khartoum)	-2 (1996-1997)	3	0·0[186]	~190 (Khartoum)	-2 (1996-1997)	3	45[186]

	IPTp study Author, Publication Year	Study site, country	Time period study	*Pfdhps* A437G %	Distance in km (location match)	Years difference (study period match)	*Pfdhps* K540E %	Distance in km (location match)	Years difference (study period match)	Score*	*Pfdhps* A581G %	Distance in km (location match)	Years difference (study period match)	Score*	Sample size (N) of marker study
105	Tagbor 2015[117]	Basse, The Gambia	2010-2012	66·7[174]	~120 (Tambacound)	0 (2010)	1·1	~350 (Kedougou)	0 (2011)	2	2·7	~120 (Tambacound)	0 (2010)		73-81[174] 188[187]
	Tagbor 2015[117]	Navrongo, Ghana	2010-2012	66·6	0	0	0·6	0	0	3	2·1	0	0		model[6]
	Tagbor 2015[117]	Ziniare, Burkina Faso	2010-2012	34·2[153]	~120 (Nanoro)	0 (2012)	0·0	~120 (Nanoro)	0 (2012)	3	0·0[160]	~210 (Navrongo)	-2 (2007-2008)		231[153], 39[160]
	Tagbor 2015[117]	Bamako, Kita, San, Mali	2010-2012	27·7[157]	0	0 (2009-2010)	0·0	0	0 (2009-2010)	3	0·0	0	0 (2009-2010)		130[157]
106	Tetteh-Ashong 2005[118]	Chikwawa, Malawi	2005-2005	94·1[147]	0	0	94·8[147]	0	0	3	0·0[175]	~70 (Chileka)	0 (2003-2005)	3	95[175]
107	Tonga 2013[119]	Sanaga-Maritime, Cameroon	2011-2012	76·5[188]	~180 (Yaounde)	0 (2010-2011)	0·0[188]	~180 (Yaounde)	0 (2010-2011)	3	5·9[188]	~180 (Yaounde)	0 (2010-2011)		51[188]
108	Tongo 2011[120]	Ibadan, Nigeria	2007-2008	92·4[138]	0 (Ibadan)	0 (2007-2008)	1·0[138]	0 (Ibadan)	0 (2007-2008)	3	2·5[138]	0 (Ibadan)	0 (2007-2008)		198[138]
109	Toure 2014[121]	Abidjan and Comoe districts, Cote d'Ivoire	2009-2010	52·1[189]	0 (Abidjan)	-1 (2008)	0·9[189]	0 (Abidjan)	-1 (2008)	3	0·9[189]	0 (Abidjan)	-1 (2008)		94[189]
110	Toure 2019[122]	Kankan, Guinea	2017-2017	72·0	0	0	4·2	0	0	3	1·0	0	0		model[6]
	Toure 2019[122]	Forecariah, Guinea	2017-2017	77·8	0	0	6·1	0	0	3	0·5	0	0		model[6]
	Toure 2019[122]	Nzerekoro, Guinea	2017-2017	88·1	0	0	6·3	0	0	3	0·8	0	0		model[6]
	Toure 2019[122]	Gueckedou, Guinea	2017-2017	82·8[190]	~200 (Kenema)	-1 (2016)	23·4	~200 (Kenema)	-1 (2016)	3	0·0[162]	~650 (Conakry)	-1 (2013-2016)		64[190] 13[162]
111	Tshiongo 2024[123]	Kinshasa, DRC	2018-2018	97·9	0 (Kinshasa)	-3 (2014-2015)	13·8	0 (Kinshasa)	-3 (2014-2015)	2	6·7	0 (Kinshasa)	-3 (2014-2015)		326[167]
112	Tutu 2011[124]	Offinso district, Ghana	2005-2007	77·6[191]	~60 (Sunyani)	0 (2005-2008)	0·0[191]	~60 (Sunyani)	0 (2005-2008)	3	0·0[160]	~60 (Sunyani)	0 (2007-2008)		85[191] 49[160]
113	Umemmuo 2020[125]	Abuja, Nigeria	2017-2017	91·7[162]	0	-2 (2013-2016)	11·1	0	-2 (2013-2016)	3	13·9	0	-2 (2013-2016)		36[162]
114	Valea 2010[126]	Hounde, Burkina Faso	2006-2008	66·7[191]	~266 (Wa)	0 (2005-2006)	0·0	~266 (Wa)	0 (2005-2006)	3	0·0[42]	~300 (Ziniare)	+1 (2009-2010)		39[191] 130[42]
115	van Eijk 2004[127]	Kisumu, Kenya	1999-2000	42·8[182]	0 (Kisumu)	0 (1996-2000)	31·1[182]	0 (Kisumu)	0 (1996-2000)	3	0·0[182]	0 (Kisumu)	0 (1996-2000)	3	180[182]
116	Van Spronsen 2012[128]	Gushegu, Ghana	2010-2010	73·0[147]	0	0	0·7[147]	0	0	3	0·0[160]	~ 200 (Navrongo)	-2 (2008)		39[160]
117	Vanga-Bosson 2011[129]	Cote d'Ivoire	2008-2008	52·1[189]	0 (Abidjan)	0 (2008)	0·9[189]	0 (Abidjan)	0 (2008)	3	0·9[189]	0 (Abidjan)	0 (2008)		94[189]
118	Verhoeff 1998[130]	Chikwawa, Malawi	1993-1994	67·7	0	0	38·7	0	0	3	1·5	0	0	3	model[6]
119	Vincenz 2022[131]	Bandiagara, Bamako Mali	2011-2019	92·8[154]	0	0 (2018	0·7	0	0 (2018	3	0·0	0	0 (2018		801[154]
120	Waltmann 2022[132]	Machinga, Malawi	2017-2018	100·0[55]	0	-2 (2015)	100	0	-2 (2015)	3	2·4	0	-2 (2015)	3	82[55]
121	Wolf 2021[133]	Atlantique Department, Benin	2020-2020	100·0[156]	~72 (Klouékanmey)	-3 (2017)	1·5	~72 (Klouékanmey)	-3 (2017)	2	4·4	~72 (Klouékanmey)	-3 (2017)		68[156]

	IPTp study Author, Publication Year	Study site, country	Time period study	*Pfdhps* A437G %	Distance in km (location match)	Years difference (study period match)	*Pfdhps* K540E %	Distance in km (location match)	Years difference (study period match)	Sco re*	*Pfdhps* A581G %	Distance in km (location match)	Years difference (study period match)	Score*	Sample size (N) of marker study
122	Yussuf 2010[134]	Lindi, Tanzania	2009-2010	79·7[147]	0	0	72·7[179]	~150 (Nachingwea)	0 (2010-2011)	3	0·0[179]	~150 (Nachingwea)	0 (2010-2011)	3	88[179]

* Score for Pfdhps K540E in west and central Africa and for both Pfdhps K540E and A581G in east and southern Africa. The score (maximum 3 points) was determined as follows: 1 point if the geographical distance between clinical and molecular marker studies was <300 km; 1 point if the time difference between studies was ≤3 years; and 1 point if the sample size for molecular marker assessment was ≥30 specimens.
† Mean of two sites: Rukara & Mashesa for Rutsuhuru (Likwela *et al*. 2012)
‡ Placebo arm
§ Non-users of cotrimoxazole

Matching: The following order of preference was used to match resistance with clinical data: 1) resistance data provided in the clinical study reports or by the authors of these reports for that location and time of study, where data from individuals with a recent history of SP intake were excluded; 2) estimates from continuous surface maps from WWARN's geospatial models for *Pfdhps*-A437G and *Pfdhps*-K540E;[147] and 3) for *Pfdhps*-A581G. For studies after 2012, data were used from existing population prevalence maps of *Pfdhps* (Table S2).[192-195]
If contemporaneous molecular data were not available, but the local prevalence of a molecular marker was 0% in studies conducted >2 years after the clinical study, a value of 0% was assumed (e.g., Mali). If a high *Pfdhps*-A581G (>15%) was encountered, the *Pfdhps*-K540E prevalence of the same source study was used. If a high *Pfdhps*-A581G was encountered in the absence of *Pfdhps*-K540E, we assumed the presence *Pfdhps*-A581G occurred in isolation of *Pfdhps*-K540E and did not represent a sextuple mutation. The *Pfdhps*-A581G was therefore scored a 0% (e.g., Esu *et al*. 2018). For sites with sub-optimal matches using source data, modelled data was used by location and year of study using prediction maps (Flegg *et al*. 2022).[6]

Table S3: Adjusted risk ratio and mean difference for several outcomes associated with a difference in IPTp-SP dose by resistance strata in each region, sub-Saharan Africa, 1993-2021

	West and central Africa						East and southern Africa								
	Very low resistance dhps Lys540Glu <4%			Low resistance dhps Lys540Glu ≥4%			Moderate resistance dhps Lys540Glu < 60% & Ala581Gly <5%			High resistance dhps Lys540Glu ≥60% & Ala581Gly <5%			Very high resistance dhps Lys540Glu ≥60% & Ala581Gly ≥5%		
**Dose comparison**	**aRR, 95% CI**	**Studies, n**	**Women, n**	**aRR, 95% CI**	**Studies, n**	**Women, n**	**aRR, 95% CI**	**Studies, n**	**Women, n**	**aRR, 95% CI**	**Studies, n**	**Women, n**	**aRR, 95% CI**	**Studies, n**	**Women, n**
**Binary outcomes**															
**Any malaria infection**															
3 versus 2	0·71, 0·65-0·78	22	3144	0·83, 0·72-0·95	10	1125	0·63, 0·57-0·69	3	731	0·89, 0·82-0·96	22	3184	0·93, 0·85-1·01	9	945
4 versus 3	0·78, 0·73-0·84	7	1313	NA	0	NA		0		0·93, 0·89-0·98	11	1313	0·96, 0·90-1·01	5	387
5 versus 4	0·94, 0·92-0·95	2	105	NA	0	NA		0		0·97, 0·95-0·99	6	776	0·98, 0·96-1·00	4	398
**Maternal peripheral parasitaemia**															
3 versus 2	0·73, 0·66-0·79	14	2259	0·81, 0·69-0·94	8	787	0·53, 0·38-0·69	3	797	0·88, 0·80-0·95	20	3281	0·96, 0·86-1·06	5	450
4 versus 3	0·78, 0·72-0·83	7	1394		0			0		0·92, 0·86-0·97	10	1283	0·97, 0·90-1·04	5	364
5 versus 4	0·93, 0·91-0·95	2	105		0			0		0·96, 0·93-0·99	5	705	0·99, 0·95-1·02	4	366
**Placental parasitaemia**															
3 versus 2	0·71, 0·64-0·79	15	1781	0·85, 0·72-0·97	9	847	0·63, 0·58-0·69	3	731	0·83, 0·75-0·91	13	2366	0·91, 0·78-1·05	7	668
4 versus 3	0·79, 0·73-0·84	3	498		0			0		0·90, 0·85-0·95	6	641	0·95, 0·86-1·03	4	231
5 versus 4		0			0			0		0·96, 0·94-0·98	1	26	0·98, 0·94-1·01	4	355
**Low birth weight**															
3 versus 2	0·58, 0·48-0·68	24	4245	0·56, 0·44-0·68	10	1144	0·75, 0·52-0·98	2	687	0·73, 0·69-0·78	30	8347	0·75, 0·63-0·87	6	746
4 versus 3	0·58, 0·48-0·68	7	1161	0·69, 0·60-0·79	1	81		0		0·77, 0·72-0·81	19	6907	0·77, 0·66-0·88	5	402
5 versus 4	0·84, 0·79-0·89	3	149		0			0		0·88, 0·85-0·90	10	3574	0·88, 0·82-0·94	4	405
**Preterm delivery**															
3 versus 2	0·48, 0·26-0·70	13	1706	0·70, 0·49-0·91	7	866	0·66, 0·22-1·10	1	341	0·65, 0·53-0·77	15	2899	0·54, 0·41-0·68	5	700
4 versus 3	0·42, 0·20-0·65	6	1026		0			0		0·68, 0·57-0·80	7	773	0·54, 0·41-0·68	5	419
5 versus 4	0·83, 0·74-0·93	1	41		0			0		0·82, 0·75-0·89	2	190	0·74, 0·66-0·83	4	420
**Maternal anaemia in 3rd trimester or at delivery (Haemoglobin < 11 g/dl)**															
3 versus 2	0·93, 0·90-0·96	14	1879	0·83, 0·66-1·00	3	590	0·95, 0·90-1·01	2	702	0·95, 0·93-0·98	17	3170	0·95, 0·92-0·98	5	697
4 versus 3	0·93, 0·90-0·96	6	1078					0		0·97, 0·95-0·99	8	1440	0·95, 0·92-0·98	3	267
5 versus 4	0·96, 0·95-0·98	2	106					0		0·97, 0·95-0·99	3	910	0·96, 0·94-0·98	2	116
**Continuous outcomes**															
	**Mean difference, 95% CI**	**Studies, n**	**Women, n**	**Mean difference, 95% CI**	**Studies, n**	**Women, n**	**Mean difference, 95% CI**	**Studies, n**	**Women, n**	**Mean difference, 95% CI**	**Studies, n**	**Women, n**	**Mean difference, 95% CI**	**Studies, n**	**Women, n**
**Birth weight (grams)**															
3 versus 2	120, 84-157	20	3184	145, 76-213	8	692	161, 2-319	2	687	74, 55-93	27	7889	71, 30-112	8	799
4 versus 3	120, 83-156	9	1191	93, 49-137	1	81		0		66, 49-83	18	6829	67, 29-105	5	391
5 versus 4	49, 34-64	4	161		0			0		35, 26-44	11	3600	34, 15-54	4	405
**Gestational age (weeks)**															
3 versus 2	0·27, 0·08-0·47	10	1311	0·09, -0·17-0·35	5	416		0		0·37, 0·18-0·56	13	6234	0·59, 0·34-0·84	6	713
4 versus 3	0·30, 0·09-0·52	6	1032		0			0		0·33, 0·16-0·50	8	5305	0·56, 0·33-0·80	5	404
5 versus 4	0·07, 0·02-0·12	1	41		0			0		0·19, 0·09-0·29	6	2578	0·29, 0·17-0·42	4	417
Maternal haemoglobin in late third trimester or at delivery (g/dl)															

3 versus 2	0·15, 0·11-0·20	7	987	0·19, 0·19-0·19	1	140	0·15, 0·08-0·21	1	362	0·09, 0·03-0·14	13	2216	0·08, 0·01-0·15	5	451
4 versus 3	0·13, 0·09-0·17	2	316		0			0		0·07, 0·03-0·11	8	1419	0·07, 0·01-0·13	5	384
5 versus 4	0·11, 0·07-0·14	2	106		0			0		0·06, 0·02-0·10	4	978	0·06, 0·01-0·11	4	413

aRR, adjusted risk ratio. CI, confidence interval. N, number. *Dhfr,* dihydrofolate reductase gene. *Dhps,* dihydropteroate synthase gene. IPTp-SP, intermittent preventive treatment in pregnancy with sulfadoxine–pyrimethamine. NA, not applicable. Any malaria infection: parasitaemia in peripheral maternal blood or placental blood detected by any malaria test.

The multivariable meta-regression analysis was adjusted for: a) the *Pf*$PR_{2\text{-}10}$: *P. falciparum* parasite prevalence in children aged 2-10 years as obtained for the year of study and study locations from the Malaria Atlas Project (https://malariaatlas.org), b) the prevalence of HIV-infected women in the study population, as reported by the study, or as estimated by UNAIDS for the country population, c) the percentage of primigravidae or primigravidae and secundigravidae combined in the study population as reported by the study, d) use of insecticide treated nets.

Table S4. Population average adjusted risk ratio and mean difference for several outcomes associated with a difference in IPTp-SP dose by resistance strata in each region, 1997-2021

	Western and central Africa		East and southern Africa		
**Dose comparison**	**Very low resistance dhps Lys540Glu <4% aRR, 95% CI**	**Low resistance dhps Lys540Glu ≥4% aRR, 95% CI**	**Moderate resistance dhps Lys540Glu < 60% & Ala581Gly <5% aRR, 95% CI**	**High resistance dhps Lys540Glu ≥60% & Ala581Gly <5% aRR, 95% CI**	**Very high resistance dhps Lys540Glu ≥60% & Ala581Gly ≥5% aRR, 95% CI**
	**Any malaria**				
**3 vs. 2**	0·71, 0·65-0·78	0·83, 0·72-0·96	0·63, 0·58-0·69	0·89, 0·82-0·96	0·93, 0·86-1·02
**4 vs. 3**	0·78, 0·73-0·84	No data	No data	0·93, 0·89-0·98	0·96, 0·91-1·01
**5 vs. 4**	0·92, 0·91-0·94	No data	No data	0·97, 0·95-0·99	0·98, 0·96-1·00
	**Maternal malaria**				
**3 vs. 2**	0·73, 0·66-0·80	0·84, 0·74-0·96	0·55, 0·42-0·73	0·88, 0·81-0·96	0·96, 0·86-1·06
**4 vs. 3**	0·79, 0·73-0·84	No data	No data	0·92, 0·86-0·97	0·97, 0·90-1·04
**5 vs. 4**	0·93, 0·91-0·95	No data	No data	0·96, 0·93-0·99	0·99, 0·95-1·02
	**Placental malaria**				
**3 vs. 2**	0·71, 0·64-0·79	0·85, 0·73-0·98	0·63, 0·58-0·69	0·83, 0·75-0·91	0·91, 0·78-1·06
**4 vs. 3**	0·79, 0·73-0·85	No data	No data	0·90, 0·85-0·95	0·95, 0·87-1·03
**5 vs. 4**	No data	No data	No data	0·96, 0·94-0·98	0·98, 0·94-1·01
	**Low birth weight**				
**3 vs. 2**	0·58, 0·49-0·69	0·56, 0·45-0·70	0·75, 0·55-1·02	0·73, 0·69-0·79	0·75, 0·64-0·88
**4 vs. 3**	0·58, 0·49-0·69	No data	No data	0·78, 0·72-0·81	0·77, 0·66-0·89
**5 vs. 4**	insufficient data	No data	No data	0·88, 0·85-0·90	0·88, 0·82-0·95
	**Preterm**				
**3 vs. 2**	0·48, 0·30-0·76	0·70, 0·52-0·94	0·66, 0·34-1·29	0·65, 0·54-0·79	0·54, 0·42-0·69
**4 vs. 3**	0·42, 0·25-0·73	No data	No data	0·68, 0·58-0·81	0·54, 0·42-0·69
**5 vs. 4**	0·83, 0·74-0·93	No data	No data	0·82, 0·75-0·89	0·74, 0·66-0·84
	**Maternal anaemia**				
**3 vs. 2**	0·93, 0·90-0·96	0·83, 0·68-1·02	0·95, 0·90-1·01	0·96, 0·93-0·98	0·95, 0·92-0·98
**4 vs. 3**	0·93, 0·90-0·96	No data	No data	0·97, 0·95-0·99	0·95, 0·93-0·98
**5 vs. 4**	0·96, 0·95-0·98	No data	No data	0·97, 0·95-0·99	0·96, 0·94-0·98
	**Adjusted mean difference, 95% CI**	**Adjusted mean difference, 95% CI**	**Adjusted mean difference, 95% CI**	**Adjusted mean difference, 95% CI**	**Adjusted mean difference, 95% CI**
	**Birth weight**				
**3 vs. 2**	120, 84-157	145, 76-213	161, 2-319	74, 55-93	71, 30-112
**4 vs. 3**	120, 83-157	No data	No data	66, 49-83	67, 29-105
**5 vs. 4**	49, 34-64	No data	No data	35, 26-44	34, 15-54
	**Gestational age**				
**3 vs. 2**	0·27, 0·08-0·47	0·09, -0·17-0·35	No data	0·37, 0·18-0·56	0·59, 0·34-0·84
**4 vs. 3**	0·30, 0·09-0·52	No data	No data	0·33, 0·16-0·50	0·56, 0·33-0·80
**5 vs. 4**	0·07, 0·02-0·12	No data	No data	0·19, 0·09-0·29	0·29, 0·17-0·42
	**Haemoglobin**				
**3 vs. 2**	0·15, 0·11-0·20	0·19, 0·19-0·91	0·15, 0·08-0·21	0·09, 0·03-0·14	0·08, 0·01-0·15
**4 vs. 3**	0·13, 0·09-0·17	No data	No data	0·07, 0·03-0·11	0·07, 0·01-0·13
**5 vs. 4**	0·11, 0·07-0·14	No data	No data	0·06, 0·02-0·10	0·06, 0·01-0·11

aRR, adjusted risk ratio. CI, confidence interval. N, number. *Dhfr,* dihydrofolate reductase gene. *Dhps,* dihydropteroate synthase gene. IPTp-SP, intermittent preventive treatment in pregnancy with sulfadoxine–pyrimethamine. Any malaria infection: parasitaemia in peripheral maternal blood or placental blood detected by any malaria test.

The multivariable meta-regression analysis was adjusted for: a) the *Pf*$PR_{2\text{-}10}$: *P. falciparum* parasite prevalence in children aged 2-10 years as obtained for the year of study and study locations from the Malaria Atlas Project (https://malariaatlas.org), b) the prevalence of HIV-infected women in the study population, as reported by the study, or as estimated by UNAIDS for the country population, c) the percentage of primigravidae or primigravidae and secundigravidae combined in the study population as reported by the study, d) use of insecticide treated nets.

Table S5. Adjusted risk ratio for any malaria and low birth weight associated with a difference in IPTp-SP dose by resistance strata in each region by gravidity, 1997-2021

	**West and central Africa**						**East and southern Africa**								
	**Very low resistance dhps Lys540Glu <4%**			**Low resistance dhps Lys540Glu ≥4%**			**Moderate resistance dhps Lys540Glu < 60% & Ala581Gly <5%**			**High resistance dhps Lys540Glu ≥60% & Ala581Gly <5%**			**Very high resistance dhps Lys540Glu ≥60% & Ala581Gly ≥5%**		
**Dose comparison**	**aRR, 95% CI**	**N studies**	**N women**	**aRR, 95% CI**	**N studies**	**N women**	**aRR, 95% CI**	**N studies**	**N women**	**aRR, 95% CI**	**N studies**	**N women**	**aRR, 95% CI**	**N studies**	**N women**
Any malaria infection paucigravidae §															
**3 vs 2**	0·70, 0·61-0·79	11	1007	0·88, 0·68-1·09	3	141	0·61, 0·38-0·83	2	674	0·82, 0·69-0·96	13	1152	0·94, 0·87-1·02	4	275
**4 vs 3**	0·78, 0·71-0·85	4	359		0			0		0·90, 0·81-0·98	5	181	0·96, 0·91-1·01	4	144
**5 vs 4**	0·94, 0·92-0·96	1	44		0			0		0·96, 0·93-0·99	1	21	0·99, 0·97-1·00	3	100
Any malaria multigravidae															
**3 vs 2**	0·81, 0·75-0·89	11	1797	0·87, 0·78-0·96	3	230		0		0·87, 0·77-0·97	12	1250	0·95, 0·84-1·07	4	369
**4 vs 3**	0·87, 0·82-0·92	5	1100		0			0		0·92, 0·86-0·98	4	227	0·97, 0·88-1·05	4	166
**5 vs 4**	0·96, 0·94-0·98	1	26		0			0		0·97, 0·94-0·99	1	32	0·99, 0·95-1·02	3	242
Low birth weight paucigravidae															
**3 vs 2**	0·62, 0·51-0·73	13	1019	0·49, 0·35-0·63	3	140	0·69, 0·57-0·80	2	687	0·76, 0·71-0·81	21	3084	0·80, 0·67-0·94	4	257
**4 vs 3**	0·63, 0·52-0·74	5	395		0			0		0·79, 0·75-0·83	13	2438	0·81, 0·68-0·94	3	130
**5 vs 4**	0·87, 0·82-0·92	1	44					0		0·91, 0·88-0·93	5	1111	0·91, 0·84-0·97	3	101
Low birth weight multigravidae															
**3 vs 2**	0·75, 0·65-0·84	12	3641	0·51, 0·38-0·64	3	639		0		0·73, 0·72-0·75	21	9128	0·74, 0·65-0·82	5	916
**4 vs 3**	0·74, 0·65-0·83	4	1433		0			0		0·76, 0·74-0·77	12	7064	0·75, 0·67-0·83	4	350
**5 vs 4**	0·91, 0·87-0·94	2	208		0			0		0·87, 0·86-0·88	6	3694	0·86, 0·82-0·91	3	365

aRR, adjusted risk ratio. CI, confidence interval. N, number. *Dhfr,* dihydrofolate reductase gene. *Dhps,* dihydropteroate synthase gene. IPTp-SP, intermittent preventive treatment in pregnancy with sulfadoxine–pyrimethamine. Any malaria infection: parasitaemia in peripheral maternal blood or placental blood detected by any malaria test.

The multivariable meta-regression analysis was adjusted for: a) the *Pf*$PR_{2\text{-}10}$: *P. falciparum* parasite prevalence in children aged 2-10 years as obtained for the year of study and study locations from the Malaria Atlas Project (https://malariaatlas.org), b) the prevalence of HIV-infected women in the study population, as reported by the study, or as estimated by UNAIDS for the country population, c) use of insecticide treated nets. HIV was not included in the models for paucigravidae because of non-convergence.

Table S6: Adjusted risk ratio and mean difference for outcomes associated with a difference in IPTp-SP dose by resistance strata in each region, when including study-quality-assessment as co-variate in the model, sub-Saharan Africa, 1997-2021

	West and central Africa						East and southern Africa								
	Very low resistance dhps Lys540Glu <4%			Low resistance dhps Lys540Glu ≥4%			Moderate resistance dhps Lys540Glu < 60% & Ala581Gly <5%			High resistance dhps Lys540Glu ≥60% & Ala581Gly <5%			Very high resistance dhps Lys540Glu ≥60% & Ala581Gly ≥5%		
**Dose comparison**	**aRR, 95% CI**	**N studies**	**N women**	**aRR, 95% CI**	**N studies**	**N women**	**aRR, 95% CI**	**N studies**	**N women**	**aRR, 95% CI**	**N studies**	**N women**	**aRR, 95% CI**	**N studies**	**N women**
**Binary outcomes**															
Any malaria infection															
**3 vs 2**	0·71, 0·65-0·78	22	3144	0·83, 0·72-0·95	10	1125	0·63, 0·57-0·69	3	731	0·89, 0·82-0·96	22	3184	0·93, 0·85-1·01	9	945
**4 vs 3**	0·78, 0·73-0·84	7	1313		0			0		0·93, 0·89-0·98	11	1313	0·96, 0·90-1·01	5	387
**5 vs 4**	0·94, 0·92-0·95	2	105		0			0		0·97, 0·95-0·99	6	776	0·98, 0·96-1·00	4	398
Maternal peripheral parasitaemia															
**3 vs 2**	0·73, 0·66-0·80	14	2259	0·81, 0·68-0·93	8	787	0·53, 0·38-0·69	3	797	0·87, 0·79-0·95	20	3281	0·95, 0·85-1·05	5	450
**4 vs 3**	0·78, 0·73-0·84	7	1394		0			0		0·91, 0·86-0·97	10	1283	0·96, 0·89-1·04	5	364
**5 vs 4**	0·93, 0·91-0·95	2	105		0			0		0·96, 0·93-0·99	5	705	0·98, 0·95-1·02	4	366
Placental parasitaemia															
**3 vs 2**	0·72, 0·64-0·79	15	1781	0·85, 0·72-0·97	9	847	0·63, 0·58-0·69	3	731	0·83, 0·75-0·91	13	2366	0·91, 0·78-1·05	7	668
**4 vs 3**	0·79, 0·73-0·85	3	498					0		0·90, 0·85-0·95	6	641	0·95, 0·86-1·03	4	231
**5 vs 4**		0						0		0·96, 0·94-0·98	1	26	0·98, 0·94-1·01	4	355
Low birth weight															
**3 vs 2**	0·58, 0·48-0·68	24	4245	0·56, 0·44-0·69	10	1144	0·74, 0·49-0·99	2	687	0·74, 0·69-0·79	30	8347	0·76, 0·64-0·88	6	746
**4 vs 3**	0·58, 0·48-0·68	7	1161	0·69, 0·60-0·79	1	81		0		0·77, 0·72-0·81	19	6907	0·78, 0·67-0·89	5	402
**5 vs 4**	0·84, 0·79-0·89	3	149		0			0		0·88, 0·86-0·90	10	3574	0·89, 0·83-0·95	4	405
Preterm delivery															
**3 vs 2**	0·47, 0·25-0·70	13	1706	0·69, 0·49-0·89	7	866	0·67, 0·22-1·12	1	341	0·66, 0·53-0·78	15	2899	0·54, 0·41-0·68	5	700
**4 vs 3**	0·42, 0·19-0·65	6	1026		0			0		0·69, 0·57-0·80	7	773	0·54, 0·41-0·67	5	419
**5 vs 4**	0·83, 0·73-0·93	1	41		0			0		0·82, 0·75-0·89	2	190	0·75, 0·66-0·83	4	420
Maternal anaemia in 3rd trimester or at delivery (Haemoglobin < 11 g/dl)															
**3 vs 2**	0·89, 0·84-0·94	14	1879	0·86, 0·69-1·03	3	590	0·95, 0·90-1·01	2	702	0·96, 0·93-0·98	17	3170	0·95, 0·92-0·98	5	697
**4 vs 3**	0·89, 0·83-0·94	6	1078					0		0·96, 0·94-0·98	8	1440	0·95, 0·92-0·98	3	267
**5 vs 4**	0·94, 0·91-0·97	2	106					0		0·97, 0·95-0·99	3	910	0·96, 0·94-0·99	2	116
**Continuous outcomes**															
	**Mean difference, 95% CI**	**N studies**	**N women**	**Mean difference, 95% CI**	**N studies**	**N women**	**Mean difference, 95% CI**	**N studies**	**N women**	**Mean difference, 95% CI**	**N studies**	**N women**	**Mean difference, 95% CI**	**N studies**	**N women**
Birthweight (grams)															
**3 vs 2**	120, 84-157	20	3184	145, 76-213	8	692	161, 2-319	2	687	74, 55-93	27	7889	71, 30-112	8	799
**4 vs 3**	120, 83-156	9	1191	93, 49-137	1	81		0		66, 49-83	18	6829	67, 29-105	5	391
**5 vs 4**	49, 34-64	4	161		0			0		35, 26-44	11	3600	34, 15-54	4	405
Gestational age (weeks)															

**3 vs 2**	0·27, 0·08-0·47	10	1311	0·09, -0·17-0·35	5	416		0		0·37, 0·18-0·56	13	6234	0·59, 0·34-0·84	6	713
**4 vs 3**	0·30, 0·09-0·52	6	1032		0			0		0·33, 0·16-0·50	8	5305	0·56, 0·33-0·80	5	404
**5 vs 4**	0·07, 0·02-0·12	1	41		0			0		0·19, 0·09-0·29	6	2578	0·29, 0·17-0·42	4	417
Maternal haemoglobin in late third trimester or at delivery (g/dl)															
**3 vs 2**	0·15, 0·11-0·20	7	987	0·19, 0·19-0·19	1	140	0·15, 0·08-0·21	1	362	0·10, 0·03-0·17	13	2216	0·06, -0·0-0·12	5	451
**4 vs 3**	0·13, 0·09-0·17	2	316		0			0		0·08, 0·03-0·13	8	1419	0·05, -0·00-0·10	5	384
**5 vs 4**	0·11, 0·07-0·14	2	106		0			0		0·07, 0·02-0·12	4	978	0·04, -0·00-0·09	4	413

aRR, adjusted risk ratio. CI, confidence interval. N, number. *Dhfr,* dihydrofolate reductase gene. *Dhps,* dihydropteroate synthase gene. IPTp-SP, intermittent preventive treatment in pregnancy with sulfadoxine–pyrimethamine. Any malaria infection: parasitaemia in peripheral maternal blood or placental blood detected by any malaria test.

The multivariable meta-regression analysis was adjusted for: a) the *Pf*PR$_{2\text{-}10}$: *P. falciparum* parasite prevalence in children aged 2-10 years as obtained for the year of study and study locations from the Malaria Atlas Project (https://malariaatlas.org), b) the prevalence of HIV-infected women in the study population, as reported by the study, or as estimated by UNAIDS for the country population, c) the percentage of primigravidae or primigravidae and secundigravidae combined in the study population as reported by the study, d) use of insecticide treated nets.

Table 7: Adjusted risk ratio and mean difference for several outcomes associated with a difference in IPTp-SP dose by resistance strata in each region, sub-Saharan Africa, 1997-2021, trials only

	West and central Africa						East and southern Africa								
	Very low resistance dhps Lys540Glu <4%			Low resistance dhps Lys540Glu ≥4%			Moderate resistance dhps Lys540Glu < 60% & Ala581Gly <5%			High resistance dhps Lys540Glu ≥60% & Ala581Gly <5%			Very high resistance dhps Lys540Glu ≥60% & Ala581Gly ≥5%		
**Dose comparison**	**aRR, 95% CI**	**N studies**	**N women**	**aRR, 95% CI**	**N studies**	**N women**	**aRR, 95% CI**	**N studies**	**N women**	**aRR, 95% CI**	**N studies**	**N women**	**aRR, 95% CI**	**N studies**	**N women**
**Binary outcomes**															
Any malaria infection															
**3 vs 2**	0·59, 0·45-0·73	6	1084	0·84, 0·68-0·99	1	184	0·60, 0·51-0·69	2	674	0·88, 0·56-1·21	5	216	0·94, 0·74-1·14	5	294
**4 vs 3**	0·71, 0·60-0·82	2	409		0			0		0·93, 0·72-1·14	5	343	0·96, 0·84-1·09	4	251
**5 vs 4**	0·85, 0·79-0·91	1	35		0			0		0·97, 0·87-1·07	3	150	0·98, 0·93-1·04	4	398
Maternal peripheral parasitaemia															
**3 vs 2**	0·70, 0·54-0·86	6	1107		0		0·45, 0·32-0·58	3	797	0·65, 0·48-0·81	3	65	0·86, 0·71-1·01	3	160
**4 vs 3**	0·78, 0·65-0·91	2	409		0			0		0·75, 0·63-0·88	3	214	0·90, 0·79-1·01	4	228
**5 vs 4**	0·87, 0·80-0·95	1	35		0			0		0·86, 0·80-0·94	1	53	0·95, 0·89-1·01	4	366
Placental parasitaemia	Model not converging														
**3 vs 2**							0·60, 0·51-0·70	2	674	0·70, 0·57-0·84	2	41	0·88, 0·55-1·21	4	271
**4 vs 3**								0		0·82, 0·74-0·91	2	189	0·92, 0·72-1·13	4	231
**5 vs 4**								0		0.93, 0.89-0.96	1	128	0·97, 0·88-1·06	4	355
Low birth weight							Model not converging								
**3 vs 2**	0·56, 0·44-0·71	6	997	0·60, 0·26-1·39	2	235									
**4 vs 3**	0·57, 0·46-0·72	1	142		0										
**5 vs 4**	0·74, 0·65-0·84	3	149		0										
Preterm delivery	Model not converging														
**3 vs 2**							0·90, 0·68-1·12	1	341	0·53, 0·36-0·70	4	157	0·46, 0·28-0·63	3	180
**4 vs 3**								0		0·58, 0·43-0·74	4	323	0·46, 0·30-0·63	4	284
**5 vs 4**								0		0·73, 0·61-0·84	2	190	0·67, 0·55-0·80	4	420
Maternal anaemia in 3rd trimester or at delivery (Haemoglobin < 11 g/dl)							Models not converging								
**3 vs 2**															
**4 vs 3**															
**5 vs 4**															
**Continuous outcomes**															
	**Mean difference, 95% CI**	**N studies**	**N women**	**Mean difference, 95% CI**	**N studies**	**N women**	**Mean difference, 95% CI**	**N studies**	**N women**	**Mean difference, 95% CI**	**N studies**	**N women**	**Mean difference, 95% CI**	**N studies**	**N women**
Birthweight (grams)															
**3 vs 2**	75, 2-148	2	687		0		75, 2-148	2	687	78, 58-98	5	275	86, 26-146	5	238
**4 vs 3**		0			0			0		85, 34-137	5	412	78, 23-133	4	262
**5 vs 4**	114, 60-168	1	38		0			0		49, 20-79	3	213	44, 13-74	4	405
Gestational age (weeks)															

**3 vs 2**	-0·00, -0·22-0·21	3	585		0			0		0·16, 0·09-0·22	2	214	0·65, 0·23-1·08	4	189
**4 vs 3**	-0·00, -0·23-0·23	1	171		0			0		0·13, 0·08-0·19	2	187	0·60, 0·21-0·99	4	269
**5 vs 4**		0			0			0		0·08, 0·05-0·11	2	136	0·34, 0·12-0·56	4	417
Maternal haemoglobin in late third trimester or at delivery (g/dl)															
**3 vs 2**	0·16, 0·05-0·26	4	782		0		0·15, 0·08-0·21	1	362	0·18, -0·02-0·38	3	61	0·11, -0·02-0·24	3	165
**4 vs 3**	0·13, 0·04-0·22	1	142		0			0		0·14, -0·02-0·29	3	222	0·09, -0·02-0·20	4	248
**5 vs 4**	0·13, 0·04-0·22	1	37		0			0		0·14, -0·02-0·29	1	68	0·08, -0·02-0·18	4	413

aRR, adjusted risk ratio. CI, confidence interval. N, number. NA, not applicable Any malaria infection: parasitaemia in peripheral maternal blood or placental blood detected by any malaria test.

The multivariable meta-regression analysis was adjusted for: a) the *Pf*$PR_{2\text{-}10}$: *P. falciparum* parasite prevalence in children aged 2-10 years as obtained for the year of study and study locations from the Malaria Atlas Project (https://malariaatlas.org), b) the prevalence of HIV-infected women in the study population, as reported by the study, or as estimated by UNAIDS for the country population, c) the percentage of primigravidae or primigravidae and secundigravidae combined in the study population as reported by the study, d) use of insecticide treated nets.

Table S8: Adjusted risk ratio and mean difference for several outcomes associated with a difference in IPTp-SP dose by resistance strata in each region, sub-Saharan Africa, 1997-2021, trials and cohort studies only

	West and central Africa						East and southern Africa								
	**Very low resistance dhps Lys540Glu <4%**			**Low resistance dhps Lys540Glu ≥4%**			**Moderate resistance dhps Lys540Glu < 60% & Ala581Gly <5%**			**High resistance dhps Lys540Glu ≥60% & Ala581Gly <5%**			**Very high resistance dhps Lys540Glu ≥60% & Ala581Gly ≥5%**		
**Dose comparison**	**aRR, 95% CI**	**N studies**	**N women**	**aRR, 95% CI**	**N studies**	**N women**	**aRR, 95% CI**	**N studies**	**N women**	**aRR, 95% CI**	**N studies**	**N women**	**aRR, 95% CI**	**N studies**	**N women**
**Binary outcomes**															
Any malaria infection							Model not converging								
**3 vs 2**	0·64, 0·55-0·76	13	2228	0·80, 0·77-0·84	2	269									
**4 vs 3**	0·72, 0·64-0·81	6	1136		0										
**5 vs 4**	0·91, 0·87-0·94	1	35		0										
Maternal peripheral parasitaemia															
**3 vs 2**	0·67, 0·57-0·79	11	1758	0·72, 0·70-0·74	1	81	0·47, 0·36-0·61	3	797	0·80, 0·62-1·03	7	421	0·91, 0·76-1·09	4	195
**4 vs 3**	0·73, 0·64-0·83	6	1217		0			0		0·87, 0·75-1·02	5	335	0·94, 0·83-1·06	4	228
**5 vs 4**	0·90, 0·86-0·94	1	35		0			0		0·93, 0·86-1·01	2	79	0·97, 0·91-1·03	4	366
Placental parasitaemia															
**3 vs 2**	0·58, 0·49-0·69	8	998	0·79, 0·75-0·83	2	257	0·61, 0·53-0·70	3	731	0·87, 0·63-1·21	5	360	0·89, 0·69-1·15	6	557
**4 vs 3**	0·67, 0·59-0·77	3	498					0		0·93, 0·79-1·10	4	312	0·93, 0·80-1·09	4	231
**5 vs 4**		0						0		0·97, 0·91-1·04	1	26	0·97, 0·92-1·03	4	355
Low birth weight															
**3 vs 2**	0·56, 0·40-0·72	15	2526	0·47, 0·40-0·55	3	316	0·62, 0·36-0·88	2	687	0·70, 0·56-0·85	8	743	0·69, 0·51-0·87	5	500
**4 vs 3**	0·54, 0·37-0·70	5	930		0			0		0·75, 0·63-0·88	6	460	0·73, 0·56-0·89	4	272
**5 vs 4**	0·82, 0·74-0·90	1	38		0			0		0·86, 0·79-0·94	2	187	0·85, 0·75-0·95	4	405
Preterm delivery															
**3 vs 2**	0·44, 0·15-0·73	11	1644	0·55, 0·24-0·87	2	266	0·65, 0·19-1·11	1	341	0·53, 0·38-0·68	7	485	0·47, 0·34-0·60	4	460
**4 vs 3**	0·40, 0·10-0·69	5	972		0			0		0·59, 0·45-0·73	5	450	0·48, 0·35-0·61	4	284
**5 vs 4**		0			0			0		0·74, 0·65-0·84	2	190	0·68, 0·59-0·78	4	420
Maternal anaemia in 3rd trimester or at delivery (Haemoglobin < 11 g/dl)															
**3 vs 2**	Not converging						0·93, 0·88-0·98	2	702	0·92, 0·84-0·99	4	218	0·97, 0·91-1·03	2	442
**4 vs 3**								0		0·94, 0·87-0·99	3	214	0·97, 0·92-1·02	2	131
**5 vs 4**								0			0		0·97, 0·93-1·02	2	116
**Continuous outcomes**															
	**Mean difference, 95% CI**	**N studies**	**N women**	**Mean difference, 95% CI**	**N studies**	**N women**	**Mean difference, 95% CI**	**N studies**	**N women**	**Mean difference, 95% CI**	**N studies**	**N women**	**Mean difference, 95% CI**	**N studies**	**N women**
Birthweight (grams)															
**3 vs 2**	138, 69-207	11	1545	49, -39-136	2	130	171, -15-357	2	687	109, 61-157	7	472	80, 28-133	7	552
**4 vs 3**	152, 76-228	5	930		0			0		91, 51-131	6	434	73, 25-121	4	262
**5 vs 4**	51, 26-77	1	38		0			0		52, 29-75	3	213	41, 14-68	4	405

Gestational age (weeks)															
**3 vs 2**	0·18, 0·02-0·35	8	1249		0			0		0·36, 0·00-0·72	3	349	0·67, 0·38-0·97	5	469
**4 vs 3**	0·21, 0·02-0·40	5	978		0			0		0·29, 0·00-0·57	2	187	0·62, 0·35-0·89	4	269
**5 vs 4**		0			0			0		0·17, 0·00-0·35	2	136	0·35, 0·20-0·51	4	417
Maternal haemoglobin in late third trimester or at delivery (g/dl)															
**3 vs 2**	0·15, 0·05-0·25	4	782		0		0·15, 0·08-0·21	1	362	0·16, 0·03-0·30	5	238	0·07, -0·02-0·16	4	196
**4 vs 3**	0·13, 0·05-0·20	1	142		0			0		0·12, 0·02-0·23	4	243	0·06, -0·01-0·14	4	248
**5 vs 4**	0·13, 0·05-0·21	1	37		0			0		0·12, 0·02-0·22	1	68	0·06, -0·01-0·12	4	413

aRR, adjusted risk ratio. CI, confidence interval. N, number. Any malaria infection: parasitaemia in peripheral maternal blood or placental blood detected by any malaria test.
The multivariable meta-regression analysis was adjusted for: a) the *Pf*PR$_{2\text{-}10}$: *P. falciparum* parasite prevalence in children aged 2-10 years as obtained for the year of study and study locations from the Malaria Atlas Project (https://malariaatlas.org), b) the prevalence of HIV-infected women in the study population, as reported by the study, or as estimated by UNAIDS for the country population, c) the percentage of primigravidae or primigravidae and secundigravidae combined in the study population as reported by the study, d) use of insecticide treated nets.

Table S9. AICs for the association between SP doses and pregnancy outcomes

Any malaria			Peripheral malaria			Placental malaria		
Dose transformation*	AIC	Proportion AIC change†	Dose transformation*	AIC	Proportion AIC change†	Dose transformation*	AIC	Proportion AIC change†
T13	2611·734		T11	1899·077		T6	1906·492	
T6	2615·838	0·16	T6	1913·596	0·76	T13	1914·617	0·43
T12	2617·569	0·07	T10	1915·428	0·10	T11	1916·784	0·11
T11	2624·656	0·27	T5	1917·311	0·10	T5	1925·179	0·44
T14	2647·605	0·87	T13	1921·221	0·20	T14	1929·07	0·20
T5	2653·116	0·21	T9	1925·971	0·25	T12	1930·077	0·05
T10	2660·555	0·28	T12	1927·293	0·07	T10	1958·223	1·46
T4	2719·067	2·20	T4	1956·223	1·50	T4	2026·842	3·50
T9	4515·171	66·06	T14	1956·945	0·04	T9	3263·026	60·99
T7	4680·231	3·66	T7	2278·533	16·43	T7	3820·306	17·08
T3	14015·99	199·47	T3	11756·94	415·99	T8	23552·62	516·51
T2			T2			T2		
T1			T8			T1		
T8			T1			T3		

Low birth weight			Preterm delivery			Anaemia <11 g/dl		
Dose transformation*	AIC	Proportion AIC change†	Dose transformation*	AIC	Proportion AIC change†	Dose transformation*	AIC	Proportion AIC change†
T6	2812·468		T7	1696·224		T12	1744·975	
T14	2822·854	0·37	T6	1711·691	0·91	T5	1781·411	2·09
T7	2830·912	0·29	T14	1729·177	1·02	T11	1796·919	0·87
T13	2862·202	1·11	T13	1747·745	1·07	T13	1797·456	0·03
T12	2911·59	1·73	T11	1783·555	2·05	T6	1809·996	0·70
T11	2939·084	0·94	T5	1784·475	0·05	T4	1817·322	0·40
T5	3023·516	2·87	T11	1805·933	1·20	T14	1824·818	0·41
T10	3047·279	0·79	T10	1862·568	3·14	T9	1826·232	0·08
T9	3122·585	2·47	T9	1890·281	1·49	T8	1846·351	1·10
T4	3213·683	2·92	T4	1898·483	0·43	T7	1852·123	0·31
T8	3238·227	0·76	T8	1948·709	2·65	T3	1867·555	0·83

T3	11228·08	246·74	T3	2108·705	8·21	T10	1908·544	2·19
T2			T2			T2		
T1			T1			T1		

Mean birth weight			Mean gestational age at delivery			Mean haemoglobin at delivery or 3rd trimester		
Dose transformation*	AIC	Proportion AIC change†	Dose transformation*	AIC	Proportion AIC change†	Dose transformation*	AIC	Proportion AIC change†
T14	891023·1		T14	-72512·3		T6	-86679·39	
T6	898710·4	0·86	T13	-58794·91	-18·92	T5	-81580·16	-5·88
T13	903217·5	0·50	T6	-56637·17	-3·67	T12	-80428·87	-1·41
T7	903479·3	0·03	T7	-54224·5	-4·26	T13	-80087·12	-0·42
T12	924529·6	2·33	T12	-38536·25	-28·93	T11	-78833·46	-1·57
T5	946770·1	2·41	T5	-19621·74	-49·08	T7	-78202·13	-0·80
T11	950436·8	0·39	T11	-16558·91	-15·61	T14	-75750·88	-3·13
T10	974781·4	2·56	T10	2110·025	-112·74	T10	-75513·73	-0·31
T9	993131·1	1·88	T9	15238·66	622·20	T9	-71565·84	-5·23
T4	1004521	1·15	T4	17666·53	15·93	T4	-65882·88	-7·94
T8	1013876	0·93	T8	28853·82	63·32	T8	-64493·13	-2·11
T3	1017479	0·36	T3	30075·34	4·23	T3	-60627·03	-5·99
T2	1048714	3·07	T2	32646·2	8·55	T2	-46044·81	-24·05
T1	1048729	0·00	T1	46455·39	42·30	T1	-46037·36	-0·02

AIC, Akaike information criterion.

*For type of transformation, see table below. If no AIC was completed, the model did not converge. In red, the transformation that was used for the outcome. For the reasoning, see page 9 of the supplement.

†Proportion AIC change compared to previous model

Table S9b: Transformation of SP dose variable explored

Transformation number	Transformation of x	Transformation number	Transformation of x
T1	$x^{-2} = 1/x^2 = 1/(x*x)$	T8	ACD transformation followed by x^{-2}
T2	$x^{-1} = 1/x$	T9	ACD transformation followed by x^{-1}
T3	$x^{-0.5} = 1/x^{0.5} = 1/\sqrt{x}$	T10	ACD transformation followed by $x^{-0.5}$
T4	ln(x) (natural log of x)	T11	ACD transformation followed by ln(x)
T5	$x^{0.5} = \sqrt{x}$	T12	ACD transformation followed by $x^{0.5}$
T6	$x^1 = x$ (linear)	T13	ACD transformation followed by x^1
T7	$x^2 = x*x$	T14	ACD transformation followed by x^2

ACD: approximate cumulative distribution function, a transformation to model a sigmoid relationship. A pure sigmoid relationship has an asymptote at both ends of the range of a continuous covariable.[11] This type of curve is common for pharmacokinetics.[196]

Supplemental Figures

Figure S1. Map of sites included in the analyses

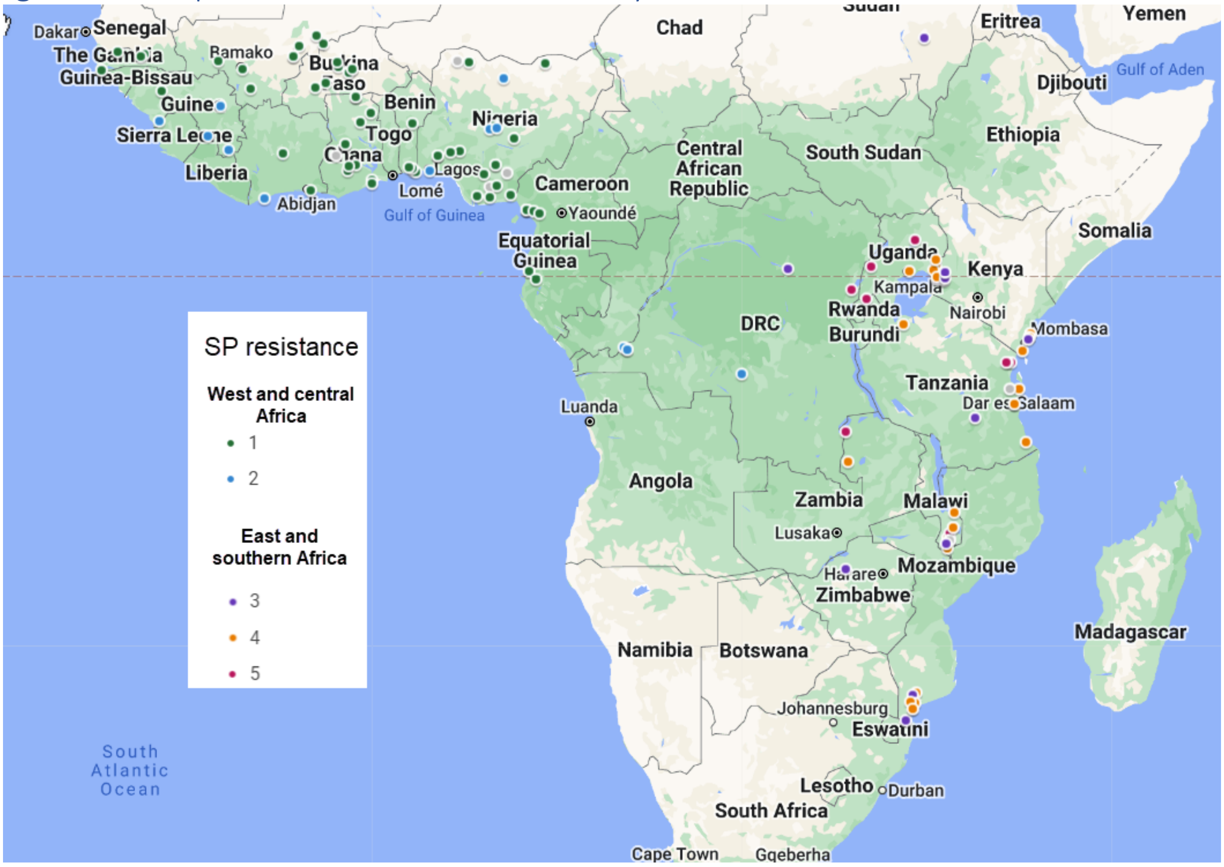

SP, sulfadoxine-pyrimethamine
SP resistance category at time of study:
-West and central Africa: 1: Very low Lys540Glu <4%; 2: Low Lys540Glu ≥ 4%
-East and southern Africa: 3: Moderate, Lys540Glu<60 & Ala581Gly<5%; 4 High Lys540Glu≥60 & Ala581Gly<5%; 5: Very high Lys540Glu≥60 & Ala581Gly≥5%.

Figure S2. Relationship between the prevalence of the *dhps* Ala437Gly and *dhps* Lys540EGlu mutation in the study locations in central and west Africa and east and southern Africa

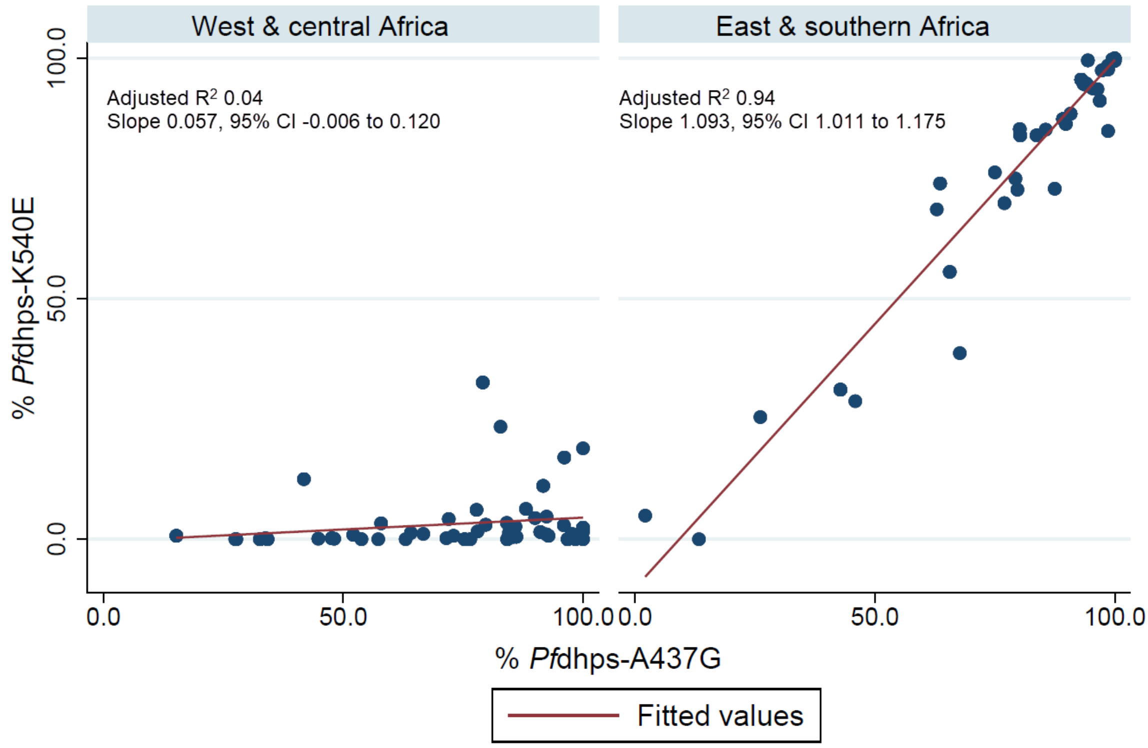

Pfdhps, *Plasmodium falciparum* dihydropteroate synthetase

Figure S3. Transformation used for analyses and transformations with the highest AICs for each outcome

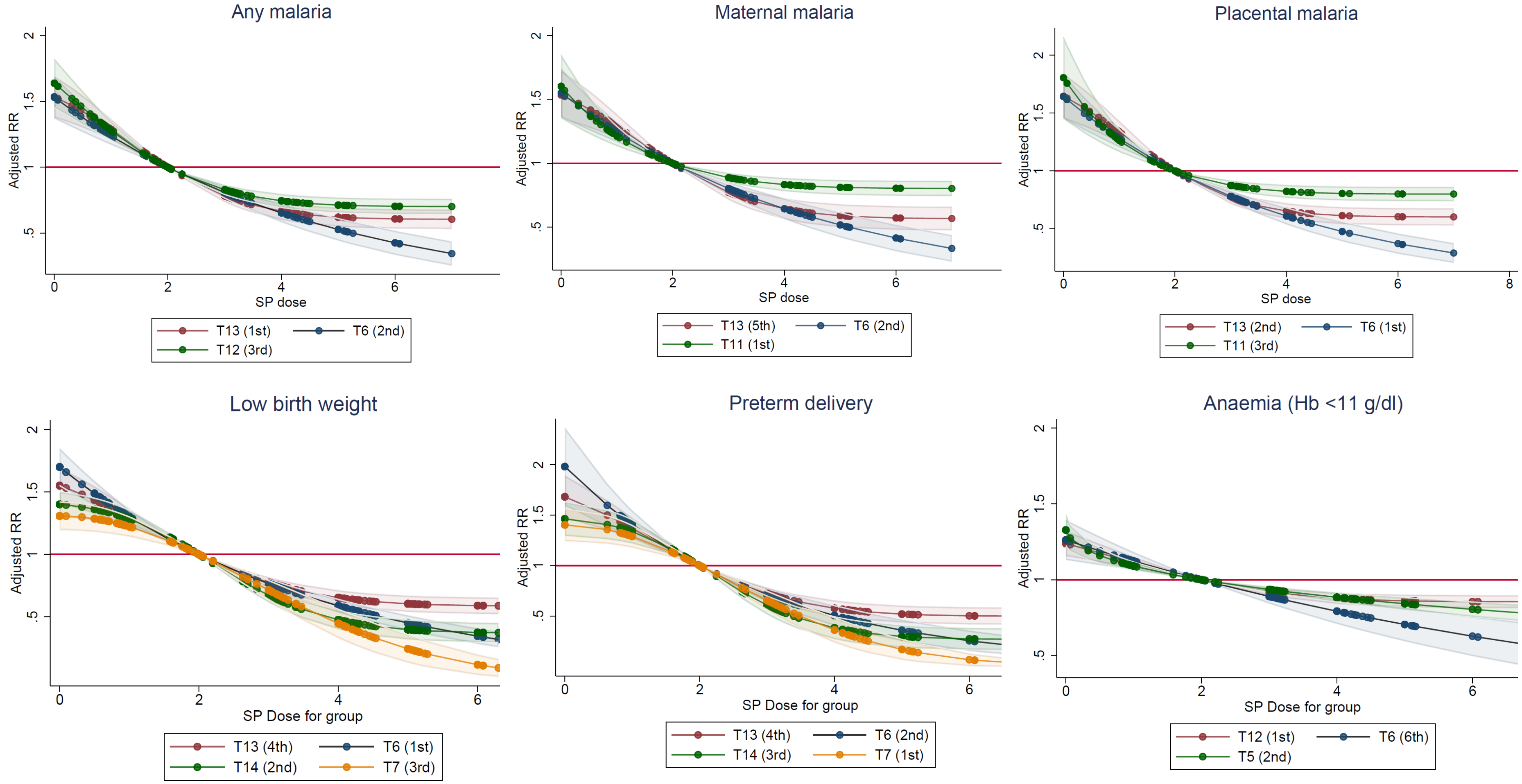

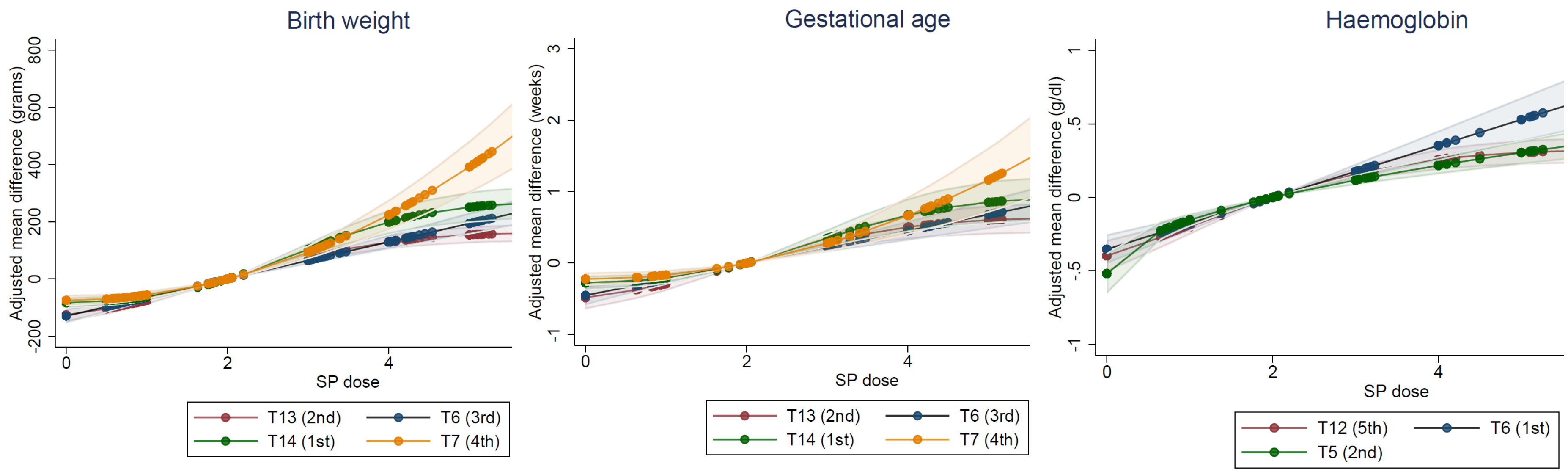

AIC, Akaika information criterion. For an explanation of the models, see table S9b. In brackets, the ranking of the model by AIC. For a malaria outcome (any malaria, maternal malaria, placental malaria) the transformation T13 was used. For the morbidity outcomes low birth weight, prematurity, birth weight and gestational age, T14 was used. For the morbidity outcomes of anaemia and haemoglobin at delivery or in the third trimester, the transformation T5 was used. For these transformations, the effect of SP resistance on the outcome by region was examined.

Supplemental References

1. van Eijk AM, Larsen DA, Kayentao K, et al. Effect of Plasmodium falciparum sulfadoxine-pyrimethamine resistance on the effectiveness of intermittent preventive therapy for malaria in pregnancy in Africa: a systematic review and meta-analysis. *Lancet Infect Dis* 2019; **19**: 546-56.
2. WWARN. Malaria in Pregnancy Library. 2024. https://mip.wwarn.org/ (accessed Jan 5, 2025).
3. Fehintola AO, Fehintola FO, Loto OM, Fasubaa OB, Bakare B, Ogundele O. Pregnancy and fetal outcome of placental malaria parasitemia in Ile-Ife, Nigeria. *Trop J Obstet Gynaecol* 2016; **33**: 310–6.
4. Nduka FO, Nwosu E, Oguariri RM. Evaluation of the effectiveness and compliance of intermittent preventive treatment (IPT) in the control of malaria in pregnant women in south eastern Nigeria. *Ann Trop Med Parasitol* 2011; **105**: 599–605.
5. van Eijk AM, Hill J, Povall S, Reynolds A, Wong H, Ter Kuile FO. The Malaria in Pregnancy Library: a bibliometric review. *Malar J* 2012; **11**: 362.
6. Flegg JA, Humphreys GS, Montanez B, et al. Spatiotemporal spread of Plasmodium falciparum mutations for resistance to sulfadoxine-pyrimethamine across Africa, 1990-2020. *PLoS Comput Biol* 2022; **18**: e1010317.
7. Foo YS, Flegg JA. A spatio-temporal model of multi-marker antimalarial resistance. *J R Soc Interface* 2024; **21**: 20230570.
8. Okell LC, Griffin JT, Roper C. Mapping sulphadoxine-pyrimethamine-resistant Plasmodium falciparum malaria in infected humans and in parasite populations in Africa. *Sci Rep* 2017; **7**: 7389.
9. Pearce RJ, Pota H, Evehe MS, et al. Multiple origins and regional dispersal of resistant dhps in African Plasmodium falciparum malaria. *PLoS Med* 2009; **6**: e1000055.
10. Walker PG, Floyd J, Ter Kuile F, Cairns M. Estimated impact on birth weight of scaling up intermittent preventive treatment of malaria in pregnancy given sulphadoxine-pyrimethamine resistance in Africa: A mathematical model. *PLoS Med* 2017; **14**: e1002243.
11. Royston P. A smooth covariate rank transformation for use in regression models with a sigmoid dose-response function. *Stata J* 2014; **14**: 329-41.
12. Curtin University, Telethon Kids Institute. The Malaria Atlas Project. 2024. http://www.map.ox.ac.uk/ (accessed (Jan 5, 2025).
13. Accrombessi M, Fievet N, Yovo E, et al. Prevalence and associated risk factors of malaria in the first trimester of pregnancy: A preconceptional cohort study in Benin. *J Infect Dis* 2018; **217**: 1309-17.
14. Aduloju OP, Ade-Ojo IP, Olaogun OD, Olofinbiyi BA, Akintayo AA. Effect of intermittent preventive treatment of malaria on the outcome of pregnancy among women attending antenatal clinic of a Nigerian Teaching Hospital. *Trop J Obstet Gynaecol* 2013; **30**: 7–15.
15. Agomo CO, Oyibo WA, Odukoya-Maije F. Parasitological assessment of two-dose and monthly intermittent preventive treatment of malaria during pregnancy with sulfadoxine-pyrimethamine (IPTp-SP) in Lagos, Nigeria. *Malar Res Treat* 2011; **Article ID 932895**: 1-6.
16. Agyeman YN, Newton SK, Annor RB, Owusu-Dabo E. The Effectiveness of the Revised Intermittent Preventive Treatment with Sulphadoxine Pyrimethamine (IPTp-SP) in the Prevention of Malaria among Pregnant Women in Northern Ghana. *J Trop Med* 2020; **2020**: 2325304.
17. Akinnawo A, Seyram K, Kaali EB, et al. Assessing the relationship between gravidity and placental malaria among pregnant women in a high transmission area in Ghana. *Malar J* 2022; **21**: 240.
18. Alli LA, Isah AY, Jamda MA, Adesokan AA. Use of intermittent preventive treatment for malaria among pregnant women in Kubwa, Abuja, Nigeria. *Int J Trop Dis Health* 2013; **3**: 339–45.
19. Anchang-Kimbi JK, Achidi EA, Nkegoum B, Sverremark-Ekstrom E, Troye-Blomberg M. Diagnostic comparison of malaria infection in peripheral blood, placental blood and placental biopsies in Cameroonian parturient women. *Malar J* 2009; **8**: 126.

20. Anchang-Kimbi JK, Kalaji LN, Mbacham HF, et al. Coverage and effectiveness of intermittent preventive treatment in pregnancy with sulfadoxine-pyrimethamine (IPTp-SP) on adverse pregnancy outcomes in the Mount Cameroon area, South West Cameroon. *Malar J* 2020; **19**: 100.
21. Anto F, Agongo IH, Asoala V, Awini E, Oduro AR. Intermittent Preventive Treatment of Malaria in Pregnancy: Assessment of the Sulfadoxine-Pyrimethamine Three-Dose Policy on Birth Outcomes in Rural Northern Ghana. *J Trop Med* 2019: 6712685.
22. Apinjoh TO, Anchang-Kimbi JK, Mugri RN, et al. Determinants of infant susceptibility to malaria during the first year of life in South Western Cameroon. *Open Forum Infect Dis* 2015; **2**: ofv012.
23. Apinjoh TO, Ntui VN, Chi HF, et al. Intermittent preventive treatment with Sulphadoxine-Pyrimethamine (IPTp-SP) is associated with protection against sub-microscopic P. falciparum infection in pregnant women during the low transmission dry season in southwestern Cameroon: A semi-longitudinal study. *PLoS One* 2022; **17**: e0275370.
24. Appiah PC, Arhin-Wiredu K, Adjei MR, et al. Stillbirths in primary level hospitals in Sunyani, Ghana: a retrospective data analysis. *Asian J Pregnancy Childbirth* 2020; **2**: 1-8.
25. Arinaitwe E, Ades V, Walakira A, et al. Intermittent preventive therapy with sulfadoxine-pyrimethamine for malaria in pregnancy: a cross-sectional study from Tororo, Uganda. *PLoS One* 2013; **8**: e73073.
26. Arnaldo P, Rovira-Vallbona E, Langa JS, et al. Uptake of intermittent preventive treatment and pregnancy outcomes: health facilities and community surveys in Chokwe district, southern Mozambique. *Malar J* 2018; **17**: 109.
27. Asamoah GD, Adoba P, Edzeamey FJ, et al. Asymptomatic malaria and its effect on parturients who received intermittent preventive treatment, a cross sectional study of the Bekwai District Ghana *GSC Biol Pharm Sci* 2018; **4**: 7-16.
28. Aziken ME, Akubuo KK, Gharoro EP. Efficacy of intermittent preventive treatment with sulfadoxine-pyrimethamine on placental parasitemia in pregnant women in midwestern Nigeria. *Int J Gynaecol Obstet* 2011; **112**: 30–3.
29. Bedia-Tanoh AV, Konaté A, Gnagne AP, et al. Effectiveness of intermittent preventive treatment with Sulfadoxine-Pyrimethamine in pregnant women in San Pedro, Côte D'Ivoire. *Pathog Glob Health* 2021: 1-6.
30. Biaou COA, Kpozehouen A, Glele-Ahanhanzo Y, Ayivi-Vinz G, Ouro-Koura AR, Azandjeme C. Sulfadoxine-pyrimethamine-based intermittent preventive treatment in pregnant women and its effect on birth weight: application of 3-dosing regimen in the urban area of South Benin in 2017. *Pan Afr Med J* 2019; **34**: 155.
31. Bouyou-Akotet MK, Nzenze-Afene S, Ngoungou EB, et al. Burden of malaria during pregnancy at the time of IPTp/SP implementation in Gabon. *Am J Trop Med Hyg* 2010; **82**: 202–9.
32. Bouyou-Akotet MK, Mawili-Mboumba DP, Kendjo E, et al. Decrease of microscopic *Plasmodium falciparum* infection prevalence during pregnancy following IPTp-SP implementation in urban cities of Gabon. *Trans R Soc Trop Med Hyg* 2016; **110**: 333–42.
33. Braun V, Rempis E, Schnack A, et al. Lack of effect of intermittent preventive treatment for malaria in pregnancy and intense drug resistance in western Uganda. *Malar J* 2015; **14**: 372.
34. Cassam Y. The effect of falciparum malaria prevalence on the effectiveness of intermittent preventive treatment with sulfadoxine-pyrimethamine during pregnancy in reducing low birth weight in southern Mozambique. Pretoria, South Africa: University of Pretoria; 2007.
35. Challis K, Osman NB, Cotiro M, Nordahl G, Dgedge M, Bergstrom S. Impact of a double dose of sulphadoxine-pyrimethamine to reduce prevalence of pregnancy malaria in southern Mozambique. *Trop Med Int Health* 2004; **9**: 1066–73.
36. Chico RM, Chaponda EB, Ariti C, Chandramohan D. Sulfadoxine-Pyrimethamine exhibits dose-response protection against adverse birth outcomes related to malaria and sexually transmitted and reproductive tract infections. *Clin Infect Dis* 2017; **64**: 1043-51.

37. Chukwuocha UM, Nwakwuo GC, Alinnor LO. Knowledge and utilization of preventive measures in the control of neonatal malaria in south-eastern Nigeria. *Tanzan J Health Res* 2016; **18**: 1–8.
38. Cohee LM, Kalilani-Phiri L, Boudova S, et al. Submicroscopic malaria infection during pregnancy and the impact of intermittent preventive treatment. *Malar J* 2014; **13**: 274.
39. Cosmic Consortium. Community-based malaria Screening and treatment for pregnant women receiving standard intermittent preventive treatment with sulfadoxine-pyrimethamine: A multicentre (The Gambia, Burkina Faso and Benin) cluster randomised controlled trial. *Clin Infect Dis* 2018.
40. Coulibaly SO, Kayentao K, Taylor S, et al. Parasite clearance following treatment with sulphadoxine-pyrimethamine for intermittent preventive treatment in Burkina-Faso and Mali: 42-day in vivo follow-up study. *Malar J* 2014; **13**: 41.
41. Desai M, Gutman J, L'lanziva A, et al. Intermittent screening and treatment or intermittent preventive treatment with dihydroartemisinin-piperaquine versus intermittent preventive treatment with sulfadoxine-pyrimethamine for the control of malaria during pregnancy in western Kenya: an open-label, three-group, randomised controlled superiority trial. *Lancet* 2015; **386**: 2507-19.
42. Desai M, Gutman J, Taylor SM, et al. Impact of sulfadoxine-pyrimethamine resistance on effectiveness of intermittent preventive therapy for malaria in pregnancy at clearing infections and preventing low birth weight. *Clin Infect Dis* 2016; **62**: 323–33.
43. Diakite OSM, Kayentao K, Traore BT, et al. Superiority of 3 doses over 2 doses of intermittent preventive treatment with sulfadoxine-pyrimethamine for the prevention of malaria during pregnancy in Mali: a randomized controlled trial. *Clin Infect Dis* 2011; **53**: 215-23.
44. Dosoo DK, Malm K, Oppong FB, et al. Effectiveness of intermittent preventive treatment in pregnancy with sulphadoxine-pyrimethamine (IPTp-SP) in Ghana. *BMJ Glob Health* 2021; **6**.
45. Douamba Z, Dao NG, Zohoncon TM, et al. Mother-to-Children Plasmodium falciparum Asymptomatic Malaria Transmission at Saint Camille Medical Centre in Ouagadougou, Burkina Faso. *Malar Res Treat* 2014; **2014**: 390513.
46. Epuitai J. Prevalence and factors associated with placental malaria parasitemia among women of Lira regional referral hospital: Makerere University, Kampala, Uganda; 2019.
47. Falade CO, Yusuf BO, Fadero FF, Mokuolu OA, Hamer DH, Salako LA. Intermittent preventive treatment with sulphadoxine-pyrimethamine is effective in preventing maternal and placental malaria in Ibadan, south-western Nigeria. *Malar J* 2007; **6**: 88.
48. Famanta A, Diakite M, Diawara SI, et al. Prevalence of maternal and placental malaria and of neonatal low birth weight in a semi-urban area of Bamako (Mali). *Sante* 2011; **21**: 3–7.
49. Fehintola AO. Prevalence and risk factors for placental parasitaemia at delivery among pregnant women in Ile Ife, Nigeria. *Int J Clin Med Cancer Res* 2015.
50. Feng G, Simpson JA, Chaluluka E, Molyneux ME, Rogerson SJ. Decreasing burden of malaria in pregnancy in Malawian women and its relationship to use of intermittent preventive therapy or bed nets. *PLoS One* 2010; **5**: e12012.
51. Filler SJ, Kazembe P, Thigpen M, et al. Randomized trial of 2-dose versus monthly sulfadoxine-pyrimethamine intermittent preventive treatment for malaria in HIV-positive and HIV-negative pregnant women in Malawi. *J Infect Dis* 2006; **194**: 286-93.
52. Gies S, Coulibaly SO, Ouattara FT, D'Alessandro U. Individual efficacy of intermittent preventive treatment with sulfadoxine-pyrimethamine in primi- and secundigravidae in rural Burkina Faso: impact on parasitaemia, anaemia and birth weight. *Trop Med Int Health* 2009; **14**: 174–82.
53. Gutman G, Mwandama D, Wiegand RE, Ali D, Mathanga DP, Skarbinski J. Effectiveness of intermittent preventive treatment with sulfadoxine-pyrimethamine in pregnancy on maternal and infant birth outcomes in Machinga District, Malawi. *J Infect Dis* 2013; **208**: 907–16.
54. Kalilani L, Taylor S, Madanitsa M, et al. Waning effectiveness of intermittent preventive treatment in pregnancy (IPTp) with sulphadoxine-pyrimethamine (SP) in the presence of high SP Resistance in Malawi. *Am J Trop Med Hyg* 2011; **85**: 354-5 (abstr).

55. Gutman J, Mwandama D, Banda J, et al. Intermittent preventive treatment continues to provide benefit to Malawian pregnant women. *Am J Trop Med Hyg* 2016; **95**: 270.
56. Harrington WE, Mutabingwa TK, Kabyemela E, Fried M, Duffy PE. Intermittent treatment to prevent pregnancy malaria does not confer benefit in an area of widespread drug resistance. *Clin Infect Dis* 2011; **53**: 224–30.
57. Hommerich L, von Oertzen C, Bedu-Addo G, et al. Decline of placental malaria in southern Ghana after the implementation of intermittent preventive treatment in pregnancy. *Malar J* 2007; **6**: 144.
58. Igboeli NU, Ukwe CV, Aguwa CN. Effect of antimalarial prophylaxis with sulphadoxine-pyrimethamine on pregnancy outcomes in Nsukka, Nigeria. *MalariaWorld J* 2017; **8**: 3.
59. Igboeli U, Adibe MO, Ukwe CV, Aguwa CN. Comparison of the effectiveness of two-dose versus three-dose sulphadoxine-pyrimethamine in preventing adverse pregnancy outcomes in Nigeria. *J Vector Borne Dis* 2018; **55**: 197-202.
60. Inyang-Etoh EC, Agan TU, Etuk SJ, Inyang-Etoh PC. The role of prophylactic antimalarial in the reduction of placental parasitemia among pregnant women in Calabar, Nigeria. *Niger Med J* 2011; **52**: 235–8.
61. Isah DA, Isah AY, Thairu Y, Agida ET. Effectiveness of 3 doses of intermittent preventive therapy with sulphadoxine-pyrimethamine in pregnancy. *Ann Med Health Sci Res* 2017; **7**: 52-7.
62. Kajubi R, Ochieng T, Kakuru A, et al. Monthly sulfadoxine-pyrimethamine versus dihydroartemisinin-piperaquine for intermittent preventive treatment of malaria in pregnancy: a double-blind, randomised, controlled, superiority trial. *Lancet* 2019; **393**: 1428-39.
63. Kalayjian BC, Malhotra I, Mungai P, Holding P, King CL. Marked decline in malaria prevalence among pregnant women and their offspring from 1996 to 2010 on the south Kenyan Coast. *Am J Trop Med Hyg* 2013; **89**: 1129-34.
64. Kalilani L, Mofolo I, Chaponda M, Rogerson SJ, Meshnick SR. The effect of timing and frequency of Plasmodium falciparum infection during pregnancy on the risk of low birth weight and maternal anemia. *Trans R Soc Trop Med Hyg* 2010; **104**: 416-22.
65. Kamau A, Musau M, Mwakio S, et al. The impact of intermittent presumptive treatment for malaria in pregnancy on hospital birth outcomes on the Kenyan coast. *Clin Infect Dis* 2022; **76**: e875-83.
66. Kayentao K. Burden of malaria in pregnancy in Mali and impact of dosing frequency and antimalarial drug resistance on the effectiveness of intermittent preventive therapy in pregnancy in Africa. Liverpool, UK: Liverpool School of Tropical Medicine; 2014.
67. Kayiba NK, Yobi DM, Tchakounang VRK, et al. Evaluation of the usefulness of intermittent preventive treatment of malaria in pregnancy with sulfadoxine-pyrimethamine in a context with increased resistance of Plasmodium falciparum in Kingasani Hospital, Kinshasa in the Democratic Republic of Congo. *Infect Genet Evol* 2021; **94**: 105009.
68. Kilauzi AL, Mulumba JGT, Matindii BA, Tamfum JJM, Ngongo LO, Mengema B. Field utilization patterns of insecticide-treated net and intermittent preventive treatment with sulphadoxine-pyrimethamine in a resource poor endemic area: Patterns' associations with adverse mother or birth outcomes. *Ann Trop Med Public Health* 2013; **6**: 603–7.
69. Lash RR, Lemwayi R, Assenga M, et al. Factors associated with achieving antenatal care (ANC) attendance and intermittent preventive treatment in pregnancy (IPTp) recommendations in Geita Region, Tanzania, 2019. *Am J Trop Med Hyg* 2020; **103**: 74.
70. Likwela JL, D'Alessandro U, Lokwa BL, Meuris S, Dramaix MW. Sulfadoxine-pyrimethamine resistance and intermittent preventive treatment during pregnancy: a retrospective analysis of birth weight data in the Democratic Republic of Congo (DRC). *Trop Med Int Health* 2012; **17**: 322–9.
71. Lingani M, Zango SH, Valea I, et al. Low birth weight and its associated risk factors in a rural health district of Burkina Faso: a cross sectional study. *BMC Pregnancy Childbirth* 2022; **22**: 228.

72. Luntamo M, Rantala AM, Meshnick SR, et al. The effect of monthly sulfadoxine-pyrimethamine, alone or with azithromycin, on PCR-diagnosed malaria at delivery: a randomized controlled trial. *PLoS One* 2012; **7**: e41123.
73. MacArthur JR, Kabanywanyi AM, Baja A, et al. Efficacy of intermittent treatment with sulfadoxine-pyrimethamine alone or sulfadoxine-pyrimethamine plus artesunate for prevention of placental malaria in Tanzania. *Am J Trop Med Hyg* 2007; **77**: 238 (abstr).
74. Mace KE, Chalwe V, Katalenich BL, et al. Evaluation of sulphadoxine-pyrimethamine for intermittent preventive treatment of malaria in pregnancy: a retrospective birth outcomes study in Mansa, Zambia. *Malar J* 2015; **14**: 69.
75. Madanitsa M, Kalilani L, Mwapasa V, et al. Scheduled Intermittent Screening with Rapid Diagnostic Tests and Treatment with Dihydroartemisinin-Piperaquine versus Intermittent Preventive Therapy with Sulfadoxine-Pyrimethamine for Malaria in Pregnancy in Malawi: An Open-Label Randomized Controlled Trial. *PLoS Med* 2016; **13**: e1002124.
76. Madanitsa M, Barsosio HC, Minja DTR, et al. Effect of monthly intermittent preventive treatment with dihydroartemisinin-piperaquine with and without azithromycin versus monthly sulfadoxine-pyrimethamine on adverse pregnancy outcomes in Africa: a double-blind randomised, partly placebo-controlled trial. *Lancet* 2023; **401**: 1020-36.
77. Mahamar A, Andemel N, Swihart B, et al. Malaria Infection Is Common and Associated With Perinatal Mortality and Preterm Delivery Despite Widespread Use of Chemoprevention in Mali: An Observational Study 2010 to 2014. *Clin Infect Dis* 2021; **73**: 1355-61.
78. Malpass A, Chinkhumba J, Davlantes E, et al. Malaria knowledge and experiences with community health workers among recently pregnant women in Malawi. *Malar J* 2020; **19**: 154.
79. Massamba JE, Djontu JC, Vouvoungui CJ, Kobawila C, Ntoumi F. Plasmodium falciparum multiplicity of infection and pregnancy outcomes in Congolese women from southern Brazzaville, Republic of Congo. *Malar J* 2022; **21**: 114.
80. Matambisso G, Brokhattingen N, Maculuve S, et al. Sustained clinical benefit of malaria chemoprevention with sulfadoxine-pyrimethamine (SP) in pregnant women in a region with high SP resistance markers. *J Infect* 2024; **88**: 106144.
81. Mbaye A, Richardson K, Balajo B, et al. A randomized, placebo-controlled trial of intermittent preventive treatment with sulphadoxine-pyrimethamine in Gambian multigravidae. *Trop Med Int Health* 2006; **11**: 992–1002.
82. Menendez C, Bardaji A, Sigauque B, et al. A randomized placebo-controlled trial of intermittent preventive treatment in pregnant women in the context of insecticide treated nets delivered through the antenatal clinic. *PLoS One* 2008; **3**: e1934.
83. Mikomangwa WP, Minzi O, Mutagonda R, et al. Effect of sulfadoxine-pyrimethamine doses for prevention of malaria during pregnancy in hypoendemic area in Tanzania. *Malar J* 2020; **19**: 160.
84. Minja DT, Schmiegelow C, Mmbando B, et al. *Plasmodium falciparum* mutant haplotype infection during pregnancy associated with reduced birthweight, Tanzania. *Emerg Infect Dis* 2013; **19**: 1446–54.
85. Mlugu EM, Minzi O, Asghar M, Farnert A, Kamuhabwa AAR, Aklillu E. Effectiveness of sulfadoxine-pyrimethamine for intermittent preventive treatment of malaria and adverse birth outcomes in pregnant women. *Pathogens* 2020; **9**.
86. Mlugu EM, Minzi O, Kamuhabwa AAR, Aklillu E. Effectiveness of Intermittent Preventive Treatment With Dihydroartemisinin-Piperaqunine Against Malaria in Pregnancy in Tanzania: A Randomized Controlled Trial. *Clin Pharmacol Ther* 2021; **110**: 1478-89.
87. Moleins I, Agnamey P. Malaria and pregnancy: impact of ontermittent preventive treatment with sulfadoxine-pyrimethamine on weight at birth at the Oussouye maternity (Casamance, Senegal). *Revue Sage-Femme* 2010; **9**: 123–7.
88. Mosha D, Chilongola J, Ndeserua R, Mwingira F, Genton B. Effectiveness of intermittent preventive treatment with sulfadoxine-pyrimethamine during pregnancy on placental malaria,

maternal anaemia and birthweight in areas with high and low malaria transmission intensity in Tanzania. *Trop Med Int Health* 2014; **19**: 1048–56.
89. Moukoko CEE, Foko LPK, Ayina A, et al. Effectiveness of Intermittent Preventive Treatment with Sulfadoxine- Pyrimethamine in Pregnancy: Low Coverage and High Prevalence of Plasmodium falciparum dhfr-dhps Quintuple Mutants as Major Challenges in Douala, an Urban Setting in Cameroon. *Pathogens* 2023; **12**: 844.
90. Msyamboza KP, Savage EJ, Kazembe PN, et al. Community-based distribution of sulfadoxine-pyrimethamine for intermittent preventive treatment of malaria during pregnancy improved coverage but reduced antenatal attendance in southern Malawi. *Trop Med Int Health* 2009; **14**: 183–9.
91. Muchekeza M, Chadambuka A, Tishmanga M, et al. Pregnancy outcomes for women receiving and not receiving intermittent preventive treatment for malaria in pregnancy using sulphadoxine-pyrimethamine in Gokwe North, Midlands Province, 2011. *Nursing Prim Care* 2018; **2**: 1-6.
92. Muhammad HU, Giwa FJ, Olayinka AT, et al. Malaria prevention practices and delivery outcome: a cross sectional study of pregnant women attending a tertiary hospital in northeastern Nigeria. *Malar J* 2016; **15**: 326.
93. Mwangi MN, Roth JM, Smit MR, et al. Effect of daily antenatal iron supplementation on Plasmodium infection in Kenyan women: A randomized clinical trial. *JAMA* 2015; **314**: 1009–20.
94. Mwapasa V. The interactions between *Plasmodium falciparum* malaria and HIV-1 in pregnant Malawian women. Chapel Hill, Michigan: University of Michigan; 2004.
95. Namusoke F, Rasti N, Kironde F, Wahlgren M, Mirembe F. Malaria burden in pregnancy at Mulago National Referral Hospital in Kampala, Uganda. *Malar Res Treat* 2010: Article ID 913857.
96. Ndeserua R, Juma A, Mosha D, Chilongola J. Risk factors for placental malaria and associated adverse pregnancy outcomes in Rufiji, Tanzania: a hospital based cross sectional study. *Afr Health Sci* 2015; **15**: 810–8.
97. Ndyomugyenyi R, Clarke SE, Hutchison CL, Hansen KS, Magnussen P. Efficacy of malaria prevention during pregnancy in an area of low and unstable transmission: an individually-randomised placebo-controlled trial using intermittent preventive treatment and insecticide-treated nets in the Kabale Highlands, southwestern Uganda. *Trans R Soc Trop Med Hyg* 2011; **105**: 607–16.
98. Nganda RY, Drakeley C, Reyburn H, Marchant T. Knowledge of malaria influences the use of insecticide treated nets but not intermittent presumptive treatment by pregnant women in Tanzania. *Malar J* 2004; **3**: 42.
99. Njagi JK. The effects of sulfadoxine-pyrimethamine intermittent treatment and pyrethroid impregnated bed nets on malaria morbidity in pregnancy and birth weight in Bondo district, Kenya. Nairobi, Kenya: University of Nairobi; 2002.
100. Obi SS, Sumner WD, Garrett-Cherry T, et al. Asymptomatic malaria infection impact on maternal anemia in Delta State, Nigeria. *Int J Health Med Nursing Practice* 2022; **4**: 48-76.
101. Oduro AR, Fryauff DJ, Koram KA, et al. Sulfadoxine-pyrimethamine-based intermittent preventive treatment, bed net use, and antenatal care during pregnancy: demographic trends and impact on the health of newborns in the Kassena Nankana District, northeastern Ghana. *Am J Trop Med Hyg* 2010; **83**: 79–89.
102. Olliaro PL, Delenne H, Cisse M, et al. Implementation of intermittent preventive treatment in pregnancy with sulphadoxine/pyrimethamine (IPTp-SP) at a district health centre in rural Senegal. *Malar J* 2008; **7**: 234.
103. Olorunda DC, Ajayi IO, Falade CO. Do frequent antenatal care visits ensure access and adherence to intermittent preventive treatment of malaria in pregnancy in an urban hospital in South West Nigeria? *Afr J Biomed Res* 2013; **16**: 153–61.
104. Onoja H, Nduka F, Abah AE. Effects of intervention schemes on sulphadoxine-pyrimethamine compliance among pregnant women in a health facility in Port Harcourt, Rivers State, Nigeria. *Int J Infect* 2021; **8**: e107940.

105. Onyebuchi AK, Lawani LO, Iyoke CA, Onoh CR, Okeke NE. Adherence to intermittent preventive treatment for malaria with sulphadoxine-pyrimethamine and outcome of pregnancy among parturients in South East Nigeria. *Patient Prefer Adherence* 2014; **8**: 447–52.
106. Orobaton N, Austin AM, Abegunde D, et al. Scaling-up the use of sulfadoxine-pyrimethamine for the preventive treatment of malaria in pregnancy: results and lessons on scalability, costs and programme impact from three local government areas in Sokoto State, Nigeria. *Malar J* 2016; **15**: 533.
107. Ouma P, Were F, Were V, et al. Effectiveness of intermittent preventive treatment with sulfadoxine-pyrimethamine in pregnant women in Western Kenya: results of an observational study. *Am J Trop Med Hyg* 2012; **87**: 435 (abstr).
108. Oweisi PW, Omietimi JE, John CT, Aigere EOS, Allagoa DO, Kotingo EL. Correlation between placental malaria parasitaemia at delivery and infant birth weight in a Nigerian tertiary health centre. *Niger J Med* 2018; **27**.
109. Parise ME, Ayisi JG, Nahlen BL, et al. Efficacy of sulfadoxine-pyrimethamine for prevention of placental malaria in an area of Kenya with a high prevalence of malaria and human immunodeficiency virus infection. *Am J Trop Med Hyg* 1998; **59**: 813–22.
110. Ramharter M, Schuster K, Bouyou-Akotet MK, et al. Malaria in pregnancy before and after the implementation of a national IPTp program in Gabon. *Am J Trop Med Hyg* 2007; **77**: 418–22.
111. Rogawski ET, Chaluluka E, Molyneux ME, Feng G, Rogerson SJ, Meshnick SR. The effects of malaria and intermittent preventive treatment during pregnancy on fetal anemia in Malawi. *Clin Infect Dis* 2012; **55**: 1096–102.
112. Rogerson SJ, Chaluluka E, Kanjala M, Mkundika P, Mhango C, Molyneux ME. Intermittent sulfadoxine-pyrimethamine in pregnancy: effectiveness against malaria morbidity in Blantyre, Malawi, in 1997-99. *Trans R Soc Trop Med Hyg* 2000; **94**: 549–53.
113. Shulman CE, Dorman EK, Cutts F, et al. Intermittent sulphadoxine-pyrimethamine to prevent severe anaemia secondary to malaria in pregnancy: a randomised placebo-controlled trial. *Lancet* 1999; **353**: 632-6.
114. Sirima SB, Cotte AH, Konate A, et al. Malaria prevention during pregnancy: assessing the disease burden one year after implementing a program of intermittent preventive treatment in Koupela District, Burkina Faso. *Am J Trop Med Hyg* 2006; **75**: 205–11.
115. Stephens JK, Kyei-Baafour E, Dickson EK, et al. Effect of IPTp on *Plasmodium falciparum* antibody levels among pregnant women and their babies in a sub-urban coastal area in Ghana. *Malar J* 2017; **16**: 224.
116. Suleiman IEDE, Mohamadani AAA, Mirgani OA. Malaria propylaxis during pregnancy in primigravidae using sulfadoxine/pyimethamine in Wad Medani - Sudan. *Gezira J Health Sci* 2003; **1**: 1–9.
117. Tagbor H, Cairns M, Bojang K, et al. A non-inferiority, individually randomized trial of intermittent screening and treatment versus intermittent preventive treatment in the control of malaria in pregnancy. *PLoS One* 2015; **10**: e0132247.
118. Tetteh-Ashong E. Evaluation of a screening method to assess the efficacy of intermittent preventive treatment with SP in pregnant women in Malawi. Liverpool, UK: Liverpool School of Tropical Medicine; 2005.
119. Tonga C, Kimbi HK, Anchang-Kimbi JK, Nyabeyeu HN, Bissemou ZB, Lehman LG. Malaria risk factors in women on intermittent preventive treatment at delivery and their effects on pregnancy outcome in Sanaga-Maritime, Cameroon. *PLoS One* 2013; **8**: e65876.
120. Tongo OO, Orimadegun AE, Akinyinka OO. Utilisation of malaria preventive measures during pregnancy and birth outcomes in Ibadan, Nigeria. *BMC Pregnancy Childbirth* 2011; **11**: 60.
121. Toure OA, Kone PL, Coulibaly ML, et al. Coverage and efficacy of intermittent preventive treatment with sulphadoxine pyrimethamine against malaria in pregnancy in Cote d'Ivoire five years after its implementation. *Parasit Vectors* 2014; **7**: 495.

122. Toure AA, Doumbouya A, Diallo A, et al. Malaria-associated factors among pregnant women in Guinea. *J Trop Med* 2019.
123. Kabalu Tshiongo J, Zola Matuvanga T, Mitashi P, et al. Prevention of Malaria in Pregnant Women and Its Effects on Maternal and Child Health, the Case of Centre Hospitalier de Kingasani II in the Democratic Republic of the Congo. *Trop Med Infect Dis* 2024; **9**.
124. Tutu EO, Browne E, Lawson B. Effect of sulphadoxine-pyrimethamine on neonatal birth weight and perceptions on its impact on malaria in pregnancy in an intermittent preventive treatment programme setting in Offinso District, Ghana. *Int Health* 2011; **3**: 206–12.
125. Umemmuo MU, Agboghoroma CO, Iregbu KC. The efficacy of intermittent preventive therapy in the eradication of peripheral and placental parasitemia in a malaria-endemic environment, as seen in a tertiary hospital in Abuja, Nigeria. *Int J Gynaecol Obstet* 2020.
126. Valea I, Tinto H, Drabo MK, et al. Intermittent preventive treatment of malaria with sulphadoxine-pyrimethamine during pregnancy in Burkina Faso: effect of adding a third dose to the standard two-dose regimen on low birth weight, anaemia and pregnancy outcomes. *Malar J* 2010; **9**: 324.
127. van Eijk AM, Ayisi JG, ter Kuile FO, et al. Effectiveness of intermittent preventive treatment with sulphadoxine-pyrimethamine for control of malaria in pregnancy in western Kenya: a hospital-based study. *Trop Med Int Health* 2004; **9**: 351–60.
128. van Spronsen JH, Schneider TA, Atasige S. Placental malaria and the relationship to pregnancy outcome at Gushegu District Hospital, Northern Ghana. *Trop Doct* 2012; **42**: 80–4.
129. Vanga-Bosson HA, Coffie PA, Kanhon S, et al. Coverage of intermittent prevention treatment with sulphadoxine-pyrimethamine among pregnant women and congenital malaria in Cote d'Ivoire. *Malar J* 2011; **10**: 105.
130. Verhoeff FH, Brabin BJ, Chimsuku L, Kazembe P, Russell WB, Broadhead RL. An evaluation of the effects of intermittent sulfadoxine-pyrimethamine treatment in pregnancy on parasite clearance and risk of low birthweight in rural Malawi. *Ann Trop Med Parasitol* 1998; **92**: 141–50.
131. Vincenz C, Dolo Z, Saye S, Lovett JL, Strassmann BI. Risk factors for placental malaria, sulfadoxine-pyrimethamine doses, and birth outcomes in a rural to urban prospective cohort study on the Bandiagara Escarpment and Bamako, Mali. *Malar J* 2022; **21**: 110.
132. Waltmann A, McQuade ETR, Chinkhumba J, et al. The positive effect of malaria IPTp-SP on birthweight is mediated by gestational weight gain but modifiable by maternal carriage of enteric pathogens. *EBioMedicine* 2022; **77**: 103871.
133. Wolf K, Alao M, Binazon A, et al. Malaria in pregnancy and antenatal care knowledge, attitudes and intervention coverage in Atlantique Department, Benin. *Am J Trop Med Hyg* 2021; **105**: 88.
134. Yussuf SM. Effect of intermitent preventive treatment (IPTp) using sulphadoxine pyrimethamine (SP) on birth weight, Lindi region, 2009. Dar es Salaam, Tanzania: Muhimbili University of Health and Allied Sciences; 2010.
135. UNAIDS. AIDSinfo. 2020. http://aidsinfo.unaids.org (accessed Jan 5, 2025).
136. Huijben S, Macete E, Mombo-Ngoma G, et al. Counter-Selection of Antimalarial Resistance Polymorphisms by Intermittent Preventive Treatment in Pregnancy. *J Infect Dis* 2020; **221**: 293-303.
137. Iwalokun BA, Iwalokun SO, Adebodun V, Balogun M. Carriage of Mutant Dihydrofolate Reductase and Dihydropteroate Synthase Genes among Plasmodium falciparum Isolates Recovered from Pregnant Women with Asymptomatic Infection in Lagos, Nigeria. *Med Princ Pract* 2015; **24**: 436-43.
138. Oguike MC, Falade CO, Shu E, et al. Molecular determinants of sulfadoxine-pyrimethamine resistance in *Plasmodium falciparum* in Nigeria and the regional emergence of dhps 431V. *Int J Parasitol Drugs Drug Resist* 2016; **6**: 220–9.
139. Dieng CC, Gonzalez L, Pestana K, et al. Contrasting Asymptomatic and Drug Resistance Gene Prevalence of Plasmodium falciparum in Ghana: Implications on Seasonal Malaria Chemoprevention. *Genes (Basel)* 2019; **10**.

140. Mensah BA, Aydemir O, Myers-Hansen JL, et al. Antimalarial Drug Resistance Profiling of Plasmodium falciparum Infections in Ghana Using Molecular Inversion Probes and Next-Generation Sequencing. *Antimicrob Agents Chemother* 2020; **64**.
141. Mbacham WF, Evehe MS, Netongo PM, et al. Efficacy of amodiaquine, sulphadoxine-pyrimethamine and their combination for the treatment of uncomplicated Plasmodium falciparum malaria in children in Cameroon at the time of policy change to artemisinin-based combination therapy. *Malar J* 2010; **9**: 34.
142. L'Episcopia M, Kelley J, Djeunang Dongho BG, et al. Targeted deep amplicon sequencing of antimalarial resistance markers in Plasmodium falciparum isolates from Cameroon. *Int J Infect Dis* 2021; **107**: 234-41.
143. Zhao L, Pi L, Qin Y, et al. Widespread resistance mutations to sulfadoxine-pyrimethamine in malaria parasites imported to China from Central and Western Africa. *Int J Parasitol Drugs Drug Resist* 2020; **12**: 1-6.
144. Gupta H, Macete E, Bulo H, et al. Drug-Resistant Polymorphisms and Copy Numbers in Plasmodium falciparum, Mozambique, 2015. *Emerg Infect Dis* 2018; **24**: 40-8.
145. Smith-Aguasca R, Gupta H, Uberegui E, et al. Mosquitoes as a feasible sentinel group for anti-malarial resistance surveillance by Next Generation Sequencing of Plasmodium falciparum. *Malar J* 2019; **18**: 351.
146. Baraka V, Delgado-Ratto C, Nag S, et al. Different origin and dispersal of sulfadoxine-resistant Plasmodium falciparum haplotypes between Eastern Africa and Democratic Republic of Congo. *Int J Antimicrob Agents* 2017; **49**: 456-64.
147. Flegg JA, Patil AP, Venkatesan M, et al. Spatiotemporal mathematical modelling of mutations of the dhps gene in African *Plasmodium falciparum*. *Malar J* 2013; **12**: 249.
148. Raman J, Little F, Roper C, et al. Five years of large-scale dhfr and dhps mutation surveillance following the phased implementation of artesunate plus sulfadoxine-pyrimethamine in Maputo Province, Southern Mozambique. *Am J Trop Med Hyg* 2010; **82**: 788-94.
149. Raman J, Mauff K, Muianga P, Mussa A, Maharaj R, Barnes KI. Five years of antimalarial resistance marker surveillance in Gaza Province, Mozambique, following artemisinin-based combination therapy roll out. *PLoS One* 2011; **6**: e25992.
150. Raman J, Sharp B, Kleinschmidt I, et al. Differential effect of regional drug pressure on dihydrofolate reductase and dihydropteroate synthetase mutations in southern Mozambique. *Am J Trop Med Hyg* 2008; **78**: 256-61.
151. Siame MN, Mharakurwa S, Chipeta J, Thuma P, Michelo C. High prevalence of dhfr and dhps molecular markers in Plasmodium falciparum in pregnant women of Nchelenge district, Northern Zambia. *Malar J* 2015; **14**: 190.
152. Artimovich E, Schneider K, Taylor TE, et al. Persistence of Sulfadoxine-Pyrimethamine Resistance Despite Reduction of Drug Pressure in Malawi. *J Infect Dis* 2015; **212**: 694-701.
153. Tahita MC, Tinto H, Erhart A, et al. Prevalence of the dhfr and dhps mutations among pregnant women in rural Burkina Faso five years after the introduction of intermittent preventive treatment with sulfadoxine-pyrimethamine. *PLoS One* 2015; **10**: e0137440.
154. Access-SMC Partnership. Effectiveness of seasonal malaria chemoprevention at scale in west and central Africa: an observational study. *Lancet* 2020; **396**: 1829-40.
155. Nag S, Dalgaard MD, Kofoed PE, et al. High throughput resistance profiling of Plasmodium falciparum infections based on custom dual indexing and Illumina next generation sequencing-technology. *Sci Rep* 2017; **7**: 2398.
156. Svigel SS, Adeothy A, Kpemasse A, et al. Low prevalence of highly sulfadoxine-resistant dihydropteroate synthase alleles in Plasmodium falciparum isolates in Benin. *Malar J* 2021; **20**: 72.
157. Desai M, Gutman J, Taylor SM, et al. Impact of sulfadoxine-pyrimethamine resistance on effectiveness of intermittent preventive therapy for malaria in pregnancy at clearing infections and preventing low birth weight. *Clin Infect Dis* 2015; **62**: 323-33.

158. Kublin JG, Dzinjalamala FK, Kamwendo DD, et al. Molecular markers for failure of sulfadoxine-pyrimethamine and chlorproguanil-dapsone treatment of Plasmodium falciparum malaria. *J Infect Dis* 2002; **185**: 380-8.
159. Lin JT, Mbewe B, Taylor SM, Luntamo M, Meshnick SR, Ashorn P. Increased prevalence of dhfr and dhps mutants at delivery in Malawian pregnant women receiving intermittent preventive treatment for malaria. *Trop Med Int Health* 2013; **18**: 175-8.
160. Alam MT, de Souza DK, Vinayak S, et al. Selective sweeps and genetic lineages of Plasmodium falciparum drug -resistant alleles in Ghana. *J Infect Dis* 2011; **203**: 220-7.
161. Kayode AT, Ajogbasile FV, Akano K, et al. Polymorphisms in Plasmodium falciparum dihydropteroate synthetase and dihydrofolate reductase genes in Nigerian children with uncomplicated malaria using high-resolution melting technique. *Sci Rep* 2021; **11**: 471.
162. Xu C, Sun H, Wei Q, et al. Mutation Profile of pfdhfr and pfdhps in Plasmodium falciparum among Returned Chinese Migrant Workers from Africa. *Antimicrob Agents Chemother* 2019; **63**.
163. Conrad MD, Mota D, Musiime A, et al. Comparative Prevalence of Plasmodium falciparum Resistance-Associated Genetic Polymorphisms in Parasites Infecting Humans and Mosquitoes in Uganda. *Am J Trop Med Hyg* 2017; **97**: 1576-80.
164. Juma DW, Omondi AA, Ingasia L, et al. Trends in drug resistance codons in Plasmodium falciparum dihydrofolate reductase and dihydropteroate synthase genes in Kenyan parasites from 2008 to 2012. *Malar J* 2014; **13**: 250.
165. Kalilani L, Chaluluka E, Kalanda G, et al. Intermittent preventive therapy in pregnancy with sulphadoxine-pyrimethamine (SP); 42 day in-vivo follow up study among asymptomatic parasitemic pregnant women in an area with high SP resistance in Southern Malawi. *Am J Trop Med Hyg* 2010; **83**: 393 (abstr).
166. Omedo I, Bartilol B, Kimani D, et al. Spatio-temporal distribution of antimalarial drug resistant gene mutations in a *Plasmodium falciparum* parasite population from Kilifi, Kenya: A 25-year retrospective study. *Wellcome Open Res* 2022; **7**: 45.
167. Mandoko PN, Rouvier F, Kakina LM, et al. Prevalence of Plasmodium falciparum parasites resistant to sulfadoxine/pyrimethamine in the Democratic Republic of the Congo: emergence of highly resistant pfdhfr/pfdhps alleles. *J Antimicrob Chemother* 2018; **73**: 2704-15.
168. Karema C, Imwong M, Fanello CI, et al. Molecular correlates of high-level antifolate resistance in Rwandan children with Plasmodium falciparum malaria. *Antimicrob Agents Chemother* 2010; **54**: 477-83.
169. Cairns ME, Sagara I, Zongo I, et al. Evaluation of seasonal malaria chemoprevention in two areas of intense seasonal malaria transmission: Secondary analysis of a household-randomised, placebo-controlled trial in Hounde District, Burkina Faso and Bougouni District, Mali. *PLoS Med* 2020; **17**: e1003214.
170. Mita T, Venkatesan M, Ohashi J, et al. Limited geographical origin and global spread of sulfadoxine-resistant dhps alleles in Plasmodium falciparum populations. *J Infect Dis* 2011; **204**: 1980-8.
171. Diawara F, Steinhardt LC, Mahamar A, et al. Measuring the impact of seasonal malaria chemoprevention as part of routine malaria control in Kita, Mali. *Malar J* 2017; **16**: 325.
172. Mayor A, Serra-Casas E, Sanz S, et al. Molecular markers of resistance to sulfadoxine-pyrimethamine during intermittent preventive treatment for malaria in Mozambican infants. *J Infect Dis* 2008; **197**: 1737-42.
173. Bwire GM, Mikomangwa WP, Kilonzi M. Occurrence of septuple and elevated Pfdhfr-Pfdhps quintuple mutations in a general population threatens the use of sulfadoxine-pyrimethamine for malaria prevention during pregnancy in eastern-coast of Tanzania. *BMC Infect Dis* 2020; **20**: 530.
174. Ndiaye D, Dieye B, Ndiaye YD, et al. Polymorphism in dhfr/dhps genes, parasite density and ex vivo response to pyrimethamine in Plasmodium falciparum malaria parasites in Thies, Senegal. *Int J Parasitol Drugs Drug Resist* 2013; **3**: 135-42.

175. Bell DJ, Nyirongo SK, Mukaka M, et al. Sulfadoxine-pyrimethamine-based combinations for malaria: a randomised blinded trial to compare efficacy, safety and selection of resistance in Malawi. *PLoS One* 2008; **3**: e1578.
176. Ogouyemi-Hounto A, Ndam NT, Fadegnon G, et al. Low prevalence of the molecular markers of Plasmodium falciparum resistance to chloroquine and sulphadoxine/pyrimethamine in asymptomatic children in Northern Benin. *Malar J* 2013; **12**: 413.
177. Malamba S, Sandison T, Lule J, et al. Plasmodium falciparum dihydrofolate reductase and dihyropteroate synthase mutations and the use of trimethoprim-sulfamethoxazole prophylaxis among persons infected with human immunodeficiency virus. *Am J Trop Med Hyg* 2010; **82**: 766-71.
178. Matondo SI, Temba GS, Kavishe AA, et al. High levels of sulphadoxine-pyrimethamine resistance Pfdhfr-Pfdhps quintuple mutations: a cross sectional survey of six regions in Tanzania. *Malar J* 2014; **13**: 152.
179. Kavishe RA, Kaaya RD, Nag S, et al. Molecular monitoring of Plasmodium falciparum super-resistance to sulfadoxine-pyrimethamine in Tanzania. *Malar J* 2016; **15**: 335.
180. Lynch C, Pearce R, Pota H, et al. Emergence of a dhfr mutation conferring high-level drug resistance in Plasmodium falciparum populations from southwest Uganda. *J Infect Dis* 2008; **197**: 1598-604.
181. Kidima W, Nkwengulila G, Premji Z, Malisa A, Mshinda H. Dhfr and dhps mutations in Plasmodium falciparum isolates in Mlandizi, Kibaha, Tanzania: association with clinical outcome. *Tanzan Health Res Bull* 2006; **8**: 50-5.
182. Iriemenam NC, Shah M, Gatei W, et al. Temporal trends of sulphadoxine-pyrimethamine (SP) drug-resistance molecular markers in Plasmodium falciparum parasites from pregnant women in western Kenya. *Malar J* 2012; **11**: 134.
183. Ndiaye D, Daily JP, Sarr O, et al. Mutations in Plasmodium falciparum dihydrofolate reductase and dihydropteroate synthase genes in Senegal. *Trop Med Int Health* 2005; **10**: 1176-9.
184. Lucchi NW, Okoth SA, Komino F, et al. Increasing prevalence of a novel triple-mutant dihydropteroate synthase genotype in Plasmodium falciparum in western Kenya. *Antimicrob Agents Chemother* 2015; **59**: 3995-4002.
185. Mombo-Ngoma G, Oyakhirome S, Ord R, et al. High prevalence of dhfr triple mutant and correlation with high rates of sulphadoxine-pyrimethamine treatment failures in vivo in Gabonese children. *Malar J* 2011; **10**: 123.
186. Khalil IF, Ronn AM, Alifrangis M, et al. Response of Plasmodium falciparum to cotrimoxazole therapy: relationship with plasma drug concentrations and dihydrofolate reductase and dihydropteroate synthase genotypes. *Am J Trop Med Hyg* 2005; **73**: 174-7.
187. Ndiaye JLA, Ndiaye Y, Ba MS, et al. Seasonal malaria chemoprevention combined with community case management of malaria in children under 10 years of age, over 5 months, in south-east Senegal: A cluster-randomised trial. *PLoS Med* 2019; **16**: e1002762.
188. Chauvin P, Menard S, Iriart X, et al. Prevalence of *Plasmodium falciparum* parasites resistant to sulfadoxine/pyrimethamine in pregnant women in Yaounde, Cameroon: emergence of highly resistant pfdhfr/pfdhps alleles. *J Antimicrob Chemother* 2015; **70**: 2566–71.
189. Ako AAB, Johansson M, Traore R, et al. Sulphadoxine-Pyrimethamine resistant haplotypes in asymptomatically and symptomatically malaria infected individuals in Cote d'Ivoire. *Malar Chemother Control Elimination* 2014; **3**.
190. Smith SJ, Kamara ARY, Sahr F, et al. Efficacy of artemisinin-based combination therapies and prevalence of molecular markers associated with artemisinin, piperaquine and sulfadoxine-pyrimethamine resistance in Sierra Leone. *Acta Trop* 2018; **185**: 363-70.
191. Duah NO, Quashie NB, Abuaku BK, Sebeny PJ, Kronmann KC, Koram KA. Surveillance of molecular markers of Plasmodium falciparum resistance to sulphadoxine-pyrimethamine 5 years after the change of malaria treatment policy in Ghana. *Am J Trop Med Hyg* 2012; **87**: 996-1003.
192. World Wide Antimalarial Resistance Network (WWARN). Molecular Surveyor. http://www.wwarn.org/dhfr-dhps-surveyor/#0 (accessed July 04, 2018.

193. London School of Hygiene and Tropical Medicine. Drug resistance maps. Mapping the distribution of resistance genes of malaria in Africa. 2010. http://www.drugresistancemaps.org/ (accessed July 04, 2018.
194. Naidoo I, Roper C. Drug resistance maps to guide intermittent preventive treatment of malaria in African infants. *Parasitol* 2011; **138**: 1469-79.
195. Naidoo I, Roper C. Mapping 'partially resistant', 'fully resistant', and 'super resistant' malaria. *Trends Parasitol* 2013; **29**: 505–15.
196. Wang D, Hensman J, Kutkaite G, et al. A statistical framework for assessing pharmacological responses and biomarkers using uncertainty estimates. *Elife* 2020; **9**.
